# The cell cycle of *Leishmania*: morphogenetic events and their implications for parasite biology

**DOI:** 10.1111/j.1365-2958.2010.07479.x

**Published:** 2010-12-13

**Authors:** Richard J Wheeler, Eva Gluenz, Keith Gull

**Affiliations:** The Sir William Dunn School of Pathology, University of OxfordSouth Parks Road, Oxford OX1 3RE, UK

## Abstract

The cell cycle is central to understanding fundamental biology of *Leishmania*, a group of human-infective protozoan parasites. *Leishmania* have two main life cycle morphologies: the intracellular amastigote in the mammalian host and the promastigote in the fly. We have produced the first comprehensive and quantitative description of a *Leishmania* promastigote cell cycle taking a morphometric approach to position any cell within the cell cycle based on its length and DNA content. We describe timings of cell cycle phases and rates of morphological changes; kinetoplast and nucleus S phase, division and position, cell body growth and morphology changes, flagellum growth and basal body duplication. We have shown that *Leishmania mexicana* undergoes large changes in morphology through the cell cycle and that the wide range of morphologies present in cultures during exponential growth represent different cell cycle stages. We also show promastigote flagellum growth occurs over multiple cell cycles. There are clear implications for the mechanisms of flagellum length regulation, life cycle stage differentiation and trypanosomatid division in general. This data set therefore provides a platform which will be of use for post-genomic analyses of *Leishmania* cell biology in relation to differentiation and infection.

## Introduction

*Leishmania mexicana*, a protozoan parasite, is one of 21 known species responsible for leishmaniasis, a major human and animal disease in the tropics and subtropics in both the Americas and Afro-Eurasia (Herwaldt, 1999). In order to analyse fully the molecular mechanisms that govern *Leishmania* proliferation and control of morphology we need a rigorous quantitative description of the changes which occur to the cell during the cell cycle. For *Trypanosoma brucei* understanding of the division process has provided insights to pathogenicity features such as motility (Bastin *et al*., 2000; Broadhead *et al*., 2006), the flagellar pocket (Lacomble *et al*., 2009), differentiation from the long slender to the short stumpy bloodstream form in the mammalian host and subsequent differentiation to procyclic forms (Matthews *et al*., 1997) and their transformations to the epimastigote forms in the Tsetse fly (Sharma *et al*., 2008). To enable a similarly detailed analysis of the cell biology of *Leishmania* we have built a comprehensive, detailed description of the processes of cell division of *L. mexicana* which will facilitate future studies using the wide range of *Leishmania* genetic tools available (Beverley, 2003; Madeira da Silva *et al*., 2009; Damasceno *et al*., 2010) for analyses of *Leishmania* biology and pathogenicity.

*Leishmania mexicana* is a strong choice as a model *Leishmania* species. Both major life cycle stages [the promastigote in the sandfly and the amastigote in the mammalian host (Gossage *et al*., 2003; Antoine *et al*., 2004)] can be grown and differentiated *in vitro* under controlled conditions (Bates, 1994) and the genome is sequenced (*Leishmania mexicana* Genome Project, http://www.sanger.ac.uk/sequencing/Leishmania/mexicana/). We have used analysis of cells in exponentially growing populations combined with video microscopy of individual cells to produce a quantitative description of the *L. mexicana* promastigote cell cycle. This article provides the first detailed description of morphological changes during the cell cycle of a *Leishmania* species and expands on previous descriptions of nucleus and kinetoplast DNA synthesis and division. We have shown *L. mexicana* cell shape depends on its cell cycle stage and all morphologies present in a logarithmically growing population are part of a single proliferative cell cycle. Furthermore we have shown the *L. mexicana* flagellum growth extends over more than one cell cycle; hence a flagellum grows progressively longer every cell cycle. We have also calculated the timings of major cell cycle events; kinetoplast and nucleus division, DNA synthesis, basal body duplication and onset of new flagellum growth. These events and timings lead to an apparently heterogeneous range of cell morphologies in an exponentially growing culture population that can be understood through this new appreciation of the normal proliferative cell cycle.

## Results

### Logarithmic culture morphologies and the cell cycle

*Leishmania mexicana* promastigotes cultured *in vivo* progress through the cell cycle in an asynchronous manner and exponentially growing cultures contain cells with diverse morphologies. We reasoned that the range of individual cell morphologies that we observed in *L. mexicana* cultures were a reflection of different stages of the cell cycle. Our aim was to produce a description of the order and timing of the cell cycle events which produce this series of morphologies.

**Fig. 1:**
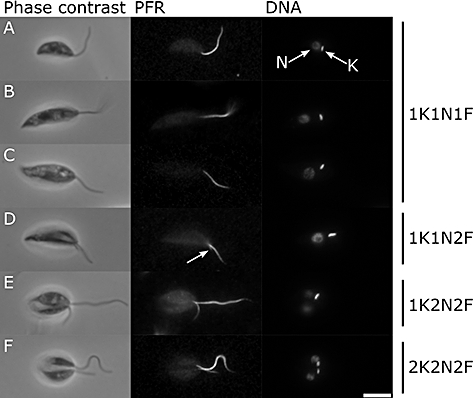
The cell cycle of promastigote *L. mexicana* by light microscopy. Micrographs of major cell cycle stages; cells were ordered based on number of kinetoplasts (K), nuclei (N) and flagella (F). The flagellum was labelled with the monoclonal antibody L8C4 which detects the PFR. Arrowed in (D) is the short new flagellum. Nuclear and kinetoplast DNA were labelled with DAPI. The kinetoplast and nucleus are indicated in (A). The scale bar represents 5 µm.

The flagellum, kinetoplast and nucleus provide discrete morphological markers. As is typical of trypanosomatids *L. mexicana* possesses a single nucleus (N), kinetoplast (K) and flagellum (F) and each replicates once during the cell cycle. Using 4′,6-diamidino-2-phenylindole (DAPI) staining of DNA and phase-contrast microscopy assisted by anti-paraflagellar rod (PFR) immunofluorescence to identify flagella the K/N configuration and occurrence of flagella was analysed. The expected morphologies 1K1N1F, 1K1N2F, 1K2N2F and 2K2N2F were observed (Fig. 1A–F). Given that these organelles duplicate once during the cell cycle this indicates that flagellum growth initiates first, followed by mitosis then kinetoplast division which starts after the onset of nuclear anaphase.

**Fig. 2:**
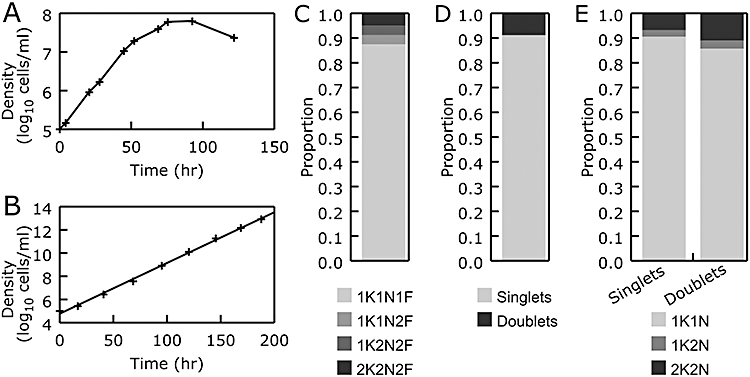
Logarithmic culture properties of *L. mexicana*.A. Growth curve of *L. mexicana* promastigotes at 28°C in M199 with 10% FCS, pH 7.4. Promastigotes grew logarithmically in the range 1 × 10^5^ to 1 × 10^7^ cells ml^−1^. Population growth slowed at densities of greater than 1 × 10^7^ cells ml^−1^ and ceases at approximately 8 × 10^7^ cell ml^−1^.B. Continued logarithmic growth of *L. mexicana*. Using repeated subculture to maintain promastigotes between 1 × 10^6^ and 1 × 10^7^ cells ml^−1^ gives rise to continuous logarithmic growth with a doubling time of 7.1 h.C. The proportions of nucleus (N), kinetoplast (K) and flagellum (F) configurations in cells undergoing logarithmic growth, *n* = 980.D. Approximately 10% of cells in logarithmic culture are connected via their posterior ends (doublets). The remainder are found as single cells (singlets), *n* = 1114.E. The proportions of nucleus and kinetoplast configurations for singlets and doublets are similar suggesting doublets progress through the cell cycle normally. Both cells in a doublet are universally found in the same K/N configuration. For singlets *n* = 942, for doublets *n* = 182.

Given the length of the cell cycle, the proportion of cells with each K/N/F configuration and the order of initiation of flagellum growth, mitosis and kinetoplast division the time of these events within the cell cycle can be calculated (Williams, 1971). We analysed exponentially growing *L. mexicana* promastigote cultures in order to determine the doubling time of the population and hence the length of the cell cycle. *L. mexicana* promastigotes grew logarithmically for densities under 1 × 10^7^ cells ml^−1^ (densities under 1 × 10^5^ were not tested). Population growth continued at a reduced rate up to approximately 8 × 10^7^ cells ml^−1^ (Fig. 2A). With repeated subculture to maintain the density between 1 × 10^6^ and 1 × 10^7^ cells ml^−1^ the population remains in logarithmic growth with a doubling time of 7.1 h (Fig. 2B). We then determined the proportion of cells in the population exhibiting each of the K/N/F configurations described above (Fig. 2C). From this analysis we calculated timings both in hours since the start of the 7.1 h long cell cycle and as the fraction of progress through the unit cell cycle, on a scale of 0–1, at which the event occurs (shown with the unit ‘u’). K, N and F duplication all occur late in the cell cycle; the new flagellum emerges from the flagellar pocket at 5.2 h (0.83 u) (i.e. 5.9 h through the 7.1 h cell cycle and 0.83 u of the unit cell cycle), DNA segregation during mitosis at 6.3 h (0.88 u) and kinetoplast division at 6.6 h (0.93 u).

**Fig. 3:**
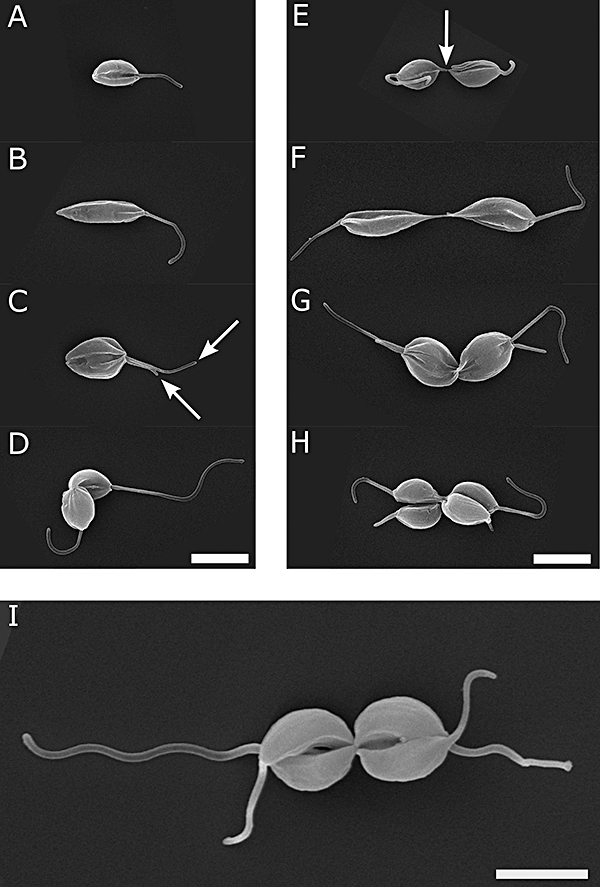
Promastigote *L. mexicana* by scanning electron microscopy.A–D. Micrographs showing major morphologies of separated individual cells. (C) shows a cell with two flagella (arrows), (D) shows very late cytokinesis.E–H. Micrographs showing major morphologies of doublets which approximately correspond to cell morphologies in (A)–(D). The posterior-to-posterior connection is arrowed in (E). Note that the cells shown in (D) and (E) are morphologically indistinguishable and whether they would remain attached or undergo abscission is not clear.I. A quadriflagellate doublet with a clear view of four flagella of different lengths.The scale bars represent 5 µm.

There is a population of cells which are found attached posterior-to-posterior by a thin cytoplasmic bridge. These are a common occurrence in culture and make up approximately 10% of the population (Fig. 2D, Fig. 3A–D compared with Fig. 3E–H). These cells, which we call doublets, appear to have failed to complete abscission but have otherwise completed organelle division. Doublets are distinct from previously described ‘rosettes’ which are larger clusters of cells positioned side-to-side with the flagellum towards the centre of the cluster (Dwyer *et al*., 1974). DAPI-stained doublet cells were examined for their K/N configurations. The two cells in a doublet universally appeared in the same K/N configuration as each other indicating that they progress through the cell cycle in synchrony. The proportions of doublets with 1K1N, 1K2N and 2K2N configurations were similar to those seen for separated individual cells (Fig. 2E). This indicates that doublets are progressing through the cell cycle in a similar manner to separated individual cells. As doublets appear to be going through the cell cycle normally they were treated as two separate cells for all further analyses.

This initial analysis established the basic order and timings of major morphological changes. Importantly in *L. mexicana* these events are concentrated in the last 20% of the cell cycle leaving the challenge of assessing whether other information can be used to chart the position of a cell in earlier stages of the cell cycle.

### Morphometric analysis of *L. mexicana*

**Fig. 4:**
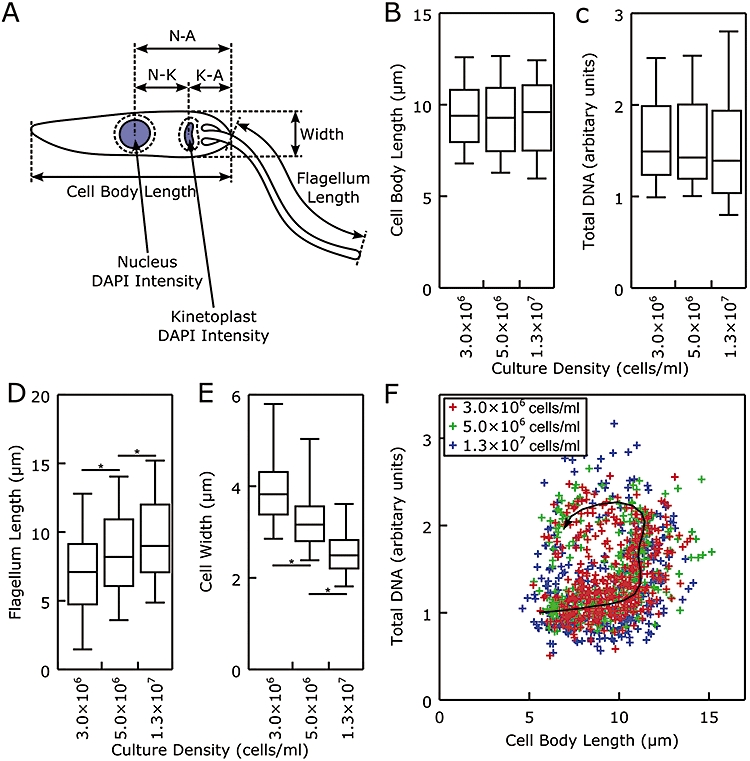
Basic of morphology analysis of *L. mexicana*.A. A cartoon showing the properties of each cell measured for analysis; cell body length and width, kinetoplast and nucleus DAPI intensity, flagellum length and kinetoplast–anterior (K–A), nucleus–anterior (N–A) and nucleus–kinetoplast (N–K) separation.B–E. Cell body length and total DNA content of *L. mexicana* does not vary for densities in the range 3.0 × 10^6^ to 1.3 × 10^7^ cells ml^−1^ following growth from subculture to 1.0 × 10^6^. Flagellum lengths tend to be longer at higher densities. Cell widths tend to be lower at high densities. Boxes and error bars indicate the median, upper and lower quartiles and 95th percentiles. Stars indicate significant differences (*P* < 0.01, Student's *t*-test), no other differences are significant.F. Scatter plot of cell body length against total DNA content. Each data point represents one cell. Three cultures at three different densities were analysed, the different colours indicate the culture density for each data point. A best-fit line (black arrow) was used to generate an estimate of cell cycle progression for each cell. 3.0 × 10^6^ cells ml^−1^ data: *n* = 320, 5.0 × 10^6^ cells ml^−1^ data: *n* = 341, 1.3 × 10^7^ cells ml^−1^ data: *n* = 321.

Having analysed the order and timing of duplication of the kinetoplast, nucleus and flagellum we then sought to place them within the context of variations of cell shape and form. We analysed the parameters of cell body length, width, flagellum length and kinetoplast and nuclear DNA content (see Fig. 4A). Measurements were made on cells taken from cultures at three different densities to check for variation of these properties in cultures across the logarithmic growth range: 3.0 × 10^6^, 5.0 × 10^6^ and 1.3 × 10^7^ cells ml^−1^. The distributions of cell body length, width, flagellum length and DNA content are shown in Fig. 4B–E. All raw morphometric data are given in Table S1. Cell body length has a wide distribution, from 6 to 12 µm with no significant difference between culture densities. Similarly total DNA content has a twofold range with no significant difference between culture densities. Flagellum length (typically 5–13 µm) increases and cell width (typically 2–5 µm) decreases with density (Fig. 4D and E) although flagellum length shows no correlation with cell width (Fig. S1A).

With this data set of morphological measurements of 980 individual cells we were then able to analyse the positioning of the kinetoplast and nucleus within each cell (see Fig. 4A). We found that the kinetoplast is positioned at a constant distance of approximately 2.5 µm from the anterior end of the cell. Nuclear position is variable but defined in that it shows a clear correlation with cell length. This relationship can be expressed as *n* ≈ 2.5 + 0.2*l* where *n* = anterior–nucleus distance and *l* = cell length in micrometres (Fig. S1B).

### Defining progress through the unit cell cycle and analysing morphological changes

The DNA replication cycle to generate duplicated DNA content for the two daughter cells is a key feature of the cell cycle. Analysis of cellular DNA content therefore provides a tool for placing a cell within the cell cycle. We noticed that there was also an approximately twofold range in cell lengths within the asynchronous population suggesting that the cell length increases progressively throughout the cell cycle. We conjectured that cell body length, like DNA content, may be a useful parameter for positioning a cell within the cell cycle. We therefore designed a method to use the combined morphometric data on DNA content and cell length to place each cell unambiguously at a position through the cell cycle. Using this calculated cell cycle progress the variation of other cell properties through the cell cycle can be analysed.

Our calculation of cell cycle progress from total DNA content and cell body length is based on a scatter plot of these two parameters (Fig. 4F). We used a stepwise path walking algorithm (see *Experimental procedures*) to draw a best-fit curve through the data starting from short cells with a unit amount of DNA. This best-fit curve describes the progression of an idealized cell through its cell cycle. A cell initially grows in length with a constant DNA content (G1), DNA synthesis then starts and the DNA is duplicated while the cell length remains constant (S phase). Finally the cell reduces in length, keeping duplicated DNA content (G2 and mitosis) before cytokinesis returns the cell to the start of the cell cycle. This quantitative analysis of cell length is consistent with the ordering of cell morphologies as seen qualitatively by microscopy (Fig. 1). The density of points around a section of the best-fit line (Fig. 4F) is related to the time spent at that stage of the cell cycle, i.e. G1 and S phase with a high point density are relatively slow while the low point density around regions with duplicated DNA indicates a short G2 phase and relatively fast mitosis and cytokinesis. Details of the calculations are given in *Experimental procedures*.

**Fig. 5:**
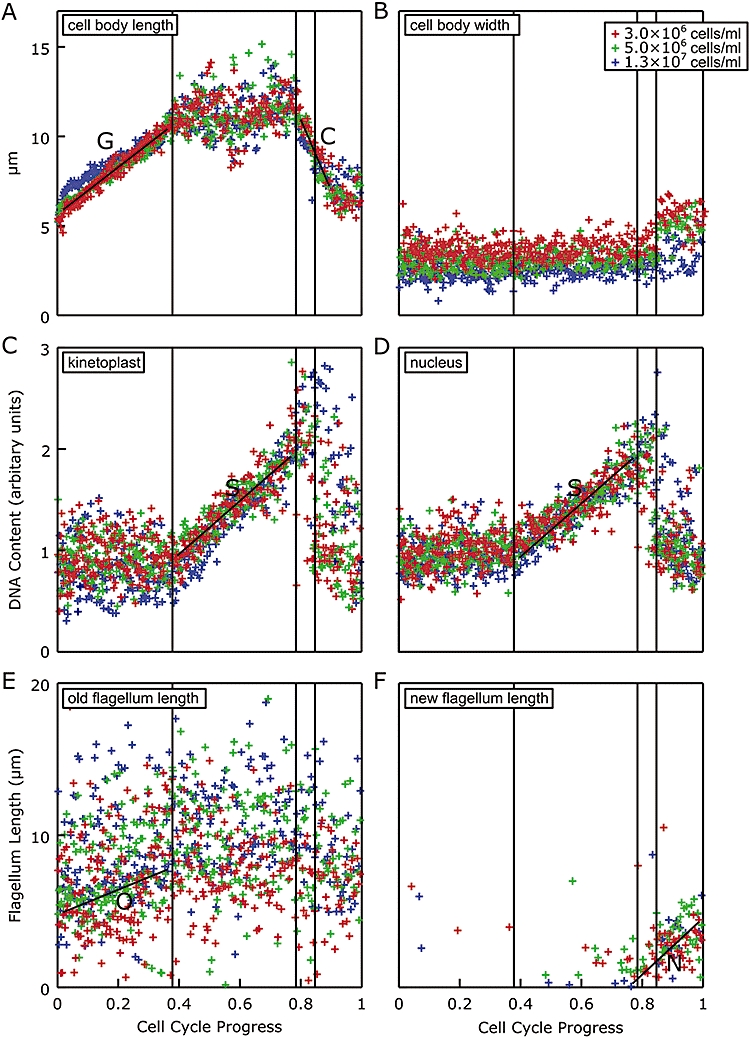
Morphological variables plotted against an estimate of cell cycle progression.A and B. Scatter plots of cell length and width against calculated cell cycle progress. Each data point represents one cell. Colours indicate the culture density for each data point. This plot outlines the changes of the cell body shape over the course of the cell cycle. The rate of increase of cell length during G1 (≈ 1.7 µm h^−1^) and decrease in cell length during post-S phase (≈ 10 µm h^−1^) are indicated with black lines labelled G and C respectively. On average cells from a lower culture density have a larger width. Cells from all culture densities show an increase in width around the onset of mitosis and cytokinesis.C and D. Scatter plots of kinetoplast and nuclear DNA content against calculated cell cycle progress. These plots show the timings and rates of DNA synthesis. The S phases of the kinetoplast and nucleus are synchronous and the rate of DNA synthesis is indicated with the black lines labelled S.E and F. Scatter plots of old and new external flagellum length against calculated cell cycle progress. New flagellum growth starts towards the end of the cell cycle, the relationship between the old flagellum length and the cell cycle is complex. The rate of old flagellum growth during G1 (≈ 1 µm h^−1^) and new flagellum growth during the late cell cycle (≈ 3 µm h^−1^) are indicated with black lines labelled O and N respectively.The three vertical lines present in all plots indicate timings of major features, from left to right: the end of cell length growth and the start of DNA synthesis; the start of cell length contraction and new flagellum growth; and mitosis and cell width increase. 3.0 × 10^6^ cells ml^−1^ data: *n* = 320, 5.0 × 10^6^ cells ml^−1^ data: *n* = 340, 1.3 × 10^7^ cells ml^−1^ data: *n* = 320.

This method for calculating cell cycle progress for each cell (i.e. placing each cell precisely within the unit cell cycle) is useful as it allowed us to analyse the morphometric data further. Specifically, it allowed us to calculate the rates of change in cell length and DNA content and analyse changes in flagellum length and cell width through the cell cycle. Figure 5A–F shows the changes in cell properties over the course of the cell cycle with the calculated cell cycle progress along the *x*-axis. These plots are powerful analysis tools for the investigation of gradual changes of morphology through the cell cycle.

From the plot of cell body length and width against calculated cell cycle progress (Fig. 5A and B) we calculated that the cell body grows in length from 6 µm at the start of the cell cycle to 11 µm at 2.7 h (0.38 u) at a rate of approximately 1.7 µm h^−1^. Cell length then remains constant from 2.7 h (0.38 u) to 5.6 h (0.78 u) then decreases rapidly from 5.6 h (0.78 u) to 6.4 h (0.9 u) at a rate of approximately 10 µm h^−1^. This returns the cell body length to 6 µm. Cell width remains constant (at a density-dependent value, see also Fig. 4E) from the start of the cell cycle to 6.4 h (0.9 u). At this point it increases to approximately 5 µm, this is synchronous with the decrease in cell length. Modelling the cell as a scalene spheroid showed that the increase in width and decrease in length observed during mitosis/early cytokinesis keep the volume (≈ 400 µm^3^) and surface area (≈ 320 µm^2^) approximately constant.

Plotting DNA content against calculated cell cycle progress (Fig. 5C and D) showed that DNA content of both the kinetoplast and nucleus remain constant from the start of the cell cycle to 2.7 h (0.38 u). From 2.7 h (0.38 u) to 5.6 h (0.78 u) DNA content increases linearly from one to two units. The increase of kinetoplast and nuclear DNA content occurs at the same time indicating their S phases are synchronous. Mitosis, identified by segregation of the DNA during anaphase, follows almost immediately on from the end of S phase. Kinetoplast division closely follows nuclear anaphase. There is no prolonged G2 phase.

Flagellum length showed a much less clear correlation with cell cycle progress (Fig. 5E and F). Looking at the length of the old flagellum (present from the start of the cell cycle) we found that cells at every stage in the cell cycle had a large range of old flagellum lengths, typically 5–15 µm. The plot shows a hint of growth of the old flagellum during G1 (a rate of around 1 µm h^−1^) (Fig. 5E). The shorter (new) flagellum emerges from the cell between 5.0 h (0.7 u) and 5.7 h (0.8 u) (near the end of S phase) and grows to a length of 4–5 µm at division at a rate of approximately 3 µm h^−1^. While number and length of flagella was not used to calculate cell cycle progress (only cell body length and total DNA content were used) our plot placed 90% of cells with two flagella in the last 2.9 h (0.4 u) of the cell cycle. This corroborates our method of using DNA content and cell length for placing cells along the unit cell cycle.

### Validation of cell cycle morphogenesis data

Our method of placing any cell from an asynchronous population at a particular stage of the cell cycle based on its length and DNA content is a powerful tool for analysis of gradual changes in cell shape, flagellum length and kinetoplast and nuclear DNA content and cytokinesis. We corroborated these findings further using a set of independent approaches; namely three methods of S phase analysis and time-lapse observations of single cells to assess cell shape. These further data rehearsed below were in full agreement with the analyses above.

From the cell cycle progress model the calculated durations of pre-S phase, S phase and post-S phase were 2.7 h (0.38 u), 2.9 h (0.4 u) and 1.6 h (0.22 u) respectively (Fig. 5C and D and Table 1). We used three different methods to support these estimates independently: (i) histogram analysis of total DNA content measurements, (ii) pulse-labelling of replicating DNA with BrdU and (iii) calculation of the post-S period based on K/N counts. In the first approach a histogram of total cellular DNA content was fitted to a curve to give a measurement of the number of cells in pre-S phase, S phase and post-S phase. This method was applied to the DAPI signal intensity data from the microscopy morphometric analysis (Fig. 4C) and to propidium iodide signal from flow cytometry; both give consistent S phase length and timing results (Table 1, Fig. S2). In the second approach short-pulse (15 min) labelling of synthesizing DNA with BrdU only labelled 1K1N cells. Assuming that there is no prolonged G2 phase the timing of S phase can be calculated from the proportion of labelled cells (Table 1). Third, assuming that S phase is complete before the onset of mitosis, the minimal duration of post-S phase can be calculated from the proportion of 1K2N and 2K2N cells in the population (Table 1). The values obtained by these three methods are in good agreement with the estimates based on the cell cycle progress model and taken together support the conclusion that S phase in *L. mexicana* lasts for 0.4 u of the cell cycle.

**Table 1:** Comparison of estimates of S phase by different methods

	Cell cycle progress model (1)	Microscope cytometry (2a)	Flow cytometry (2b)	K/N configuration counts (3)	Short-pulse BrdU (4)
Pre-S phase	2.7 h (0.38 u)	2.9 h (0.40 u)	2.6 h (0.37 u)	n/c	2.5 h (0.35 u)
S phase	2.9 h (0.40 u)	3.3 h (0.45 u)	3.25 h (0.46 u)	n/c	3.5 h (0.49 u)
Post-S phase	1.6 h (0.22 u)	1.0 h (0.14 u)	1.3 h (0.18 u)	1.2 h (0.17 u)	1.1 h (0.16 u)
*n*	980	980	10 000	980	1053

The length of pre-S phase, S phase and post-S phase as determined by four methods: (1) pre-S phase, S phase and post-S phase lengths as determined from Fig. 5C, D. (2) Fitting of pre-S phase, S phase and post-S phase to a histogram of DNA content, performed on microscopy (DAPI stained) and flow cytometry (propidium iodide stained) measurements of cellular DNA content. (3) The minimum length of post-S phase based on the proportion of 1K2N and 2K2N cells; kinetoplast and nuclear division can only occur after the end of S phase. As this minimum bound is similar to other post-S phase measurements this confirms G2 is short. (4) On the assumption that there is no prolonged G2 phase the proportion of BrdU positive 1K1N cells following a short (15 min) BrdU pulse can also be used to calculate the length of pre-S phase and S phase, the proportion of 1K2N and 2K2N cells give the length of post-S phase. n/c indicates the timing for the phases are not calculable with that experimental method.

**Fig. 6:**
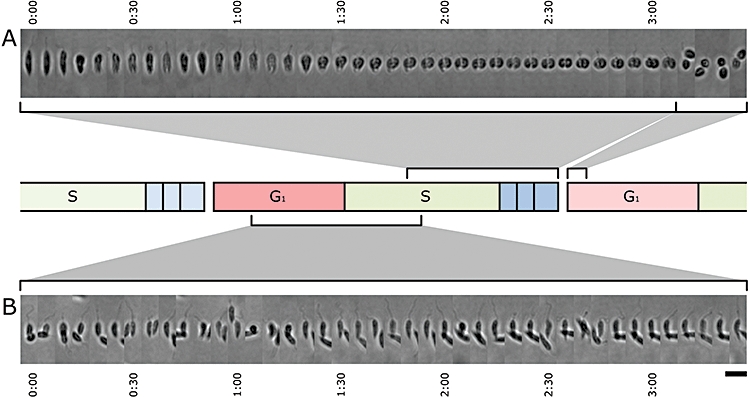
Time-lapse observation of single cells progressing through the cell cycle. Cells were trapped in 0.5% agarose to assist observation and a *z*-stack was captured every 1 min. The most in-focus slice was selected and rotated for every time point to give a constant cell orientation, one in five images (i.e. one image every 5 min) is shown here.A. A cell contracting in length and increasing in width to give a bi-lobed cytokinetic morphology before abscission. The two daughter cells remain trapped in the same liquid pocket within the agarose following division.B. Two cells, likely to be sister cells, growing in length. Both cells are growing in length at the same rate.For both (A) and (B) the brackets indicate the approximate position in the cell cycle (centre) of the cells in the time-lapse images. The scale bar represents 10 µm.

The best independent support for any cell cycle analysis is consistency with time-lapse observations of single cells. We were able to perform long term time-lapse microscopy on motile cells by embedding *L. mexicana* cells in pockets within 0.5% low-melt agarose. Micrographs were captured every minute for 12 h. Flagellum movement was seen to slow after 5 h so only the first 3.5 h (approximately half the cell cycle) following embedding was analysed to ensure minimal perturbation of the cell cycle due to the unusual cellular environment. We observed many cells at different stages of the cell cycle, including cells undergoing cytokinesis and division (Fig. 6A). These direct observations of cytokinesis and length and width changes were consistent with the data in Fig. 5 confirming that cells grow in length through G1 (Fig. 6B), resulting in a doubling of length, and undergo rapid length reduction and width increase ≈ 1.3 h prior to division (Fig. 6A). These shape changes can also be seen in the form of time-lapse video (see Video S1).

### Asymmetries in division

**Fig. 7:**
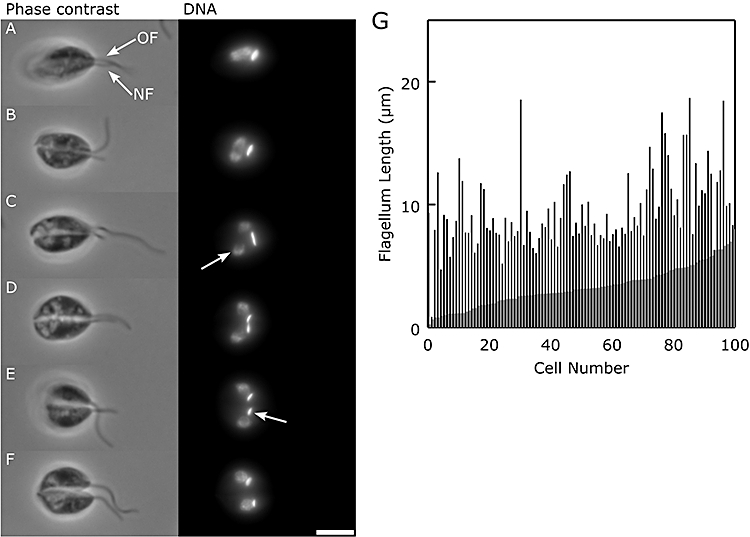
Asymmetries in promastigote *L. mexicana* division.A–F. Micrographs of cells during mitosis, kinetoplast division and cytokinesis arranged in order of cell cycle progress. Phase-contrast images are a single slice from a *z*-stack, DNA images show DAPI staining averaged over all slices of the *z*-stack. The kinetoplast and nucleus enter division on the old flagellum (OF) one side of the cell (A). During mitosis one nucleus is repositioned to the new flagellum (NF) side of the cell, this nucleus lies further towards the posterior end (left) of the cell (C, arrow). One daughter kinetoplast is positioned on the new flagellum side of the cell and initially lies perpendicular to its partner nucleus (E, arrow). All cells are orientated with the new flagellum on the lower half of the cell. The scale bar represents 5 µm.G. Bar graph showing old (black) and new (grey) flagellum length for 100 cells with two flagella (representative of 296 cells). Cells were arranged in order of increasing new flagellum length and the pairs of flagellum length were plotted. The old flagellum length (plotted next to the corresponding new flagellum) shows flagellum length variability and no clear relationship to new flagellum length.

During microscopy analysis of morphology some asymmetries in division were noticed. While cytokinesis appears to be longitudinally symmetric (unlike in *T. brucei*) the positioning of the kinetoplast and nucleus leading up to and during mitosis and kinetoplast division is not (Fig. 7A–F). Light microscopy showed that a 1K1N cell enters mitosis with the kinetoplast and nucleus asymmetrically positioned on the same side of the cell as the long (old) flagellum. Mitosis results in the positioning of one of the daughter nuclei to the other (new flagellum) side of the cell at a more posterior position. Kinetoplast division occurs similarly with the daughter kinetoplast associated with the short (new) flagellum moving to join the daughter nucleus on the other (new flagellum) side of the cell. Cytokinesis then proceeds symmetrically along the longitudinal axis of the cell (Fig. 7A–F).

As seen in Fig. 5E flagellum length has a large range and a complex relationship with the cell cycle. To provide a simpler data set for analysis of flagellum dynamics the lengths of flagella of cells undergoing new flagellum growth (i.e. all cells with two flagella) were measured. The flagella length pairs were plotted in order of increasing new flagellum length (Fig. 7G), as was used to analyse new flagellum growth in *T. brucei* (Tyler *et al*., 2001). Figure 7G illustrates in more detail the events during the last 20% of the cell cycle as seen in Fig. 5E and F. Pair-wise analysis of new and old flagellum length showed that at the point of division the old flagellum is always longer than the new flagellum i.e. there is an asymmetry in flagella lengths. Most surprisingly old flagellum length showed great variability and therefore had not reached a rigidly defined length by the time a cell re-entered division. Figure 7G also allows an estimate of new flagellum growth rate. At division the external portion of the new flagellum measured 5 µm and we showed above that growth of the external portion of the new flagellum occurs during the last 1.2 h of the cell cycle (Fig. 2C). This suggests a growth rate of approximately 4 µm h^−1^ which is in good agreement with the 3 µm h^−1^ estimate derived from our cell cycle progression model (Fig. 5F).

### Basal body duplication and reorientation

**Fig. 8:**
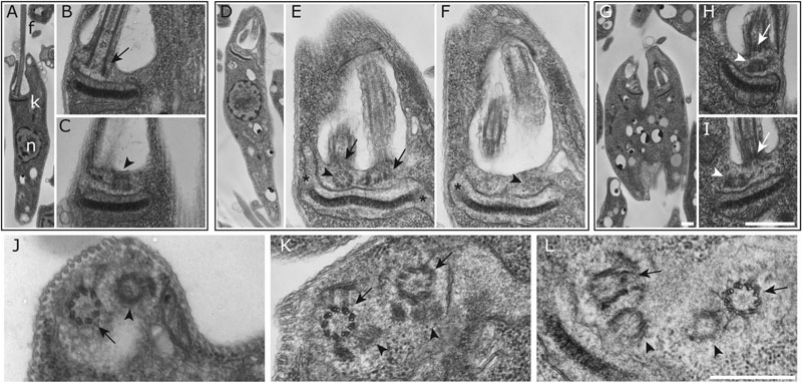
The basal body duplication cycle. Electron microscopy of serial thin sections resolved the order of events in basal body duplication and early stages of new flagellum growth.A–C. Longitudinal sections through a 1K1N cell; the kinetoplast in this cell is associated with one basal body (B, arrow) subtending the single flagellum and one pro-basal body (C, arrowhead).D–F. Longitudinal sections through a 1K1N cell. The kinetoplast is elongated and filamentous lobes at each pole (marked with asterisks in E and F) indicate that it is in S-phase. This cell has two basal bodies (E, arrows), one at the proximal end of the old flagellum and one at the proximal end of the short new flagellum (still inside the flagellar pocket, Fig. S4). Two pro-basal bodies are positioned orthogonal to the basal bodies (E and F, arrowheads).G–I. Longitudinal sections through a 2K2N cell; both kinetoplasts in this cell are associated with one basal body (H and I, arrows) and one pro-basal body (H and I, arrowheads).J–L. Cross-sections of basal body/pro-basal body pairs.f, flagellum; k, kinetoplast; n, nucleus. Micrographs are shown at one of three magnifications; scale bar in (G) represents 1 µm (A, D and G are at the same magnification); scale bar in (I) represents 500 nm (B, C, E, F, H and I are at the same magnification) and scale bar in (L) represents 500 nm (J–L are at the same magnification).

Measurements of new flagellum lengths were performed only on the cell-external portion of the flagellum, which first emerged near the end of S phase. Examination of serial sections by TEM was used to resolve the timings of new flagellum growth initiation and formation and re-orientation of the pro-basal bodies, relative to the other cell cycle markers (Fig. 8). All 1K1N1F cells possessed one basal body at the proximal end of the flagellum with an adjacent pro-basal body (*n* = 7 cells; Fig. 8A–C and J and Fig. S3). In 1K1N2F cells where the short new flagellum was still fully contained within the flagellar pocket, both basal bodies had an orthogonally positioned pro-basal body next to it (*n* = 9 cells; Fig. 8D–F and K, Figs S4 and S5). In cells with a dividing kinetoplast and mitotic nucleus, the pro-basal bodies had re-oriented such that they lay parallel to the basal bodies (Fig. 8L and Fig. S6; *n* = 9 cells); at cytokinesis, each daughter inherits one basal body/pro-basal body pair (Fig. 8G–I; *n* = 5 cells). These data show that the basal body duplication cycle of *L. mexicana* follows a pattern similar to that seen in *T. brucei* (Sherwin *et al*., 1989) and *T. cruzi* (Elias *et al*., 2007).

These electron micrographs indicate that the earliest new flagellum growth begins during kinetoplast S phase (Fig. 8D–F) but the flagellum only becomes visible outside of the flagellar pocket by light microscopy near the end of S phase. By this stage the new flagellum has extended through the approximately 2-µm-deep flagellar pocket and we estimate that it may have grown an additional 1–2 µm before becoming clearly visible next to the base of the old flagellum. Given a growth rate of 3–4 µm h^−1^ (Figs 5F and 7G) we therefore estimate that basal body maturation and onset of axoneme growth occurs approximately 1 h before the end of S phase.

### Modelling flagellum growth

**Fig. 9:**
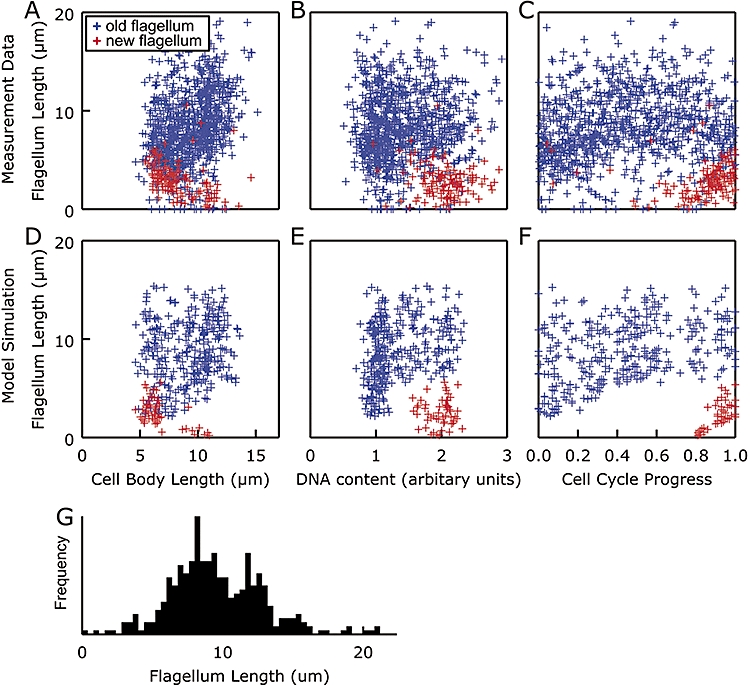
Modelling of population flagellum length distributions.A–C. Cell body length, DNA content and cell cycle progress show complex correlations with flagellum length. *n* = 980.D–F. A simple model of flagellum growth based on the balance-point model where flagellum growth is driven by a cytoplasmic pool of flagellum components produced throughout the cell cycle (except S phase) give distributions that match the experimental data.G. A histogram of flagellum length of all cells during S phase (from the experimental data). The model of flagellum growth predicts a multi-modal distribution of S phase cells' flagellum lengths. The histogram of measured flagella lengths of S phase cells shows three peaks and confirms this prediction. *n* = 360.

Unlike cell body length, width, DNA content and organelle number the old flagellum length did not show a straightforward correlation with the cell cycle (see Figs 5E and 7G). One likely explanation is that the flagellum grows over more than one cell cycle and that the rate of extension varies over this period. The different flagella lengths on dividing doublet cells, which possess four sibling flagella, support this presumption; the two flagella formed during the current cell cycle are very short and approximately equal in length while the remaining two, one of which was formed the previous cell cycle and one earlier, are not equal in length (Fig. 3I). In order to analyse the complex relationships between flagellum length and total DNA content, cell length and cell cycle progress (Fig. 9A–C) we developed a computer model of flagellum growth with the aim of identifying from our data the cell cycle stages where the flagellum is undergoing growth.

Our simple simulation of flagellum growth was based on the balance-point model (Marshall *et al*., 2005), a model of flagellum length control, which states that a flagellum grows longer at a rate inversely proportional to its length. Slow flagellum length-independent disassembly also occurs at the flagellum tip. Once the flagellum reaches a certain length the growth and disassembly rates balance giving rise to an equilibrium length. We extended this model with the plausible assumption that flagellum growth is driven by the availability of a cytoplasmic pool of flagellum components. We adjusted the flagellum growth rate, disassembly rate and component synthesis rate to match the flagellum length distributions seen in Figs 4D and 7G (for more detail see *Experimental procedures*). We then simulated flagellum growth with flagellum component synthesis occurring at different stages of the cell cycle and the simulation results were compared with experimental data (Fig. 9A–C). Only one pattern of flagellum component synthesis matched the experimental data namely that flagellum component synthesis occurs throughout the entire cell cycle except S phase (Fig. 9D–F). In this model flagellum growth occurs during G1 and there is limited growth during early S phase. It also suggests there may be some decrease in old flagellum length during new flagellum growth and that new flagellum growth rate is increased if the old flagellum is particularly long.

Because the model entails that flagellum growth stalls during S phase (see Fig. 9F) a histogram of flagella lengths of cells in S phase would be expected to have a multi-modal distribution where each peak represents flagella which are one, two, three, etc. cell cycles old. We tested this by plotting a histogram of the flagella lengths we had measured for cells with a calculated cell cycle progress of 0.4–0.8 u (see Fig. 5C and D) which are in S phase. The resulting histogram showed a multi-modal distribution, as predicted by the model (Fig. 9G).

## Discussion

**Fig. 10:**
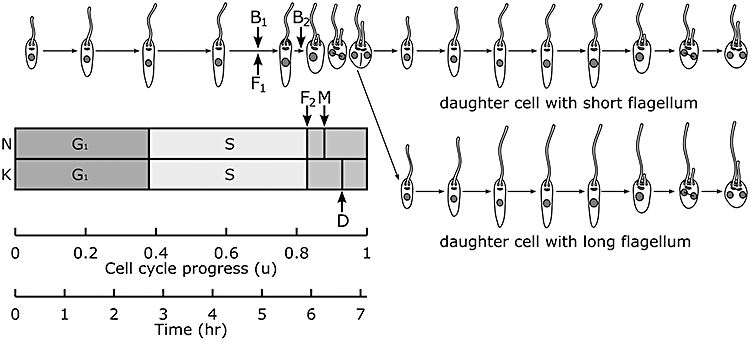
The cell cycle of promastigote *L. mexicana*. Top left: cartoons of the major morphological forms occurring during the cell cycle and their approximate timing. Cell length increases during G1, remains constant during S phase and decreases for division. Flagellum length increases during G1 and remains constant during S phase. The new flagellum emerges from the flagellar pocket at the end of S phase but flagellum length at division is not equal giving rise to one daughter cell with a short flagellum (top right) and one daughter cell with a long flagellum (bottom right). Both daughter cells continue through the cell cycle normally. The approximate timings of pro-basal body formation (B_1_), pro-basal body rotation (B_2_) and the start of axoneme extension from the basal body (F_1_) are indicated. Bottom left: summary of the timing of DNA synthesis and division of the nucleus (N) and kinetoplast (K). S phases of the kinetoplast and nucleus are synchronous and take up a large proportion of the cell cycle. M indicates DNA segregation during mitotic anaphase, D indicates kinetoplast division. F_2_ indicates the emergence of the new flagellum from the flagellar pocket.

Taken together our data provide a detailed description of the *L. mexicana* promastigote cell cycle which we summarize in Fig. 10. We established the timing of the successive phases of the nuclear and kinetoplast replication cycles and defined the changes in cell length and width that occur as a function of cell cycle progress. All of the diverse morphologies present in an exponentially growing culture are as a result of the cell cycle. We have also determined the approximate timing of basal body maturation and onset of axoneme extension, pro-basal body formation and pro-basal body reorientation. At division, the two daughter cells possess flagella of unequal length and our data suggest that the *L. mexicana* flagellum continues to increase in length over multiple cell cycles.

### The cell cycle of *L. mexicana*

We have shown that during the course of its cell cycle *L. mexicana* progresses through well-defined morphological stages in manner similar to other trypanosomatids (Cosgrove *et al*., 1970; Sherwin *et al*., 1989; Woodward and Gull, 1990;Tyler *et al*., 2001). The present study is the first comprehensive quantitative analysis of a *Leishmania* cell cycle and provides detail of timings of the cell cycle events and the rates of morphological changes which provides a foundation for analysis of *Leishmania* cell biology. This work builds upon earlier, albethey limited, separate studies which considered cell shape (Chakraborty *et al*., 1962) and DNA synthesis (Bhattacharya *et al*., 1985). In combination with existing detailed morphological descriptions of the cell cycle for *T. brucei* (Vaughan and Gull, 2008) and *T. cruzi* (Elias *et al*., 2007) greater comparative analysis of trypanosomatid division is now also possible.

Our data indicate that in *L. mexicana* nuclear and kinetoplast S phase occur simultaneously and occupy a large proportion (40%) of the cell cycle (Fig. 5C and D and Table 1). The *L. mexicana* nuclear S phase (2.9 h) appears particularly long in comparison with *T. brucei* (1.51 h) (Woodward and Gull, 1990), *Crithidia fasciculata* (1.35 h) (Cosgrove *et al*., 1970) and *T. cruzi* (2.4 h) (Elias *et al*., 2007). Given that the total nuclear DNA content of *L. mexicana*[around 64 Mb (*Leishmania mexicana* Genome Project)] is similar to that of *T. brucei* (60 Mb, 0.097 pg) (Borst *et al*., 1982; Berriman *et al*., 2005) and *C. fasciculata* (0.095 pg) (Borst *et al*., 1982) and approximately half that of *T. cruzi* (110 Mb, 0.156 pg) (Borst *et al*., 1982; Berriman *et al*., 2005), *L. mexicana* appears to take twice as long to duplicate the same amount of nuclear DNA. Whether this reflects differences in chromosome number and genome architecture (Van der Ploeg *et al*., 1984; Berriman *et al*., 2005; El-Sayed *et al*., 2005; Ivens *et al*., 2005) or different processivity of the DNA replication machinery remains to be determined.

In contrast the rate of accumulation of kinetoplast DNA (kDNA) in *L. mexicana* is more similar to other trypanosomatids. The *L. mexicana* kinetoplast S phase (2.9 h) is comparable to *T. cruzi* (2.4 h) (Elias *et al*., 2007) and *C. fasciculata* (0.98 h) (Cosgrove *et al*., 1970). The quantity of kDNA present in these species is also comparable; the quantity of kDNA in *L. mexicana*[calculated from approximate mini- and maxicircle size and number (Rogers *et al*., 1988)] is similar to *T. cruzi* (0.047 pg) (Henriksson *et al*., 1996) and is higher than that of *C. fasciculata* (0.032 pg) (Borst *et al*., 1982). These rates of accumulation of kDNA are much higher than that of *T. brucei* where 0.004 pg kDNA is duplicated in 0.99 h (Borst *et al*., 1982; Woodward and Gull, 1990). The kDNA minicircles in *T. brucei* have high sequence diversity (Steinert and Van Assel, 1980) and show a different pattern of accumulation during kDNA network replication (Guilbride *et al*., 1998); one of these factors may be linked to the slow rate of kinetoplast DNA synthesis. Mitosis is underway prior to the completion of kinetoplast segregation in *L. mexicana* (Fig. 1), unlike *C. fasciculata* (Cosgrove *et al*., 1970) and *T. brucei* (Woodward and Gull, 1990) where duplicated kinetoplasts segregate before the onset of mitosis.

Our analysis showed that at division *L. mexicana* promastigotes are first formed with a new flagellum that is often significantly shorter (up to 10 µm) than the existing older flagellum, whose length is highly variable (Fig. 7G) and continues to increase over multiple cell cycles. This is in sharp contrast to *T. brucei* where both the old and new flagellum lengths at division are within a narrow range (Tyler *et al*., 2001). While recent work has shown that there is some limited flagellum growth during G1 phase in *T. brucei* (Farr and Gull, 2009), this is to a far lesser extent than inferred here for *L. mexicana*, indicating different mechanisms of flagellar length regulation between these species. New flagellum growth rates are, however, not dramatically different; calculated to be 8 µm h^−1^ in *T. brucei* (Tyler *et al*., 2001) and estimated here at 3–4 µm h^−1^ in *L. mexicana*. The basal body duplication cycle of *L. mexicana* follows a similar pattern to that seen in *T. brucei* (Sherwin *et al*., 1989) and *T. cruzi* (Elias *et al*., 2007) and earliest onset of new flagellum growth occurs during the kinetoplast S phase.

While cell shape and the changes in cell shape for cytokinesis appear symmetrical mitosis and kinetoplast division are not. The asymmetries in mitosis and kinetoplast division of *L. mexicana* have interesting similarities with *T. brucei* (Robinson *et al*., 1995) and may reflect shared underlying mechanisms. Of *C. fasciculata, T. cruzi, T. brucei* and now *L. mexicana* only the procyclic-form *T. brucei* achieves a large kinetoplast separation during division and only procyclic *T. brucei* possesses a flagella connector (Briggs *et al*., 2004). We observed that during division of *L. mexicana*, abscission does not always occur following cytokinesis resulting in approximately 10% of the population remaining attached by a cytoplasmic bridge connecting their posterior ends. These doublets appear to go through the cell cycle normally. The *in vivo* relevance of this is unknown, it may indicate that effective motility is required to complete cytokinesis and is compromised in culture. Alternatively, in the sandfly gut it may provide a mechanism for enhancing the chance that the daughter of an attached promastigote has sufficient time to attach to the epithelium via its new flagellum or to be held longer before escape to the lumen in an asymmetric division.

### Implications for differentiation

In this article we have considered exclusively the proliferative cell cycle of one life cycle stage. However *Leishmania* are a parasitic species and undergo large morphogenetic changes between different life cycle stages in different regions within their hosts and vectors. In trypanosomatids life cycle differentiation events appear to involve a specialized division in which one or both daughter cells have an altered morphology and/or biochemistry. This is in contrast to normal proliferative cell division where both daughter cells are identical to their parent. Our study has implications for understanding the complexities of differentiation division transitions in the life cycle.

We have shown that *L. mexicana* promastigotes in exponential growth in culture progress through a range of morphologies which make up a single cell cycle. The subpellicular microtubule array and flagellum of *L. mexicana* have much plasticity of form; the subpellicular array can halve in length and double in width and the regulation of flagellum length appears, at least partially, decoupled from the cell cycle. Microtubule dynamics are therefore likely to have an interesting regulation in *Leishmania. L. mexicana* division is asymmetric and this may, as in *T. brucei* (Tyler *et al*., 2001; Sharma *et al*., 2008), be used as a tool for generating modified daughter cell morphology via specialized differentiation division. In the sandfly vector multiple different forms have been described (Rogers *et al*., 2002; Gossage *et al*., 2003) involving successive differentiation from amastigote form cells to procyclic, nectomonad, leptomonad then metacyclic promastigotes over the course of approximately three doublings of the population (Rogers *et al*., 2002). These sandfly promastigote forms have been defined by their morphology (flagellum length and cell body length and width). The possibility that differentiation and cell division are closely linked has been raised in several studies (Bates, 1994; Rogers *et al*., 2002) but the true relationships between the proliferative cell cycle and differentiation to the next life cycle stage remain unclear. Our results showing that promastigote morphology is dependent on cell cycle stage may influence interpretation of promastigote life cycle stage variations.

This study provides the first quantitative description of morphological events during the *L. mexicana* cell cycle. The division process of *L. mexicana* is fundamentally similar to other trypanosomatids and our detailed analysis of the *L. mexicana* cell cycle reported here provides another data set for the meta-analysis of trypanosomatid division, which may shed light on cellular processes common to this family of protists as well as processes specifically adapted to the biology of each species. We are now also in a position where the mechanisms of morphological change during *Leishmania* life cycle stage differentiation and their dependence on the cell cycle can be analysed in detail. The regulation of flagellum length (Bengs *et al*., 2005; Erdmann *et al*., 2006; Wilson *et al*., 2008; Casanova *et al*., 2009) and the diversity of flagellum function (Gluenz *et al*., 2010a,b;) are particularly interesting areas of *Leishmania* biology. Our insights into the promastigote cell cycle and flagellum length regulation in relation to it provide particular support for future study of *Leishmania* flagellum formation, growth and length regulation.

## Experimental procedures

### Cells

Promastigote-form *L. mexicana* (WHO strain MNYC/BZ/62/M379) were grown in M199 medium (Sigma) (10% FCS, pH 7.4, 28°C), and culture density was measured with a CASY model TT cell counter (Innovatis).

### Microscopy and morphometric measurements

For light microscopy cells were harvested, washed in PBS, settled on aminoalkylsilane-coated slides and fixed with 2% paraformaldehyde for 15 min. For quantitative DAPI staining slides were incubated in 1 µg ml^−1^ DAPI in water for 2 min, washed with PBS and mounted in glycerol with 1% 1,4-diazabicyclo[2.2.2]octane and 10% 50 mM sodium phosphate, pH 8.0. Images were captured on a DM5500 B epifluorescence microscope (Leica) with an Orca cooled CCD camera (Hamamatsu). Length and intensity (sum pixel intensity within an area) measurements were made in ImageJ (Collins, 2007). The morphological features measured were cell body length and width, nucleus and kinetoplast position, flagellum length and DNA content of the nucleus and kinetoplast. Anti-PFR immunofluorescence used L8C4 (Kohl *et al*., 1999) primary antibody with anti-mouse antibody-fluorescein isothiocyanate conjugate (Jackson Immunoresearch) secondary antibody. BrdU labelling of DNA undergoing synthesis was performed as described (Woodward and Gull, 1990) except cells were fixed in 0.1% paraformaldehyde. Scanning electron microscopy samples were prepared as described in Sharma *et al*. (2008). Images were captured on a JSM-6390 scanning electron microscope (JEOL) and prepared for publication in ImageJ. Transmission electron microscopy of serial sections was performed as described in Gluenz *et al*. (2010b).

### Live cell time-lapse microscopy

For live cell microscopy cells were immobilized by mixing of an equal volume of cells in culture medium with 1% low-melt agarose (Sigma, final concentration of 0.5% agarose) and allowing the agarose to solidify at approximately 20°C. A stack of 10 images was captured every 1 min with a DM550 B epifluorescence microscope (Leica) with an Orca cooled CCD camera (Hamamatsu) with a *z* spacing of 2 µm using a FCS3 heated chamber (Bioptechs) to maintain temperature at 28°C. In focus slices were selected, rotated and prepared for publication in ImageJ.

### Calculation of cell cycle timings

In an asynchronous logarithmic culture with multiple morphologies corresponding to different cell cycle stages the proportion of cells with each morphology is related to the timing of cell cycle events which give rise to the morphologies. This relationship is complicated by the growth of the population by binary fission. Simply put there are always twice as many cells just entering a new cell cycle as there are just leaving the previous cycle. This bias towards observing cells within an asynchronous population which are at an early stage in the cell cycle was accounted for as described in Williams (1971):


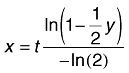


where *y* = cumulative proportion of cells up to and including that stage of the cell cycle, *t* = doubling time of the population and *x* = time through the cell cycle. If *t* = 1, x = progress through the unit cell cycle on a scale of zero to one, referred to here with a unit of ‘u’.

### Calculation of cell cycle progress using DNA content and cell length measurements

The stepwise path walking algorithm for curve fitting for cell cycle analysis was performed by a custom script in ImageJ (available on request). For each step the Gaussian weighted average (with variances equal to estimated variance in the DNA content and length data) of all data points within 90° of the current direction of travel gave the next point in the line. Variances were estimated from a Gaussian curve fitted to the histograms of DNA content and cell length, ±0.21 units and ±1.25 µm respectively. The fitted curve was allowed to complete a single loop. Cell cycle progress was calculated by assigning each data point to the nearest point on the fitted curve. The distance along the curve was calculated for each point, and all data points were ranked in ascending order of distance along the curve. The bias towards early cell cycle stages was accounted for with *y* = *r*/*n*, where *r* = rank of the current data point and *n* = number of data points. Calculations were performed for each culture density separately to give timings for each culture density independently. To ensure equal weighting of DNA content and length all calculations were performed on DNA content and length data normalized to the mean DNA content and mean length respectively.

### Flow cytometry and analysis of DNA content

Flow cytometry with propidium iodide DNA staining was performed as described in Signorell *et al*. (2009). A histogram of DNA content can be analysed to determine the lengths of pre-S phase, S phase and post-S phase. Histogram data were fitted to a curve made up of pre-S phase (single DNA content) and post-S phase (duplicated DNA content) linked by an S phase with a constant rate of DNA synthesis. Error in measurement was treated as Gaussian with variance proportional to DNA quantity. Number of cells in each category was adjusted for the binary fission bias to give S phase length and timing estimates.

### Flagellum length modelling

Flagellum length modelling was performed by custom scripts in Python (available on request). Changes in flagellum length and free flagellum component concentration were calculated for 100 steps per cell cycle. The cell body length and total DNA corresponding to progress through the cell cycle were inferred from the data in Fig. 5A–D. The model for flagellum length growth was based on the balance-point model (Marshall *et al*., 2005); the formulae were adapted for the purposes of this analysis. Flagellum growth and change in flagellum component pool concentration are given by:


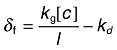



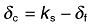


where: *δ*_f_ = change in flagellum length, *k*_g_ = flagellum growth rate constant, *k*_d_ = flagellum decay rate constant, *l* = flagellum length, [*c*] = flagellum component pool concentration, *δ*_c_ = change in flagellum component pool, *k*_s_ = flagellum component synthesis rate. A population of cells were simulated progressing through four generations with new flagellum growth allowed to start at 0.8 u.

In order to fit our model to the available data we adjusted four variables: (1) flagellum component synthesis rate, (2) flagellum growth rate constant, (3) flagellum disassembly rate and (4) the times during the cell cycle in which flagellum component synthesis is occurring. We started by adjusting the three rate constants (parameters 1–3) to match the distribution of flagellum lengths seen in Fig. 4D. Starting values for these variables were based on the following observations: flagellum component synthesis must occur at approximately one flagellum's-worth per cell cycle. Maximum flagellum length is ≈ 20 µm and the ratio of flagellum growth rate to disassembly rate defines the maximum flagellum length. Flagellum growth rate is slow compared with the length of the cell cycle, very few cells reach flagellum length equilibrium. This is shown by the small proportion of cells with long flagella on the flagellum length distribution (Fig. 4D) and variability of old flagellum length on 2F cells (Fig. 7G). We adjusted the values for these parameters until the flagellum length simulation matched the observations described above. The final values for used for these parameters were: flagellum component synthesis rate = 12 µm (cell cycle)^−1^, flagellum assembly rate constant = 0.014 h^−1^ and flagellum disassembly rate = 0.28 µm h^−1^. Once approximate values for the rate constants (parameters 1–3) were determined different timings of flagellum component synthesis (parameter 4) were tested. Flagellum component synthesis was simulated to occur in pre-S phase, S phase, post-S phase and combinations thereof. The best fit to the experimental data was achieved with flagellum component synthesis occurring during all non-S phase periods of the cell cycle.
