# Supplementary material for: The cell cycle of Leishmania: morphogenetic events and their implications for parasite biology
Source: Mol Microbiol. 2010 Dec 13;79(3):647–62. doi: 10.1111/j.1365-2958.2010.07479.x (PMC3166656; doi:10.1111/j.1365-2958.2010.07479.x)
Supplement: Supplementary file 1 [file mmi0079-0647-SD1.pdf]

The cell cycle of *Leishmania*: morphogenetic events and their implications for parasite biology

Richard J Wheeler, Eva Gluenz[1] and Keith Gull

The Sir William Dunn School of Pathology, University of Oxford, South Parks Road, Oxford, OX1 3RE. UK

[1]Corresponding author:eva.gluenz@path.ox.ac.uk +44 1865 285456

Supporting Information

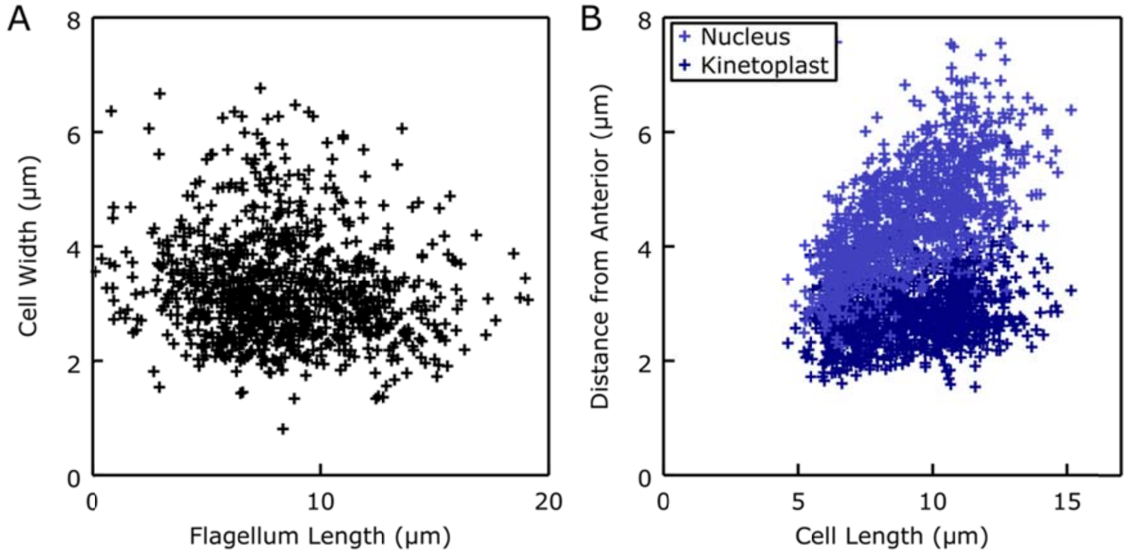

Figure S1. Further morphology analysis of promastigote *L. mexicana*. A. Scatter plot of cell width against flagellum length of cells from all three culture densities, n=980. Cell width and flagellum length does not show a correlation. **B.** Kinetoplast position (dark blue) does not vary according to cell length and is found at a constant distance of around 2.5 µm from the anterior end of the cell. Nuclear position (light blue) shows a positive correlation with cell length. Both kinetoplast and nucleus position show pooled data from all three culture densities, n=980.

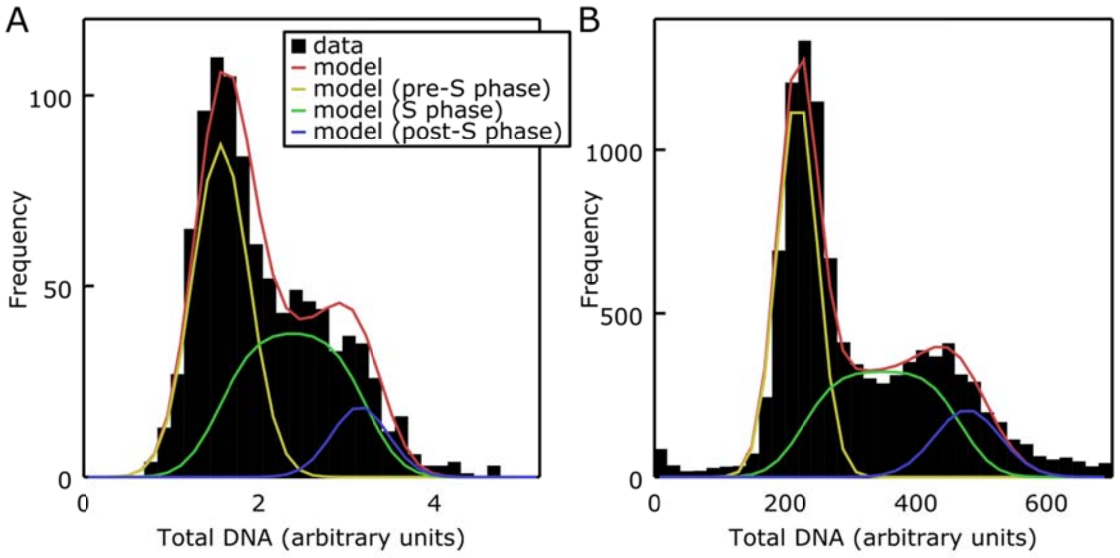

Figure S2. Calculation of S phase length and timing from histograms of cellular DNA. Timings of pre-S phase, S phase and post-S phase cell cycle stages can be calculated by fitting a curve (red) to a histogram of cellular DNA content. Black bars represent the experimental data. The four lines indicate components of the fitted model; the area under the red, yellow, green and blue lines gives the number of cells in total, during pre-S phase, during S phase and during post-S phase respectively. **A.** Model fitting to microscopy data (DNA content measured by DAPI staining), n=980. **B.** Model fitting to flow cytometry data (DNA content measured by propidium iodide staining), n=10000.

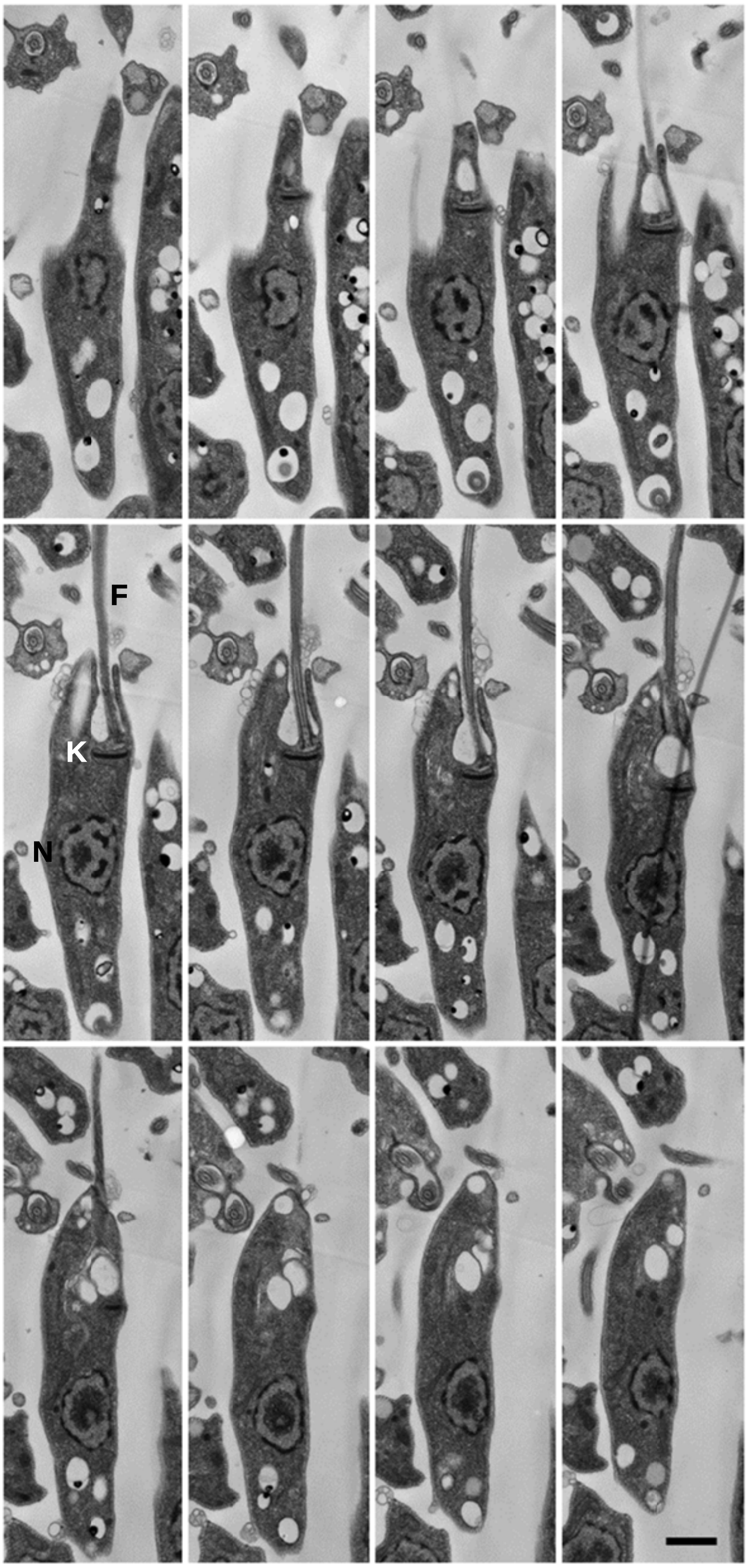

Figure S3. Electron microscopy of serial sections through a 1K1N cell. 12 adjacent 90 nm longitudinal sections through a 1K1N cell show the single basal body/pro-basal body pair. For higher magnification of the basal body region see Figure 8B, C. The nucleus (N), kinetoplast (K) and flagellum (F) are indicated. The scale bar represents 1 μm.

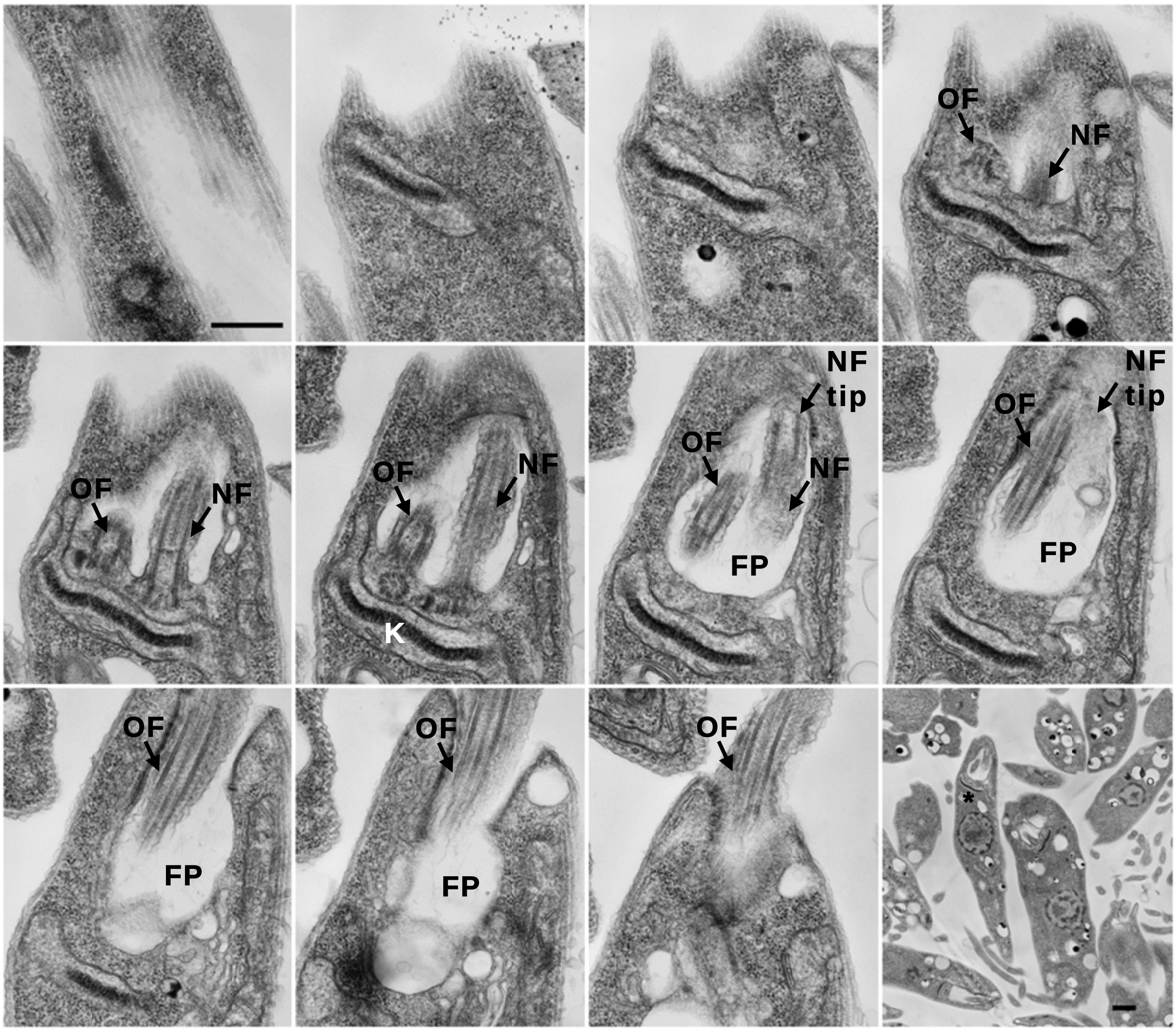

Figure S4. Electron microscopy of serial sections through a cell in the early stages of new flagellum growth. 11 adjacent 90 nm longitudinal sections through the cell shown in Figure 8D–F. The flagellar pocket (FP), old flagellum (OF, which extends from the flagellar pocket), new flagellum (NF, which does not extend from the flagellar pocket), new flagellum tip (NF tip) and kinetoplast (K) are indicated. The asterisk in the final panel indicates the region shown at higher magnification in the first 11 panels. The scale bar represents 500 nm (panels 1–11) or 1 µm (panel 12).

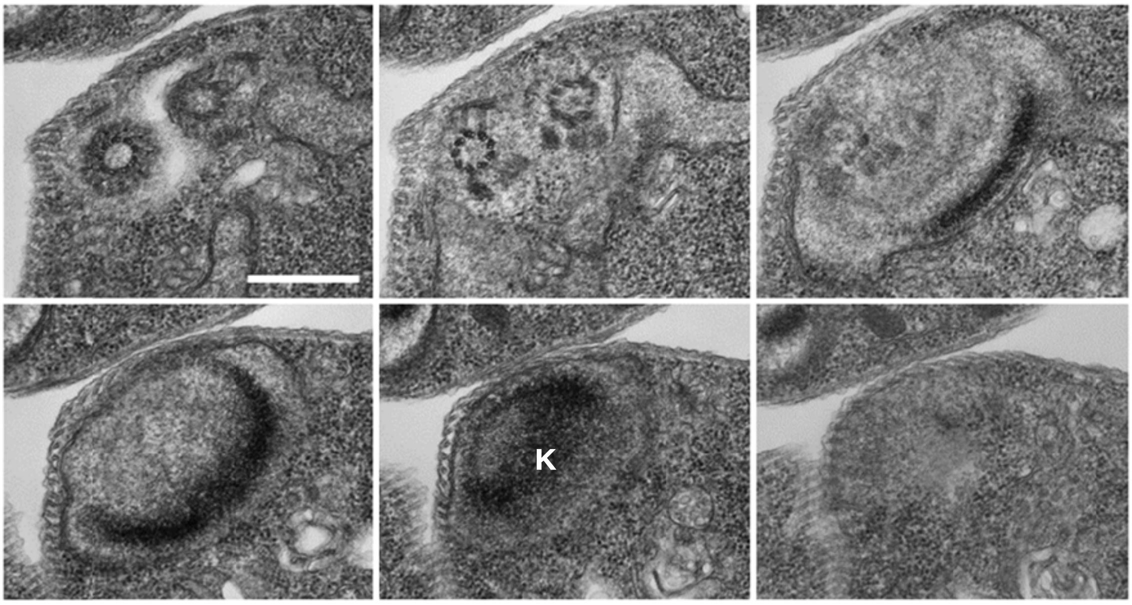

Figure S5. Electron microscopy of serial sections through a cell with a single kinetoplast and two basal bodies. 6 adjacent 90 nm sections show two basal body/pro-basal body pairs with the pro-basal bodies lying orthogonal to their partner basal body. For higher magnification of the basal bodies see Figure 8K. The kinetoplast (K) is indicated. The scale bar represents 500 nm.

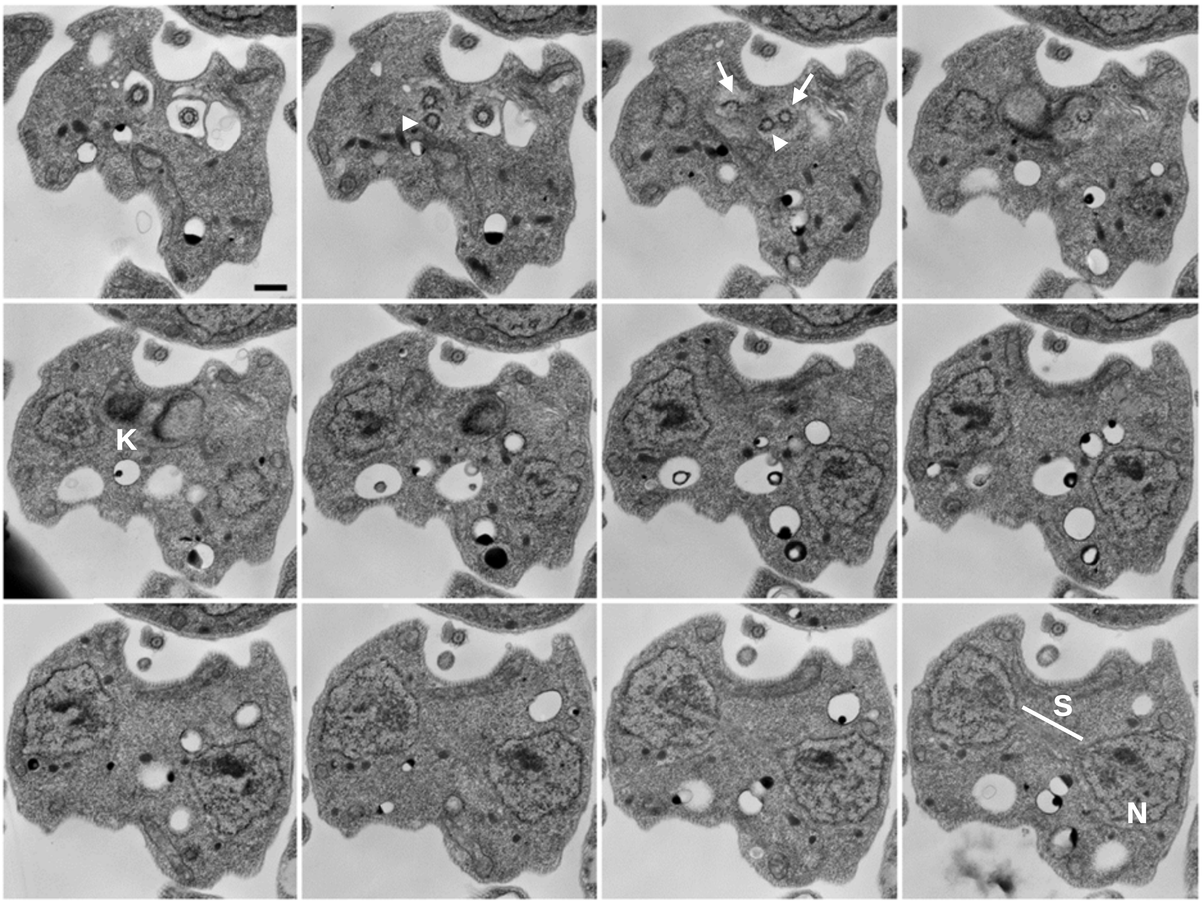

Figure S6. Electron microscopy of serial sections through a cell with a dividing kinetoplast and mitotic nucleus. 12 adjacent 90 nm longitudinal sections show the late mitotic nucleus (N) and spindle (S), the dividing kinetoplast (K) and the two basal body (arrows)/pro-basal body (arrowheads) pairs. The pro-basal bodies lie parallel to their partner basal body. The scale bar represents 500 nm.

Video S1. Time-lapse videos of *Leishmaniamexicana* progressing through the cell cycle. Cells were trapped in fluid pockets within 0.5% agarose to assist observation and a z-stack was captured every 1 min for 3.5 h. The most in focus slice was selected and rotated for every time point to give a constant cell orientation. Four cells observed at different stages of the cell cycle are shown. Based on morphology data from Figure 5 the four cells were aligned in time to the cell cycle to give a composite overview of the entire cell cycle. Video playback is at 10 frames per second, i.e. 600× actual speed. The scale bar represents 10 µm.

Table S1. Complete *L. mexicana* promastigote morphometric data set.

Culture Density (cells/ml)	Image No	Kinetoplast Count	Nucleus Count	Flagellum Count	Long Flagellum Length (µm)	Short Flagellum Length (µm)	Cell Body Length (µm)	Cell Body Width (µm)	Kin Anterior Distance 1 (µm)	Nuc Anterior Distance 1 (µm)	Kin Nuc Distance 1 (µm)	Kin Anterior Distance 2 (µm)	Nuc Anterior Distance 2 (µm)	Kin Nuc Distance 2 (µm)	Normalised Kin DNA 1 (arbitary)	Normalised Kin DNA 2 (arbitary)	Normalised Nuc DNA 1 (arbitary)	Normalised Nuc DNA 2 (arbitary)	Cell Cycle Progress (u)
3E+06	1	1	1	1	8.769	n/a	10.339	3.443	2.926	4.735	2.181	n/a	n/a	n/a	1.093	n/a	1.102	n/a	0.3459
3E+06	1	1	1	1	7.872	n/a	7.618	3.114	3.915	5.171	1.322	n/a	n/a	n/a	1.47	n/a	1.111	n/a	0.1031
3E+06	1	1	1	1	11.644	n/a	10.464	3.195	2.591	4.182	1.931	n/a	n/a	n/a	1.414	n/a	1.107	n/a	0.3749
3E+06	1	1	1	1	4.532	n/a	9.396	3.227	2.891	4.38	2.448	n/a	n/a	n/a	1.795	n/a	1.388	n/a	0.5133
3E+06	1	2	2	2	7.59	3.512	8.959	5.61	3.757	3.271	1.604	4.527	4.953	2.109	1.291	0.882	1.018	1.343	0.858
3E+06	1	1	1	1	11.949	n/a	12.115	5.223	3.66	5.331	2.535	n/a	n/a	n/a	2.372	n/a	1.695	n/a	0.6844
3E+06	1	1	2	5	10.984	10.509	9.032	5.883	2.891	4.424	1.674	n/a	4.123	2.085	2.083	n/a	1.032	1.245	0.8703
3E+06	1	1	1	1	8.18	n/a	8.334	4.909	2.948	4.768	1.252	n/a	n/a	n/a	1.063	n/a	1.224	n/a	0.1425
3E+06	1	1	1	1	5.309	n/a	9.077	3.825	3.085	5.164	2.233	n/a	n/a	n/a	0.937	n/a	1.305	n/a	0.2326
3E+06	1	1	1	1	5.5	n/a	8.305	5.012	2.996	4.979	3.757	n/a	n/a	n/a	1.55	n/a	2.148	n/a	1
3E+06	1	2	2	2	8.324	8	13.102	4.022	2.535	4.661	2.768	3.462	5.601	2.801	0.819	1.691	1.289	1.953	0.786
3E+06	1	1	1	1	4.272	n/a	9.397	4.315	2.948	5.338	2.891	n/a	n/a	n/a	0.993	n/a	1.356	n/a	0.2648
3E+06	1	1	1	1	0.975	n/a	7.53	2.721	3.183	4.877	1.735	n/a	n/a	n/a	0.926	n/a	0.9	n/a	0.0553
3E+06	1	1	2	2	7.553	2.228	9.078	5.096	2.801	4.653	2.151	n/a	4.182	1.958	2.118	n/a	1.282	1.621	0.8336
3E+06	1	1	1	1	9.425	n/a	13.54	4.412	4.362	6.598	3.882	n/a	n/a	n/a	1.741	n/a	2.208	n/a	0.6772
3E+06	1	1	2	2	5.015	0.717	8.196	5.518	n/a	4.888	2.996	n/a	5.035	1.793	2.906	n/a	1.05	1.388	0.8457
3E+06	1	1	1	1	5.46	n/a	12.953	n/a	3.983	6.386	3.371	n/a	n/a	n/a	1.915	n/a	1.89	n/a	0.617
3E+06	1	1	1	1	11.255	n/a	10.169	4.424	3.604	6.699	3.443	n/a	n/a	n/a	1.714	n/a	1.467	n/a	0.5036
3E+06	1	1	1	1	8.985	n/a	12.701	4.333	3.812	7.261	3.636	n/a	n/a	n/a	2.18	n/a	1.669	n/a	0.6274
3E+06	1	1	1	1	4.824	n/a	7.863	3.462	2.669	4.516	2.251	n/a	n/a	n/a	0.812	n/a	1.061	n/a	0.0934
3E+06	2	1	1	1	5.01	n/a	9.591	3.879	3.371	5.221	2.805	n/a	n/a	n/a	1.643	n/a	1.161	n/a	0.3373
3E+06	2	1	1	1	11.488	n/a	13.348	3.306	3.825	6.311	3.114	n/a	n/a	n/a	2.227	n/a	2.015	n/a	0.7175
3E+06	2	1	1	1	7.125	n/a	11.139	2.944	3.207	5.41	2.151	n/a	n/a	n/a	1.399	n/a	1.501	n/a	0.4594
3E+06	2	1	1	1	5.994	n/a	10.551	3.744	2.983	5.253	2.688	n/a	n/a	n/a	1.411	n/a	1.626	n/a	0.4814
3E+06	2	1	1	1	11.732	n/a	11.674	3.825	3.905	5.473	2.279	n/a	n/a	n/a	1.311	n/a	1.245	n/a	0.4196
3E+06	2	1	2	2	7.446	3.107	6.851	5.795	3.211	3.702	2.957	n/a	3.636	1.027	1.521	n/a	1.334	1.066	0.9955
3E+06	2	2	2	2	6.793	3.426	6.07	5.53	2.157	2.61	0.717	3.223	3.337	0.935	1.148	1.169	0.953	0.929	0.991
3E+06	2	1	1	1	9.145	n/a	12.028	4.009	3.664	5.853	2.405	n/a	n/a	n/a	1.811	n/a	1.915	n/a	0.6032
3E+06	2	1	1	1	8.526	n/a	9.812	3.55	3.043	4.835	2.233	n/a	n/a	n/a	1.103	n/a	1.304	n/a	0.3174
3E+06	2	1	1	1	11.851	n/a	9.305	4.493	3.223	4.835	1.727	n/a	n/a	n/a	1.397	n/a	1.488	n/a	0.3118
3E+06	2	1	1	1	7.656	n/a	11.827	3.97	3.207	5.331	2.721	n/a	n/a	n/a	2.121	n/a	2.296	n/a	0.7325
3E+06	2	1	1	1	6.438	n/a	8.371	4.009	2.891	5.104	2.801	n/a	n/a	n/a	1.308	n/a	1.047	n/a	0.1575
3E+06	2	1	1	1	4.382	n/a	8.63	3.454	2.891	5.131	2.489	n/a	n/a	n/a	1.723	n/a	1.374	n/a	0.2379
3E+06	2	1	1	1	8.033	n/a	12.231	4.009	3.757	5.757	2.891	n/a	n/a	n/a	2.152	n/a	1.745	n/a	0.6379
3E+06	2	1	1	1	8.277	n/a	9.013	3.398	3.085	4.824	1.931	n/a	n/a	n/a	1.022	n/a	1.148	n/a	0.2194
3E+06	2	1	1	1	6.223	n/a	9.452	3.754	3.306	4.595	2.091	n/a	n/a	n/a	1.415	n/a	0.973	n/a	0.273
3E+06	2	1	1	1	7.208	n/a	9.612	3.586	3.064	6.045	3.195	n/a	n/a	n/a	1.44	n/a	1.259	n/a	0.3259
3E+06	2	1	1	1	11.561	n/a	10.188	3.74	3.043	4.735	2.041	n/a	n/a	n/a	0.799	n/a	0.994	n/a	0.2923
3E+06	2	1	1	1	9.817	n/a	11.139	4.303	3.636	5.966	3.586	n/a	n/a	n/a	1.796	n/a	1.747	n/a	0.5659
3E+06	2	1	1	1	4.896	n/a	7.321	4.009	3.812	4.447	1.877	n/a	n/a	n/a	0.94	n/a	1.268	n/a	0.0506
3E+06	2	1	1	1	4.692	n/a	8.278	4.675	3.74	4.047	1.252	n/a	n/a	n/a	0.976	n/a	0.977	n/a	0.1177
3E+06	2	1	1	2	9.49	2.612	10.561	5.331	2.869	4.784	2.443	n/a	n/a	n/a	2.128	n/a	1.738	n/a	0.6135
3E+06	2	1	1	1	3.29	n/a	9.56	4.019	3.402	5.426	2.091	n/a	n/a	n/a	1.194	n/a	1.461	n/a	0.3146
3E+06	3	1	1	1	6.9	n/a	10.612	3.195	3.227	5.485	2.405	n/a	n/a	n/a	1.663	n/a	1.501	n/a	0.4972
3E+06	3	1	1	1	9.456	n/a	12.591	5.01	3.402	4.592	1.513	n/a	n/a	n/a	2.314	n/a	2.401	n/a	0.7438
3E+06	3	1	1	1	6.584	n/a	11.605	3.064	3.757	5.688	2.726	n/a	n/a	n/a	1.946	n/a	1.572	n/a	0.5625
3E+06	3	1	1	2	8.353	0.717	12.355	4.218	2.745	5.936	2.996	n/a	n/a	n/a	2.477	n/a	2.652	n/a	0.7744
3E+06	3	1	1	2	18.451	6.638	7.152	3.875	3.371	4.261	1.293	n/a	n/a	n/a	0.872	n/a	1.293	n/a	0.0413
3E+06	3	1	1	1	3.495	n/a	10.274	3.517	3.337	4.318	1.134	n/a	n/a	n/a	1.453	n/a	1.512	n/a	0.4378
3E+06	3	1	1	1	8.258	n/a	7.937	3.409	2.801	4.94	2.495	n/a	n/a	n/a	1.388	n/a	1.312	n/a	0.1301
3E+06	3	1	1	1	6.043	n/a	10.912	3.402	3.462	5.294	2.731	n/a	n/a	n/a	2.793	n/a	1.483	n/a	0.699
3E+06	3	1	1	1	8.652	n/a	5.901	4.768	2.926	3.825	1.293	2.489	4.047	1.283	1.706	n/a	0.985	n/a	0.0136
3E+06	3	1	1	1	6.301	n/a	10.507	3.931	3.195	5.096	1.513	n/a	n/a	n/a	1.371	n/a	1.555	n/a	0.4348
3E+06	3	1	1	1	7.271	n/a	12.176	3.657	3.862	5.473	3.163	n/a	n/a	n/a	1.377	n/a	1.728	n/a	0.4877
3E+06	3	1	1	1	14.291	n/a	11.013	4.768	3.944	5.585	2.028	n/a	n/a	n/a	2.469	n/a	1.488	n/a	0.645
3E+06	3	1	1	1	9.176	n/a	9.118	4.538	2.745	4.561	2.591	n/a	n/a	n/a	1.768	n/a	1.294	n/a	0.309
3E+06	3	1	1	1	4.558	n/a	8.725	3.532	3.183	4.435	2.384	n/a	n/a	n/a	1.302	n/a	1.468	n/a	0.222
3E+06	3	1	1	1	7.671	n/a	10.445	4.315	3.528	4.885	2.405	n/a	n/a	n/a	1.98	n/a	1.716	n/a	0.5895

3E+06	3	1	1	1	7.132	n/a	11.801	4.683	2.721	7.345	3.944	n/a	n/a	n/a	2.645	n/a	2.189	n/a	0.7628
3E+06	3	1	1	1	4.088	n/a	7.97	3.774	3.207	4.49	1.931	n/a	n/a	n/a	1.379	n/a	1.343	n/a	0.1375
3E+06	3	1	1	1	4.867	n/a	7.515	3.306	3.247	4.76	1.814	n/a	n/a	n/a	1.537	n/a	1.041	n/a	0.0862
3E+06	5	1	1	1	0.935	n/a	5.811	4.493	3.29	3.151	1.87	n/a	n/a	n/a	1.426	n/a	1.214	n/a	0.0114
3E+06	5	1	1	1	2.746	n/a	7.741	4.315	3.983	4.716	1.452	n/a	n/a	n/a	1.046	n/a	1.053	n/a	0.0886
3E+06	5	1	1	1	8.015	n/a	11.386	3.047	3.754	5.49	2.745	n/a	n/a	n/a	2.309	n/a	1.68	n/a	0.6592
3E+06	5	1	1	1	4.984	n/a	7.121	3.517	3.532	4.435	1.293	n/a	n/a	n/a	1.728	n/a	0.818	n/a	0.0483
3E+06	5	1	1	1	6.166	n/a	9.339	3.462	2.368	4.906	2.571	n/a	n/a	n/a	1.599	n/a	1.224	n/a	0.3062
3E+06	5	1	1	1	7.837	n/a	9.919	4.303	3.812	5.987	2.669	n/a	n/a	n/a	3.456	n/a	1.765	n/a	0.8057
3E+06	5	1	1	1	2.645	n/a	10.407	4.237	3.74	6.319	3.29	n/a	n/a	n/a	2.693	n/a	2.263	n/a	0.794
3E+06	5	1	1	1	6.745	n/a	10.124	4.617	3.757	6.106	2.61	n/a	n/a	n/a	1.619	n/a	1.568	n/a	0.5101
3E+06	5	1	1	1	8.012	n/a	12.538	3.875	3.306	6.897	3.462	4.022	7.549	3.664	2.911	n/a	2.193	n/a	0.7705
3E+06	5	1	1	1	3.918	n/a	7.041	4.458	3.114	3.183	0.717	n/a	n/a	n/a	1.004	n/a	1.069	n/a	0.0251
3E+06	5	1	1	1	9.555	n/a	10.787	3.08	4.197	7.487	3.517	n/a	n/a	n/a	2.165	n/a	1.769	n/a	0.6344
3E+06	5	1	1	1	8.489	n/a	10.976	4.318	3.774	6.402	2.726	n/a	n/a	n/a	3.227	n/a	2.068	n/a	0.7899
3E+06	5	1	1	1	2.891	n/a	9.734	3.409	3.29	5.674	2.855	n/a	n/a	n/a	1.933	n/a	1.271	n/a	0.5198
3E+06	5	1	1	1	8.194	n/a	10.488	4.088	3.757	6.127	2.731	n/a	n/a	n/a	1.923	n/a	1.839	n/a	0.5997
3E+06	5	2	2	2	7.249	2.381	7.618	5.966	3.561	4.592	1.221	3.163	4.661	1.735	0.684	1.846	0.951	1.108	0.9165
3E+06	7	1	1	1	6.572	n/a	9.302	3.114	2.63	4.403	1.793	n/a	n/a	n/a	1.083	n/a	1.365	n/a	0.2594
3E+06	7	1	1	1	3.086	n/a	8.665	3.636	3.744	4.237	1.612	n/a	n/a	n/a	1.431	n/a	1.335	n/a	0.2168
3E+06	7	1	1	1	8.602	n/a	8.636	4.595	3.383	4.318	2.51	n/a	n/a	n/a	1.214	n/a	1.518	n/a	0.2115
3E+06	7	1	1	1	8.439	n/a	8.037	3.628	2.926	4.592	1.705	n/a	n/a	n/a	0.946	n/a	1.234	n/a	0.1104
3E+06	7	1	1	1	4.119	n/a	5.615	4.022	2.745	3.586	1.604	n/a	n/a	n/a	0.984	n/a	1.312	n/a	0.0045
3E+06	7	1	1	1	3.469	n/a	8.75	3.517	4.163	5.61	2.268	n/a	n/a	n/a	0.915	n/a	1.125	n/a	0.175
3E+06	7	1	1	1	6.147	n/a	7.214	3.604	3.183	4.501	2.181	n/a	n/a	n/a	1.097	n/a	1.479	n/a	0.0577
3E+06	7	1	1	1	5.826	n/a	11.458	4.309	3.302	5.901	2.495	n/a	n/a	n/a	1.464	n/a	1.378	n/a	0.4563
3E+06	7	2	2	2	8.474	4.441	8.246	5.063	2.489	3.604	1.651	2.61	3.754	1.814	0.963	1.146	1.046	0.979	0.9865
3E+06	7	2	2	2	6.691	3.227	7.28	5.987	2.313	3.915	1.604	2.74	4.057	1.434	0.875	1.046	1.059	1.54	0.9208
3E+06	7	1	1	1	12.989	n/a	11.795	4.012	3.74	5.64	2.411	n/a	n/a	n/a	1.198	n/a	1.293	n/a	0.4165
3E+06	7	1	1	1	1.513	n/a	9.227	3.902	3.618	5.012	2.411	n/a	n/a	n/a	1.401	n/a	1.34	n/a	0.2867
3E+06	7	1	1	1	5.536	n/a	9.576	3.862	2.842	5.134	2.654	n/a	n/a	n/a	0.887	n/a	1.402	n/a	0.2785
3E+06	8	1	1	1	3.741	n/a	9.342	3.586	2.948	5.071	2.028	n/a	n/a	n/a	2.024	n/a	2.07	n/a	0.6556
3E+06	8	1	1	1	4.661	n/a	8.363	4.27	3.432	5.294	2.505	n/a	n/a	n/a	1.37	n/a	1.406	n/a	0.183
3E+06	8	1	1	1	11.797	n/a	10.066	3.207	2.448	4.628	1.931	n/a	n/a	n/a	1.06	n/a	1.241	n/a	0.3344
3E+06	8	1	1	1	13.356	n/a	13.41	2.745	2.645	5.455	2.566	n/a	n/a	n/a	1.54	n/a	1.465	n/a	0.4751
3E+06	8	1	1	1	11.877	n/a	7.962	5.688	3.931	5.253	2.181	n/a	n/a	n/a	1.396	n/a	1.88	n/a	0.1779
3E+06	8	1	1	2	8.097	1.897	11.636	3.586	3.825	6.272	2.51	n/a	n/a	n/a	2.54	n/a	2.196	n/a	0.759
3E+06	8	2	2	2	9.957	2.827	9.301	4.653	2.688	4.675	1.076	3.306	3.744	2.296	1.64	1.122	1.08	1.271	0.8216
3E+06	8	1	1	1	10.324	n/a	7.864	3.306	3.211	4.749	1.221	n/a	n/a	n/a	1.645	n/a	1.449	n/a	0.15
3E+06	8	1	1	1	4	n/a	8.402	3.502	2.801	5.02	n/a	n/a	n/a	n/a	0.933	n/a	0.889	n/a	0.1227
3E+06	8	1	1	1	3.247	n/a	8.613	3.085	3.383	4.848	1.604	n/a	n/a	n/a	1.369	n/a	1.235	n/a	0.2037
3E+06	8	1	1	1	7.959	n/a	11.551	3.398	3.702	6.947	4.261	n/a	n/a	n/a	1.302	n/a	1.604	n/a	0.4688
3E+06	8	1	1	1	7.076	n/a	7.166	3.469	2.453	3.432	1.479	n/a	n/a	n/a	0.686	n/a	1.209	n/a	0.0343
3E+06	8	1	1	1	10.862	n/a	10.325	3.371	3.306	4.377	2.296	n/a	n/a	n/a	1.567	n/a	1.844	n/a	0.5492
3E+06	8	1	1	1	9.186	n/a	9.494	4.716	3.744	5.742	1.828	n/a	n/a	n/a	1.367	n/a	1.479	n/a	0.3316
3E+06	8	1	1	1	7.952	n/a	12.767	2.891	5.811	3.227	n/a	n/a	n/a	n/a	1.811	n/a	1.639	n/a	0.5426
3E+06	8	1	1	1	6.346	n/a	10.8	4.022	3.428	5.782	3.051	n/a	n/a	n/a	1.669	n/a	1.659	n/a	0.5328
3E+06	8	1	1	1	7.035	n/a	7.861	3.398	2.891	4.182	1.771	n/a	n/a	n/a	1.395	n/a	1.222	n/a	0.1202
3E+06	8	1	1	0	n/a	n/a	10.255	4.038	3.306	5.455	2.245	n/a	n/a	n/a	1.204	n/a	1.57	n/a	0.3926
3E+06	8	1	1	1	3.114	n/a	9.88	3.882	3.73	4.95	2.028	n/a	n/a	n/a	1.512	n/a	1.592	n/a	0.4845
3E+06	8	1	1	1	12.96	n/a	9.074	4.315	3.383	5.585	2.181	n/a	n/a	n/a	1.019	n/a	1.217	n/a	0.2299
3E+06	8	1	1	1	8.298	n/a	11.327	3.064	3.243	4.811	1.951	n/a	n/a	n/a	0.97	n/a	1.369	n/a	0.3985
3E+06	8	1	1	0	n/a	n/a	6.683	4.76	3.097	5.033	1.156	n/a	n/a	n/a	1.137	n/a	1.161	n/a	0.0182
3E+06	8	1	1	1	7.108	n/a	8.114	4.098	2.505	3.586	1.674	n/a	n/a	n/a	0.942	n/a	1.388	n/a	0.1251
3E+06	8	1	1	1	3.153	n/a	9.003	3.692	3.902	5.452	2.228	n/a	n/a	n/a	0.996	n/a	1.366	n/a	0.2273
3E+06	9	1	1	1	7.401	n/a	9.794	3.744	3.409	4.683	1.828	n/a	n/a	n/a	1.004	n/a	1.061	n/a	0.2758
3E+06	9	1	1	1	11.129	n/a	11.867	4.057	3.051	5.951	3.163	n/a	n/a	n/a	1.175	n/a	1.577	n/a	0.444
3E+06	9	1	1	1	13.284	n/a	10.328	3.383	2.745	5.594	2.983	n/a	n/a	n/a	1.225	n/a	1.154	n/a	0.3545
3E+06	9	1	1	1	6.389	n/a	7.516	3.432	2.63	4.27	1.434	n/a	n/a	n/a	1.159	n/a	1.397	n/a	0.0838
3E+06	9	1	1	1	6.982	n/a	10.82	3.402	3.961	5.154	2.028	n/a	n/a	n/a	1.06	n/a	1.347	n/a	0.3808
3E+06	9	1	1	1	7.468	n/a	10.801	3.517	3.586	5.071	2.151	n/a	n/a	n/a	1.625	n/a	1.743	n/a	0.5393
3E+06	9	1	1	1	9.96	n/a	11.56	3.875	4.012	6.44	3.183	n/a	n/a	n/a	1.65	n/a	1.599	n/a	0.5262
3E+06	9	1	1	1	13.696	n/a	10.354	2.443	2.335	4.974	2.996	n/a	n/a	n/a	1.349	n/a	1.242	n/a	0.3778
3E+06	9	1	1	1	3.806	n/a	8.402	3.302	3.517	4.019	1.479	n/a	n/a	n/a	1.058	n/a	1.535	n/a	0.1728
3E+06	9	1	1	1	4.589	n/a	8.982	3.454	3.371	4.342	2.411	n/a	n/a	n/a	1.317	n/a	1.195	n/a	0.2433

3E+06	9	1	1	1	11.722	n/a	10.382	3.183	4.403	6.604	2.837	n/a	n/a	n/a	1.631	n/a	1.412	n/a	0.4657
3E+06	9	1	1	2	6.085	1.579	11.19	5.094	3.247	4.848	1.951	n/a	n/a	n/a	1.998	n/a	2.084	n/a	0.67
3E+06	9	1	1	1	5.142	n/a	10.841	3.961	3.852	6.036	2.654	n/a	n/a	n/a	2.132	n/a	2.416	n/a	0.7362
3E+06	9	1	1	1	7.045	n/a	8.833	3.862	3.151	4.974	0.975	n/a	n/a	n/a	0.788	n/a	1.197	n/a	0.1856
3E+06	9	2	2	2	5.719	1.931	6.45	6.246	3.348	4.435	0.975	2.869	4.558	1.361	1.116	1.257	0.93	1.267	0.9337
3E+06	9	2	2	2	2.983	1.167	8.246	6.67	4.138	5.518	1.828	3.207	4.342	1.793	1.193	1.128	1.13	1.374	0.8786
3E+06	9	1	1	1	5.737	n/a	8.613	4.182	3.207	4.94	1.604	n/a	n/a	n/a	1.763	n/a	1.775	n/a	0.5793
3E+06	9	1	1	1	9.202	n/a	7.955	2.645	3.211	4.009	0.818	n/a	n/a	n/a	1.297	n/a	0.794	n/a	0.1055
3E+06	9	1	1	0	n/a	n/a	11.263	4.303	4.675	6.886	2.996	n/a	n/a	n/a	2.479	n/a	2.187	n/a	0.7552
3E+06	9	1	1	1	8.606	n/a	12.251	3.692	4.082	5.793	2.654	n/a	n/a	n/a	1.863	n/a	2.166	n/a	0.6736
3E+06	9	1	1	1	7.05	n/a	9.467	2.731	2.768	4.695	2.405	n/a	n/a	n/a	0.975	n/a	1.23	n/a	0.2621
3E+06	9	1	1	1	11.552	n/a	9.989	4.088	3.657	5.455	2.405	n/a	n/a	n/a	1.183	n/a	1.474	n/a	0.3516
3E+06	10	1	1	1	7.207	n/a	9.309	3.586	4.592	6.543	2.085	n/a	n/a	n/a	1.245	n/a	1.338	n/a	0.2812
3E+06	10	1	1	1	4.455	n/a	8.75	2.837	3.085	4.098	1.604	n/a	n/a	n/a	1.089	n/a	1.164	n/a	0.1985
3E+06	10	1	1	1	4.182	n/a	8.147	2.51	n/a	3.432	4.237	n/a	n/a	n/a	1.135	n/a	1.214	n/a	0.1276
3E+06	10	1	1	2	4.456	3.961	10.462	3.72	4.303	6.551	2.489	n/a	n/a	n/a	1.206	n/a	1.105	n/a	0.3632
3E+06	10	1	1	1	4.031	n/a	11.431	4.309	4.138	6.402	3.207	n/a	n/a	n/a	1.368	n/a	1.453	n/a	0.447
3E+06	10	2	2	2	0.864	0.661	8.523	6.366	3.348	4.561	1.479	3.114	4.661	1.37	1.656	0.957	1.088	1.242	0.8539
3E+06	10	1	1	1	9.694	n/a	13.791	3.368	3.542	6.416	3.368	n/a	n/a	n/a	2.112	n/a	1.969	n/a	0.7027
3E+06	10	1	1	1	12.337	n/a	12.589	3.302	3.064	5.071	2.801	n/a	n/a	n/a	1.547	n/a	1.661	n/a	0.5165
3E+06	10	1	1	1	12.421	n/a	9.791	2.855	2.427	4.877	2.448	n/a	n/a	n/a	0.598	n/a	0.718	n/a	0.2567
3E+06	10	2	2	2	2.948	1.87	8.142	5.61	3.08	4.234	1.604	2.904	3.383	1.379	1.274	1.149	1.265	1.72	0.8417
3E+06	10	1	2	2	9.484	2.61	7.605	6.35	2.51	4.218	3.223	n/a	4.885	1.87	2.063	n/a	1.1	1.017	0.982
3E+06	10	1	1	1	8.705	n/a	11.294	3.967	4.009	5.331	3.243	n/a	n/a	n/a	0.852	n/a	1.249	n/a	0.369
3E+06	10	1	1	0	n/a	n/a	8.82	4.022	4.279	4.974	1.513	n/a	n/a	n/a	1.394	n/a	1.041	n/a	0.2141
3E+06	10	1	1	1	3.722	n/a	7.858	3.702	3.967	4.822	1.479	n/a	n/a	n/a	1.009	n/a	1.043	n/a	0.0982
3E+06	10	1	1	1	2.261	n/a	8.878	3.195	3.151	4.561	2.085	n/a	n/a	n/a	1.072	n/a	1.207	n/a	0.2063
3E+06	10	1	1	1	13.388	n/a	8.419	2.028	2.535	4.738	2.054	n/a	n/a	n/a	0.776	n/a	0.769	n/a	0.108
3E+06	10	1	1	1	6.678	n/a	9.467	3.862	3.443	5.71	2.313	n/a	n/a	n/a	1.952	n/a	1.954	n/a	0.6204
3E+06	10	1	1	0	n/a	n/a	10.929	4.123	3.409	5.695	2.427	n/a	n/a	n/a	1.699	n/a	2.046	n/a	0.5963
3E+06	10	1	1	1	9.285	n/a	9.655	4.009	3.74	5.023	1.771	n/a	n/a	n/a	1.141	n/a	1.483	n/a	0.3231
3E+06	10	2	2	2	6.231	3.611	6.543	6.354	3.348	4.009	1.37	2.669	3.66	2.085	0.842	1.077	0.986	1.18	0.9776
3E+06	10	1	1	1	10.381	n/a	11.775	3.915	3.443	5.987	3.085	n/a	n/a	n/a	1.732	n/a	1.985	n/a	0.5929
3E+06	10	1	1	1	5.352	n/a	7.189	3.66	3.825	4.377	1.156	n/a	n/a	n/a	0.708	n/a	1.149	n/a	0.032
3E+06	10	1	2	2	7.618	3.743	8.203	4.971	2.886	3.961	1.931	n/a	3.905	1.134	1.282	n/a	0.886	1.032	0.1933
3E+06	11	1	1	1	14.901	n/a	11.884	4.038	3.604	5.795	3.047	n/a	n/a	n/a	1.427	n/a	1.753	n/a	0.5004
3E+06	11	1	1	1	5.221	n/a	10.82	4.716	3.469	4.877	1.579	n/a	n/a	n/a	1.207	n/a	1.072	n/a	0.3661
3E+06	11	1	1	1	6.494	n/a	12.213	3.097	3.586	5.793	2.957	n/a	n/a	n/a	1.704	n/a	1.562	n/a	0.5295
3E+06	11	1	1	1	6.913	n/a	7.475	3.628	3.337	4.088	1.322	n/a	n/a	n/a	0.73	n/a	0.956	n/a	0.0459
3E+06	11	1	1	1	6.907	n/a	10.627	3.542	2.586	4.218	2.505	n/a	n/a	n/a	1.181	n/a	1.489	n/a	0.4015
3E+06	11	1	1	2	4.719	0.935	9.596	5.134	3.371	4.784	2.759	n/a	n/a	n/a	2.487	n/a	2.431	n/a	0.8176
3E+06	11	2	2	2	8.9	3.163	7.95	6.472	2.61	4.038	1.452	2.61	4.67	1.322	1.455	1.366	1.42	1.245	0.8498
3E+06	11	1	1	1	4.304	n/a	7.549	3.586	4.169	5.154	1.958	n/a	n/a	n/a	1.369	n/a	1.023	n/a	0.0814
3E+06	11	1	1	1	9.973	n/a	12.628	3.247	2.759	6.326	2.654	n/a	n/a	n/a	1.226	n/a	1.63	n/a	0.4719
3E+06	11	1	1	1	7.39	n/a	10.682	3.561	2.61	4.088	1.958	n/a	n/a	n/a	1.145	n/a	1.42	n/a	0.3867
3E+06	11	2	2	2	8.169	2.759	7.475	6.272	2.591	3.862	1.983	2.63	3.469	1.322	1.181	1.113	1.437	1.207	0.8953
3E+06	11	1	1	1	9.051	n/a	6.803	4.625	3.227	4.303	1.361	n/a	n/a	n/a	1.57	n/a	0.899	n/a	0.0228
3E+06	11	1	1	1	4.634	n/a	7.665	3.337	2.842	3.754	1.134	n/a	n/a	n/a	1.383	n/a	0.964	n/a	0.0958
3E+06	11	1	1	1	7.089	n/a	11.083	4.182	3.502	6.464	3.163	n/a	n/a	n/a	1.195	n/a	1.327	n/a	0.4075
3E+06	11	1	1	2	5.444	2.085	8.174	3.852	3.159	4.735	2.028	n/a	n/a	n/a	2.82	n/a	1.533	n/a	0.9251
3E+06	11	1	1	1	10.693	n/a	7.863	3.097	3.306	4.885	1.931	n/a	n/a	n/a	0.874	n/a	0.992	n/a	0.091
3E+06	11	1	1	1	6.466	n/a	9.75	2.61	2.759	4.762	2.313	n/a	n/a	n/a	0.899	n/a	1.29	n/a	0.284
3E+06	11	2	2	2	9.038	3.148	8.326	5.253	2.996	3.657	1.014	2.591	4.182	1.877	1.56	1.228	0.919	0.78	0.9038
3E+06	11	1	1	1	4.261	n/a	8.145	3.443	3.302	4.814	1.958	n/a	n/a	n/a	1.346	n/a	1.34	n/a	0.155
3E+06	11	1	1	1	10.883	n/a	12.204	3.211	4.012	4.564	1.651	n/a	n/a	n/a	0.902	n/a	1.315	n/a	0.4105
3E+06	11	2	2	2	5.996	3.744	7.603	5.033	3.195	5.455	2.645	2.726	4.541	1.513	0.653	0.858	1.196	1.217	0.9731
3E+06	11	1	1	1	4.574	n/a	9.342	3.915	3.862	5.613	2.948	n/a	n/a	n/a	1.16	n/a	1.839	n/a	0.3287
3E+06	11	1	1	1	7.255	n/a	8.926	3.223	2.904	4.919	2.405	n/a	n/a	n/a	1.17	n/a	1.363	n/a	0.2353
3E+06	11	1	1	1	10.978	n/a	10.127	3.085	3.047	5.809	2.443	n/a	n/a	n/a	0.902	n/a	1.206	n/a	0.3202
3E+06	11	1	1	1	6.211	n/a	7.159	3.211	3.636	5.164	2.443	n/a	n/a	n/a	1.809	n/a	1.295	n/a	0.0719
3E+06	12	0	2	1	12.995	n/a	10.82	2.586	3.55	6.127	2.405	n/a	n/a	n/a	n/a	n/a	1.19	0.725	0.3603
3E+06	12	1	1	1	4.304	n/a	8.821	4.234	3.247	4.493	2.181	n/a	n/a	n/a	1.128	n/a	1.643	n/a	0.2406
3E+06	12	1	1	1	10.209	n/a	7.508	3.398	4.047	3.604	0.507	n/a	n/a	n/a	0.693	n/a	0.917	n/a	0.0436
3E+06	12	2	2	2	8.347	4.764	6.598	5.782	3.175	4.318	0.453	3.398	3.983	1.764	0.526	0.769	1.413	1.069	0.9687
3E+06	12	1	1	1	6.274	n/a	11.097	3.657	3.043	6.851	4.197	n/a	n/a	n/a	1.421	n/a	1.59	n/a	0.4782

3E+06	12	1	1	1	3.306	n/a	11.295	4.107	3.337	6.386	2.957	n/a	n/a	n/a	1.933	n/a	2.456	n/a	0.725
3E+06	12	1	1	1	12.37	n/a	7.81	3.961	2.411	4.009	2.085	n/a	n/a	n/a	1.629	n/a	1.459	n/a	0.14
3E+06	12	1	1	1	7.871	n/a	10.447	3.043	2.296	5.311	2.443	n/a	n/a	n/a	1.296	n/a	1.653	n/a	0.4409
3E+06	12	1	1	1	14.458	n/a	10.904	3.849	3.561	5.124	3.825	n/a	n/a	n/a	1.067	n/a	1.662	n/a	0.4256
3E+06	12	1	1	1	3.852	n/a	10.454	4.333	3.879	6.328	3.159	n/a	n/a	n/a	2.346	n/a	1.775	n/a	0.6628
3E+06	12	1	1	1	9.738	n/a	8.34	3.561	3.013	5.035	2.313	n/a	n/a	n/a	1.459	n/a	1.437	n/a	0.1881
3E+06	12	1	1	1	12.989	n/a	12.188	3.961	3.657	6.188	2.837	n/a	n/a	n/a	2.258	n/a	2.115	n/a	0.7287
3E+06	12	1	1	1	4.592	n/a	10.704	3.528	3.702	7.121	4.057	n/a	n/a	n/a	2.812	n/a	2.138	n/a	0.7821
3E+06	12	2	2	2	7.169	2.801	8.185	5.292	2.801	5.782	2.891	3.502	5.02	1.793	1.113	1.557	0.982	1.516	0.862
3E+06	12	1	1	1	3.506	n/a	7.188	3.74	3.961	3.862	1.293	n/a	n/a	n/a	1.515	n/a	1.281	n/a	0.0671
3E+06	12	1	1	1	5.007	n/a	8.68	3.604	3.247	5.236	1.793	n/a	n/a	n/a	0.852	n/a	1.505	n/a	0.1907
3E+06	12	1	1	1	11.262	n/a	8.255	3.383	3.306	4.661	2.957	n/a	n/a	n/a	1.14	n/a	1.673	n/a	0.1702
3E+06	12	1	1	1	11.631	n/a	10.722	3.561	3.744	6.161	2.855	n/a	n/a	n/a	1.955	n/a	1.383	n/a	0.5361
3E+06	12	1	1	1	8.448	n/a	6.146	3.532	3.657	4.877	1.202	n/a	n/a	n/a	0.499	n/a	0.645	n/a	0.0068
3E+06	13	1	1	1	5.466	n/a	7.71	3.862	3.163	4.595	1.604	n/a	n/a	n/a	1.447	n/a	1.392	n/a	0.1153
3E+06	13	1	1	1	6.275	n/a	11.259	3.802	3.195	6.24	3.163	n/a	n/a	n/a	2.281	n/a	1.999	n/a	0.7064
3E+06	13	2	2	2	7.484	3.913	6.432	3.688	4.009	7.569	3.702	3.702	4.822	1.076	2.163	1.143	2.638	1.194	0.8376
3E+06	13	1	1	1	4.266	n/a	8.683	3.085	3.271	4.994	1.604	n/a	n/a	n/a	1.531	n/a	1.69	n/a	0.2513
3E+06	13	1	1	1	5.674	n/a	12.133	3.862	4.315	6.612	3.944	n/a	n/a	n/a	1.992	n/a	1.809	n/a	0.6101
3E+06	13	1	1	1	9.791	n/a	12.814	4.458	3.657	6.604	3.195	n/a	n/a	n/a	1.961	n/a	2.249	n/a	0.7138
3E+06	13	1	1	1	6.555	n/a	11.975	3.944	3.371	6.851	3.043	n/a	n/a	n/a	1.944	n/a	1.971	n/a	0.6415
3E+06	13	1	1	1	7.791	n/a	7.863	3.862	4.16	5.035	1.877	n/a	n/a	n/a	1.107	n/a	1.747	n/a	0.1325
3E+06	13	1	2	1	2.51	n/a	6.366	6.062	3.542	3.348	1.604	n/a	3.73	4.197	2.305	n/a	1.44	1.297	0.9123
3E+06	13	1	1	1	5.56	n/a	11.202	4.11	4.318	6.246	1.793	n/a	n/a	n/a	2.155	n/a	2.06	n/a	0.6954
3E+06	13	1	1	1	10.339	n/a	8.115	3.961	3.636	5.094	1.951	n/a	n/a	n/a	1.118	n/a	1.596	n/a	0.1525
3E+06	13	1	1	1	8.395	n/a	10.872	3.085	3.043	4.675	2.054	n/a	n/a	n/a	1.264	n/a	1.612	n/a	0.4317
3E+06	13	1	1	1	9.113	n/a	8.613	3.043	2.948	5.164	2.085	n/a	n/a	n/a	1.038	n/a	1.18	n/a	0.1677
3E+06	13	1	1	1	10.074	n/a	7.441	3.915	3.409	4.974	1.828	n/a	n/a	n/a	1.251	n/a	1.423	n/a	0.079
3E+06	13	1	1	1	4.648	n/a	9.163	2.535	2.983	3.604	1.521	n/a	n/a	n/a	0.675	n/a	1.112	n/a	0.2089
3E+06	13	1	1	1	6.35	n/a	10.774	3.812	3.227	4.979	1.635	n/a	n/a	n/a	0.856	n/a	1.807	n/a	0.4045
3E+06	13	1	1	1	4.754	n/a	6.884	3.026	3.528	3.862	1.479	n/a	n/a	n/a	0.844	n/a	1.354	n/a	0.0205
3E+06	13	1	1	1	10.348	n/a	10.274	3.428	3.243	5.782	2.926	n/a	n/a	n/a	1.481	n/a	1.526	n/a	0.4532
3E+06	13	1	1	1	6.299	n/a	11.106	4.038	3.66	6.67	3.528	n/a	n/a	n/a	1.713	n/a	1.47	n/a	0.5068
3E+06	13	1	1	1	14.592	n/a	9.741	2.891	2.505	5.035	1.958	n/a	n/a	n/a	1.165	n/a	1.13	n/a	0.295
3E+06	13	1	1	1	6.652	n/a	11.089	3.114	3.432	5.181	1.931	n/a	n/a	n/a	1.837	n/a	1.609	n/a	0.5525
3E+06	13	1	1	1	9.408	n/a	8.549	2.74	2.944	4.234	1.521	2.891	3.983	1.727	1.047	n/a	0.967	n/a	0.1475
3E+06	13	1	1	1	8.385	n/a	7.168	2.453	2.926	4.172	1.612	n/a	n/a	n/a	0.966	n/a	1.037	n/a	0.039
3E+06	14	1	1	1	3.365	n/a	7.377	3.849	1.924	4.012	1.612	n/a	n/a	n/a	1.412	n/a	1.248	n/a	0.0766
3E+06	14	1	1	1	4.914	n/a	7.252	4.403	2.926	4.209	2.268	n/a	n/a	n/a	1.185	n/a	1.423	n/a	0.0624
3E+06	14	1	1	1	9.911	n/a	9.219	4.209	3.961	5.613	2.505	n/a	n/a	n/a	1.828	n/a	2.203	n/a	0.6521
3E+06	14	1	1	1	7.692	n/a	5.221	6.227	2.157	2.489	1.931	n/a	n/a	n/a	1.198	n/a	1.763	n/a	0.0023
3E+06	14	1	1	1	9.077	n/a	9.787	3.454	3.227	6.106	2.591	n/a	n/a	n/a	2.163	n/a	1.355	n/a	0.5726
3E+06	14	1	1	1	6.64	n/a	10.614	3.223	3.618	4.853	2.313	n/a	n/a	n/a	1.544	n/a	1.85	n/a	0.5459
3E+06	14	2	2	2	7.563	2.983	7.089	5.811	2.891	4.456	1.156	3.306	5.096	0.802	1.304	1.005	1.163	1.359	0.908
3E+06	14	1	1	1	0.802	n/a	10.633	3.702	3.306	5.669	2.245	n/a	n/a	n/a	1.157	n/a	1.264	n/a	0.372
3E+06	14	1	1	1	1.705	n/a	10.886	4.683	3.402	5.966	2.726	n/a	n/a	n/a	1.137	n/a	1.34	n/a	0.3896
3E+06	14	1	1	1	6.195	n/a	9.787	3.371	2.731	5.379	2.109	n/a	n/a	n/a	2.819	n/a	1.728	n/a	0.7978
3E+06	14	1	1	0	n/a	n/a	9.866	4.749	3.223	6.094	2.731	n/a	n/a	n/a	2.583	n/a	2.08	n/a	0.8017
3E+06	14	1	1	0	n/a	n/a	9.692	3.882	3.882	5.485	2.313	n/a	n/a	n/a	1.182	n/a	1.656	n/a	0.343
3E+06	14	1	1	2	5.208	1.612	10.097	3.159	2.801	5.134	2.891	n/a	n/a	n/a	1.797	n/a	2.205	n/a	0.6485
3E+06	14	1	1	1	4.16	n/a	6.011	3.183	2.535	3.114	0.935	n/a	n/a	n/a	0.689	n/a	1.328	n/a	0.0091
3E+06	14	1	1	1	5.919	n/a	9.754	3.026	3.586	5.613	2.759	n/a	n/a	n/a	0.818	n/a	0.93	n/a	0.2703
3E+06	14	1	1	1	12.774	n/a	10.002	3.402	3.097	4.098	2.405	n/a	n/a	n/a	0.779	n/a	1.337	n/a	0.3006
3E+06	14	1	1	1	3.586	n/a	9.571	3.383	2.586	5.331	2.51	n/a	n/a	n/a	1.028	n/a	1.139	n/a	0.2676
3E+06	14	1	1	1	7.605	n/a	9.438	3.151	2.801	5.601	1.958	n/a	n/a	n/a	1.509	n/a	1.132	n/a	0.2978
3E+06	14	1	1	1	7.249	n/a	8.297	4.234	2.61	4.315	1.379	n/a	n/a	n/a	1.233	n/a	1.369	n/a	0.1651
3E+06	14	2	2	2	5.265	2.296	6.957	5.61	2.61	3.915	0.818	3.744	4.661	0.802	1.098	1.228	1.213	0.987	0.9294
3E+06	14	1	2	2	9.676	2.775	7.864	6.272	3.013	4.501	2.586	n/a	4.661	2.384	1.988	n/a	1.109	1.226	0.9424
3E+06	14	2	2	2	5.196	2.232	7.706	5.601	3.227	4.805	1.479	3.151	5.141	1.951	0.875	1.946	1.283	1.019	0.887
3E+06	15	1	1	1	8.103	n/a	12.09	3.306	3.163	5.737	3.175	n/a	n/a	n/a	1.218	n/a	1.37	n/a	0.4287
3E+06	15	1	1	1	6.932	n/a	9.77	3.454	3.207	5.094	4.022	n/a	n/a	n/a	0.978	n/a	1.233	n/a	0.2895
3E+06	15	1	1	1	9.898	n/a	9.947	3.306	3.114	5.445	3.688	n/a	n/a	n/a	0.936	n/a	1.569	n/a	0.3401
3E+06	15	2	2	2	7.758	1.156	6.592	5.34	3.852	4.824	1.156	3.29	3.586	1.322	0.869	0.97	0.764	1.083	0.9643
3E+06	15	1	1	1	6.532	n/a	10.271	2.891	2.842	5.578	2.586	n/a	n/a	n/a	1.293	n/a	1.16	n/a	0.3574
3E+06	15	2	2	2	7.381	3.552	7.921	6.766	3.74	4.261	1.322	3.223	3.812	0.864	1.285	1.428	1.24	1.178	0.8745

3E+06	15	1	1	1	5.394	n/a	10.97	4.138	3.195	5.309	2.759	n/a	n/a	n/a	2.276	n/a	2.123	n/a	0.7212
3E+06	15	1	1	1	8.457	n/a	12.681	4.16	3.73	5.896	2.805	n/a	n/a	n/a	1.861	n/a	1.915	n/a	0.6066
3E+06	15	1	1	1	3.331	n/a	7.539	3.026	2.869	4.303	1.705	n/a	n/a	n/a	1.021	n/a	1.214	n/a	0.0695
3E+06	15	1	1	1	9.911	n/a	10.662	2.768	2.886	4.94	2.151	n/a	n/a	n/a	1.073	n/a	1.519	n/a	0.3956
3E+06	15	1	1	0	n/a	n/a	7.123	4.94	3.306	4.088	1.076	n/a	n/a	n/a	0.938	n/a	1.169	n/a	0.0366
3E+06	15	1	1	1	12.492	n/a	10.863	3.409	2.869	6.612	3.247	n/a	n/a	n/a	1.834	n/a	1.732	n/a	0.5759
3E+06	15	1	1	1	3.064	n/a	7.598	2.886	2.996	4.835	1.604	n/a	n/a	n/a	1.108	n/a	1.521	n/a	0.1007
3E+06	15	1	1	1	7.496	n/a	10.623	4.447	3.402	4.877	2.14	n/a	n/a	n/a	1.668	n/a	1.977	n/a	0.5827
3E+06	15	1	1	2	10.578	1.635	8.537	5.023	3.528	4.218	1.604	n/a	n/a	n/a	2.596	n/a	2.223	n/a	0.8662
3E+06	15	1	1	1	11.295	n/a	8.856	2.731	2.688	4.009	0.962	n/a	n/a	n/a	1.07	n/a	1.011	n/a	0.2011
3E+06	15	1	1	1	3.764	n/a	10.163	2.944	3.692	5.71	2.688	n/a	n/a	n/a	1.52	n/a	1.641	n/a	0.494
3E+06	15	1	1	2	4.179	1.322	8.68	4.885	3.227	4.909	1.793	n/a	n/a	n/a	2.518	n/a	1.708	n/a	0.938
3E+06	16	1	1	1	0.661	n/a	8.595	3.271	2.837	5.275	2.566	n/a	n/a	n/a	1.593	n/a	1.449	n/a	0.2247
3E+06	16	1	1	1	8.717	n/a	9.829	3.371	2.768	4.197	2.957	n/a	n/a	n/a	1.78	n/a	1.727	n/a	0.5692
3E+06	16	1	1	2	8.569	2.335	11.059	4.057	3.163	4.209	1.735	n/a	n/a	n/a	1.682	n/a	2.476	n/a	0.6881
3E+06	16	1	1	1	6.718	n/a	10.906	4.172	3.247	6.897	3.398	n/a	n/a	n/a	2.414	n/a	1.691	n/a	0.6664
3E+06	16	1	1	1	4.913	n/a	7.371	4.197	2.731	4.456	0.864	n/a	n/a	n/a	1.12	n/a	1.23	n/a	0.0647
3E+06	16	1	1	1	3.368	n/a	8.203	4.009	3.114	5.615	2.586	n/a	n/a	n/a	1.73	n/a	2.146	n/a	0.9599
3E+06	16	2	2	2	10.287	1.938	7.188	5.811	3.754	3.902	1.434	3.561	4.516	1.443	1.259	0.782	1.818	1.151	0.8996
3E+06	16	1	1	2	7.062	1.727	7.757	4.49	2.957	5.536	1.931	n/a	n/a	n/a	1.957	n/a	2.238	n/a	0.9555
3E+06	16	1	1	1	10.417	n/a	10.801	3.532	3.247	3.983	2.335	n/a	n/a	n/a	1.037	n/a	1.703	n/a	0.4226
3E+06	16	1	1	1	3.517	n/a	7.757	3.532	2.801	3.961	1.674	n/a	n/a	n/a	1.674	n/a	1.658	n/a	0.1601
3E+06	16	1	1	1	5.801	n/a	9.252	3.517	2.726	5.164	2.109	n/a	n/a	n/a	1.718	n/a	1.968	n/a	0.5861
3E+06	16	1	1	1	7.921	n/a	11.001	3.825	3.013	4.661	2.443	n/a	n/a	n/a	1.822	n/a	1.671	n/a	0.5592
3E+06	16	1	1	1	1.931	n/a	7.387	3.163	3.586	4.261	1.764	n/a	n/a	n/a	0.986	n/a	1.232	n/a	0.06
3E+06	16	1	1	1	6.81	n/a	10.895	3.432	3.744	6.414	4.209	n/a	n/a	n/a	1.883	n/a	2.277	n/a	0.6808
3E+06	16	1	1	1	6.353	n/a	11.259	4.447	3.657	4.824	1.076	n/a	n/a	n/a	1.384	n/a	1.865	n/a	0.523
3E+06	16	1	1	1	7.757	n/a	10.734	3.586	3.383	5.793	3.207	n/a	n/a	n/a	1.948	n/a	1.515	n/a	0.5559
3E+06	16	1	1	1	7.84	n/a	11.564	3.502	3.882	6.188	2.926	n/a	n/a	n/a	2.357	n/a	2.324	n/a	0.7476
3E+06	16	1	1	1	13.251	n/a	12.718	3.223	3.371	5.352	2.74	n/a	n/a	n/a	3.001	n/a	2.011	n/a	0.7667
3E+06	16	1	1	1	2.964	n/a	8.907	3.66	3.521	4.38	1.076	n/a	n/a	n/a	1.638	n/a	1.191	n/a	0.2486
3E+06	17	1	1	1	3.163	n/a	9.016	2.948	2.448	4.527	2.313	n/a	n/a	n/a	0.826	n/a	1.784	n/a	0.246
3E+06	17	1	1	1	9.625	n/a	11.45	3.383	4.057	6.598	3.163	n/a	n/a	n/a	2.611	n/a	2.304	n/a	0.7783
3E+06	17	1	1	1	3.664	n/a	8.196	4.182	3.517	5.485	2.411	n/a	n/a	n/a	1.548	n/a	1.714	n/a	0.1959
3E+06	17	1	1	1	4.333	n/a	6.454	3.657	3.469	4.768	1.252	n/a	n/a	n/a	2.127	n/a	1.636	n/a	0.9511
3E+06	17	1	1	1	1.579	n/a	7.897	2.891	2.957	4.172	2.181	n/a	n/a	n/a	1.35	n/a	1.681	n/a	0.145
3E+06	17	1	1	1	0.962	n/a	6.464	4.683	2.279	3.097	1.771	n/a	n/a	n/a	1.254	n/a	1.386	n/a	0.0159
3E+06	17	1	1	1	4.932	n/a	8.971	3.08	3.183	6.822	2.427	n/a	n/a	n/a	1.429	n/a	1.331	n/a	0.254
3E+06	17	1	1	1	9.351	n/a	11.115	4.197	3.371	5.916	2.759	n/a	n/a	n/a	1.264	n/a	1.658	n/a	0.4625
3E+06	17	1	1	2	9.589	1.828	10.066	3.337	3.532	5.831	2.411	n/a	n/a	n/a	2.361	n/a	2.205	n/a	0.7514
3E+06	17	1	1	0	n/a	n/a	7.975	4.516	2.801	4.49	1.735	n/a	n/a	n/a	1.549	n/a	1.734	n/a	0.1804
3E+06	17	1	1	1	4.023	n/a	8.037	4.169	2.405	4.009	1.735	n/a	n/a	n/a	1.505	n/a	1.434	n/a	0.1626
3E+06	17	1	1	1	2.948	n/a	7.549	4.057	2.688	3.517	1.283	n/a	n/a	n/a	1.585	n/a	1.397	n/a	0.1128
3E+06	17	1	1	1	10.306	n/a	11.055	3.55	3.114	4.022	2.028	n/a	n/a	n/a	1.368	n/a	1.275	n/a	0.4135
3E+06	17	1	1	1	3.993	n/a	9.538	4.019	4.628	6.464	1.635	n/a	n/a	n/a	1.415	n/a	1.741	n/a	0.4909
3E+06	17	1	1	1	7.77	n/a	9.282	3.702	3.97	5.914	2.586	n/a	n/a	n/a	2.665	n/a	1.958	n/a	0.8296
3E+06	17	1	1	1	7.892	n/a	10.697	4.06	3.051	6.925	3.774	n/a	n/a	n/a	1.848	n/a	2.087	n/a	0.6309
3E+06	17	1	1	1	8.158	n/a	12.656	4.538	4.169	5.936	2.74	n/a	n/a	n/a	1.74	n/a	2.78	n/a	0.74
3E+06	17	1	1	1	8.603	n/a	7.764	4.107	3.862	5.134	2.505	n/a	n/a	n/a	1.859	n/a	1.199	n/a	0.135
3E+06	17	1	1	1	13.424	n/a	7.357	3.348	2.688	3.852	1.651	n/a	n/a	n/a	1.108	n/a	1.094	n/a	0.053
3E+06	17	1	1	1	14.11	n/a	10.233	3.306	2.443	5.448	3.195	n/a	n/a	n/a	0.992	n/a	1.387	n/a	0.3487
3E+06	18	1	1	1	6.028	n/a	10.434	3.692	3.618	4.516	1.814	n/a	n/a	n/a	1.348	n/a	1.289	n/a	0.3837
3E+06	18	1	1	2	8.071	3.302	7.864	4.493	2.768	4.123	1.771	n/a	n/a	n/a	1.496	n/a	2.01	n/a	0.9467
3E+06	18	1	1	1	8.708	n/a	9.083	3.915	3.409	5.916	2.091	n/a	n/a	n/a	1.603	n/a	1.499	n/a	0.3034
3E+06	18	1	1	1	7.332	n/a	13.952	3.175	3.302	5.567	2.448	n/a	n/a	n/a	1.98	n/a	2.182	n/a	0.7101
3E+06	18	2	2	2	5.937	2.934	7.223	5.688	3.114	3.657	1.727	2.009	3.561	1.076	1.282	1.574	1.361	1.351	0.8828
3E+06	18	1	1	1	4.907	n/a	6.925	3.785	2.384	3.469	1.361	n/a	n/a	n/a	1.189	n/a	1.216	n/a	0.0274
3E+06	18	1	2	2	10.515	3.517	7.539	5.522	2.61	5.221	2.296	n/a	4.541	2.251	2.58	n/a	1.197	1.203	0.8911
3E+06	18	1	1	1	9.847	n/a	7.392	4.182	3.915	3.879	1.951	n/a	n/a	n/a	1.37	n/a	1.252	n/a	0.0742
3E+06	18	1	1	1	7.371	n/a	10.676	3.469	3.026	7.539	4.138	n/a	n/a	n/a	1.872	n/a	2.03	n/a	0.6239
3E+06	18	1	1	1	9.228	n/a	7.028	3.983	2.957	3.657	1.027	n/a	n/a	n/a	1.147	n/a	1.06	n/a	0.0297
3E+06	18	1	1	1	0.453	n/a	9.397	3.785	3.692	5.71	2.586	n/a	n/a	n/a	2.58	n/a	2.241	n/a	0.8256
3E+06	18	1	1	1	6.342	n/a	12.079	2.904	3.657	6.328	2.904	n/a	n/a	n/a	1.999	n/a	2.14	n/a	0.6917
3E+06	18	1	1	2	5.055	1.027	9.494	4.467	2.891	4.768	2.151	n/a	n/a	n/a	3.016	n/a	2.762	n/a	0.8096
3E+06	18	1	1	2	9.464	1.252	9.707	3.774	2.74	5.314	1.604	n/a	n/a	n/a	2.308	n/a	2.41	n/a	0.8136

3E+06	18	1	1	1	8.871	n/a	12.929	3.08	2.726	5.613	3.114	n/a	n/a	n/a	1.137	n/a	1.481	n/a	0.4501
5E+06	1	1	1	1	12.558	n/a	11.029	2.453	2.489	4.377	2.448	n/a	n/a	n/a	1.929	n/a	2.215	n/a	0.6755
5E+06	1	1	1	1	5.947	n/a	8.681	2.427	3.085	4.628	1.771	n/a	n/a	n/a	0.844	n/a	1.355	n/a	0.2387
5E+06	1	1	1	1	5.727	n/a	6.398	3.402	3.047	3.812	1.434	n/a	n/a	n/a	1.239	n/a	1.096	n/a	0.0429
5E+06	1	2	2	2	8.457	2.731	6.543	5.536	1.793	3.047	1.293	2.296	3.247	1.221	1.193	1.472	1.119	1.111	0.9058
5E+06	1	1	1	1	7.711	n/a	7.785	2.586	2.654	4.675	2.61	n/a	n/a	n/a	0.743	n/a	1.102	n/a	0.1492
5E+06	1	1	1	1	6.541	n/a	6.487	2.028	2.181	3.195	1.014	n/a	n/a	n/a	0.774	n/a	1.233	n/a	0.0408
5E+06	1	1	1	1	9.903	n/a	9.67	2.489	3.026	5.236	2.51	n/a	n/a	n/a	1.258	n/a	1.611	n/a	0.3341
5E+06	1	1	1	1	10.627	n/a	8.38	2.891	2.63	3.66	1.793	n/a	n/a	n/a	1.345	n/a	1.082	n/a	0.2189
5E+06	1	1	1	1	8.79	n/a	10.896	2.181	2.768	4.919	2.51	n/a	n/a	n/a	1.113	n/a	1.321	n/a	0.383
5E+06	1	2	2	2	4.536	1.969	6.106	5.096	2.335	3.542	1.283	2.384	3.855	1.134	0.747	0.968	1.335	1.016	1
5E+06	1	2	2	2	10.988	4.098	7.261	4.76	2.855	3.73	1.221	2.948	4.06	1.37	0.782	0.656	0.962	1.269	0.9958
5E+06	1	1	1	1	11.38	n/a	10.654	2.745	3.207	4.49	1.931	n/a	n/a	n/a	1.244	n/a	1.074	n/a	0.3583
5E+06	1	1	1	1	7.462	n/a	11.102	2.731	3.243	6.045	2.855	n/a	n/a	n/a	0.906	n/a	1.175	n/a	0.3556
5E+06	1	1	1	1	7.094	n/a	10.875	2.535	3.517	6.138	2.842	n/a	n/a	n/a	1.603	n/a	1.946	n/a	0.5839
5E+06	1	1	1	0	6.152	n/a	6.408	3.114	2.745	3.636	1.764	n/a	n/a	n/a	0.854	n/a	0.906	n/a	0.0256
5E+06	1	1	2	2	4.419	0.661	7.872	4.877	2.805	3.879	1.735	n/a	4.458	1.931	1.698	n/a	1.257	0.973	0.9916
5E+06	1	1	1	1	7.512	n/a	9.648	3.097	2.571	4.473	2.313	n/a	n/a	n/a	0.765	n/a	1.014	n/a	0.2999
5E+06	1	1	1	1	8.06	n/a	9.981	2.869	2.745	5.289	2.944	n/a	n/a	n/a	1.343	n/a	1.782	n/a	0.4888
5E+06	1	1	1	1	9.962	n/a	8.787	2.51	2.51	4.749	2.091	n/a	n/a	n/a	0.951	n/a	0.838	n/a	0.2164
5E+06	1	1	1	2	7.588	1.814	7.441	4.683	2.51	4.098	1.705	n/a	n/a	n/a	1.819	n/a	2.266	n/a	0.9874
5E+06	1	1	1	1	10.174	n/a	7.864	2.74	2.731	3.618	1.252	n/a	n/a	n/a	1.214	n/a	0.85	n/a	0.1586
5E+06	1	1	1	1	12.538	n/a	13.265	3.114	2.768	5.455	3.064	n/a	n/a	n/a	2.292	n/a	1.856	n/a	0.7028
5E+06	2	1	1	1	7.128	n/a	11.354	2.948	3.08	5.742	2.801	n/a	n/a	n/a	1.945	n/a	2.033	n/a	0.6521
5E+06	2	1	1	1	10.813	n/a	8.893	2.181	2.948	3.306	1.479	n/a	n/a	n/a	1.263	n/a	1.345	n/a	0.2843
5E+06	2	1	1	1	10.455	n/a	10.691	3.013	2.948	4.768	2.688	n/a	n/a	n/a	2.613	n/a	2.169	n/a	0.7916
5E+06	2	1	2	2	6.62	3.05	6.766	4.981	2.983	4.561	1.735	n/a	4.458	2.151	2.895	n/a	0.923	1.019	0.9098
5E+06	2	1	1	1	10.046	n/a	6.301	3.306	3.306	3.983	1.134	n/a	n/a	n/a	1.128	n/a	0.975	n/a	0.0235
5E+06	2	1	2	2	5.725	3.432	6.683	5.033	2.495	3.064	2.091	n/a	3.915	1.938	1.784	n/a	1.627	1.101	0.942
5E+06	2	1	1	1	12.211	n/a	11.097	3.08	2.801	4.424	1.727	n/a	n/a	n/a	1.418	n/a	1.284	n/a	0.408
5E+06	2	1	1	1	20.724	n/a	13.271	3.383	3.207	6.127	2.535	n/a	n/a	n/a	2.647	n/a	2.809	n/a	0.777
5E+06	2	1	1	1	5.64	n/a	6.013	3.08	2.63	3.432	1.579	n/a	n/a	n/a	1.275	n/a	1.07	n/a	0.0127
5E+06	2	1	1	1	4.896	n/a	7.779	3.051	3.183	4.719	2.368	n/a	n/a	n/a	1.381	n/a	1.414	n/a	0.1896
5E+06	2	1	1	1	16.142	n/a	14.01	3.692	2.842	4.909	2.489	n/a	n/a	n/a	1.611	n/a	2.063	n/a	0.5807
5E+06	2	1	1	1	5.071	n/a	7.459	3.195	2.855	3.517	1.521	n/a	n/a	n/a	1.413	n/a	1.117	n/a	0.1422
5E+06	2	1	1	1	8.376	n/a	11.381	2.855	3.114	5.388	2.61	n/a	n/a	n/a	2.442	n/a	2.42	n/a	0.7734
5E+06	2	1	1	1	1.793	n/a	9.912	3.72	2.61	4.362	1.828	n/a	n/a	n/a	1.569	n/a	1.63	n/a	0.5067
5E+06	2	1	1	1	12.051	n/a	8.288	2.745	2.669	4.088	1.983	n/a	n/a	n/a	1.057	n/a	1.402	n/a	0.2115
5E+06	2	1	1	1	5.708	n/a	6.495	3.195	2.411	3.618	0.935	n/a	n/a	n/a	1.519	n/a	1.04	n/a	0.0649
5E+06	2	1	1	1	4.354	n/a	6.962	3.443	2.842	3.409	1.283	n/a	n/a	n/a	0.912	n/a	1.144	n/a	0.0872
5E+06	2	1	1	1	8.414	n/a	10.292	3.085	3.085	5.426	2.384	n/a	n/a	n/a	1.539	n/a	1.864	n/a	0.5588
5E+06	2	1	1	1	12.079	n/a	11.231	3.744	2.801	4.592	2.009	n/a	n/a	n/a	1.138	n/a	1.382	n/a	0.3996
5E+06	2	1	1	1	9.659	n/a	9.99	2.384	2.891	5.204	1.793	n/a	n/a	n/a	1.081	n/a	1.155	n/a	0.3209
5E+06	2	1	1	1	6.887	n/a	11.376	2.759	3.306	5.164	2.181	n/a	n/a	n/a	2.197	n/a	2.031	n/a	0.6857
5E+06	2	1	1	1	8.011	n/a	10.884	2.886	3.383	5.096	1.877	n/a	n/a	n/a	1.434	n/a	1.343	n/a	0.4164
5E+06	2	1	1	1	7.166	n/a	8.639	3.247	3.432	4.661	1.479	n/a	n/a	n/a	1.068	n/a	1.28	n/a	0.2462
5E+06	2	1	1	1	6.912	n/a	10.11	3.064	2.731	4.564	2.335	n/a	n/a	n/a	1.812	n/a	1.855	n/a	0.5998
5E+06	2	1	1	1	10.454	n/a	10.809	3.08	2.996	5.236	2.313	n/a	n/a	n/a	2.041	n/a	1.941	n/a	0.6488
5E+06	3	1	1	1	7.209	n/a	9.78	2.768	2.63	5.289	2.571	n/a	n/a	n/a	1.769	n/a	1.413	n/a	0.5037
5E+06	3	2	2	2	7.838	2.547	6.886	5.39	3.183	3.852	1.283	3.064	4.558	1.434	1.237	1.657	1.04	0.842	0.9138
5E+06	3	1	1	1	9.854	n/a	15.157	3.744	3.227	6.384	3.337	n/a	n/a	n/a	1.8	n/a	2.019	n/a	0.6722
5E+06	3	1	1	1	14.039	n/a	11.356	2.251	2.63	5.033	2.411	n/a	n/a	n/a	1.524	n/a	1.338	n/a	0.4536
5E+06	3	2	2	2	6.281	4.88	7.216	5.033	2.233	2.926	0.907	2.368	3.517	0.962	0.886	0.755	1.198	1.139	0.9832
5E+06	3	1	1	1	8.57	n/a	10.205	4.107	3.195	5.688	2.591	n/a	n/a	n/a	1.496	n/a	1.543	n/a	0.4594
5E+06	3	1	1	2	10.113	6.986	9.584	3.862	3.085	4.377	1.877	n/a	n/a	n/a	1.785	n/a	1.659	n/a	0.5713
5E+06	3	1	1	1	3.37	n/a	11.386	3.159	2.957	4.814	2.443	n/a	n/a	n/a	2.009	n/a	1.8	n/a	0.6225
5E+06	3	1	1	1	6.579	n/a	10.324	3.243	3.371	4.592	1.931	n/a	n/a	n/a	2.559	n/a	2.374	n/a	0.8212
5E+06	3	1	1	1	5.424	n/a	5.56	3.657	3.243	3.08	1.793	n/a	n/a	n/a	1.175	n/a	1.637	n/a	0.0064
5E+06	3	1	1	1	11.074	n/a	6.822	3.097	2.61	3.66	1.293	n/a	n/a	n/a	1.16	n/a	1.254	n/a	0.0894
5E+06	3	1	1	1	3.927	n/a	7.232	3.337	2.948	4.022	1.727	n/a	n/a	n/a	1.445	n/a	1.16	n/a	0.1236
5E+06	3	1	1	1	1.252	n/a	9.451	2.842	2.837	3.66	1.283	n/a	n/a	n/a	1.353	n/a	1.313	n/a	0.3103
5E+06	3	1	1	1	0.16	n/a	11.383	3.561	3.443	5.181	1.793	n/a	n/a	n/a	1.738	n/a	1.698	n/a	0.5525
5E+06	3	2	2	1	6.548	n/a	9.251	3.432	3.013	5.164	2.505	n/a	n/a	n/a	1.001	1.038	1.375	1.24	0.84
5E+06	3	1	1	1	12.264	n/a	12.872	3.561	2.721	4.022	2.085	n/a	n/a	n/a	1.16	n/a	1.481	n/a	0.4507
5E+06	3	1	1	1	11.451	n/a	7.559	3.159	3.502	4.33	0.935	n/a	n/a	n/a	1.289	n/a	1.325	n/a	0.1657

5E+06	3	1	1	1	9.493	n/a	8.881	2.855	2.233	4.516	1.983	n/a	n/a	n/a	1.058	n/a	1.111	n/a	0.2588
5E+06	3	1	1	1	10.627	n/a	9.467	4.653	3.879	5.023	1.443	n/a	n/a	n/a	0.965	n/a	1.716	n/a	0.3156
5E+06	4	1	1	1	12.85	n/a	10.886	3.348	3.08	4.11	1.452	n/a	n/a	n/a	1.159	n/a	1.132	n/a	0.3692
5E+06	4	1	2	2	10.229	3.17	6.328	5.012	2.768	3.825	1.938	n/a	3.657	2.009	1.991	n/a	1.038	0.868	0.979
5E+06	4	1	1	1	12.309	n/a	10.73	3.702	2.869	5.601	3.047	n/a	n/a	n/a	2.156	n/a	1.963	n/a	0.6654
5E+06	4	1	1	1	8.322	n/a	10.423	2.405	2.74	5.338	2.495	n/a	n/a	n/a	1.073	n/a	1.274	n/a	0.3529
5E+06	4	1	1	2	11.902	2.566	10.701	2.669	2.886	4.412	2.181	n/a	n/a	n/a	2.062	n/a	1.878	n/a	0.6454
5E+06	4	1	1	1	8.502	n/a	10.948	2.63	3.08	5.34	2.63	n/a	n/a	n/a	1.768	n/a	1.571	n/a	0.534
5E+06	4	1	1	2	13.028	2.505	11.697	3.026	3.163	4.675	1.513	n/a	n/a	n/a	2.299	n/a	2.337	n/a	0.7412
5E+06	4	1	1	1	4.527	n/a	7.429	2.891	3.586	4.716	1.379	n/a	n/a	n/a	1.009	n/a	1.868	n/a	0.1728
5E+06	4	1	1	1	4.574	n/a	6.287	2.891	2.405	3.692	1.293	n/a	n/a	n/a	1.017	n/a	1.562	n/a	0.0386
5E+06	4	2	2	2	6.577	2.832	6.612	6.272	3.013	3.688	1.361	3.151	4.088	1.87	1.326	0.8	1.005	1.195	0.9748
5E+06	4	1	1	1	5.713	n/a	6.464	2.586	2.801	3.852	1.434	n/a	n/a	n/a	1.548	n/a	1.053	n/a	0.0627
5E+06	4	1	1	1	20.026	n/a	14.057	3.517	3.785	6.39	3.051	n/a	n/a	n/a	2.017	n/a	2.319	n/a	0.7167
5E+06	5	1	1	1	8.273	n/a	11.453	2.957	2.768	5.649	3.085	n/a	n/a	n/a	2.554	n/a	2.04	n/a	0.7377
5E+06	5	1	1	1	15.588	n/a	10.603	3.862	3.586	5.141	1.924	n/a	n/a	n/a	2.339	n/a	2.266	n/a	0.7518
5E+06	5	1	1	1	4.991	n/a	11.03	3.085	3.097	4.989	2.654	n/a	n/a	n/a	1.455	n/a	1.821	n/a	0.5279
5E+06	5	1	1	1	13.333	n/a	10.922	2.731	3.08	5.164	2.745	n/a	n/a	n/a	2.433	n/a	2.437	n/a	0.7989
5E+06	5	1	1	1	7.059	n/a	9.173	4.716	2.948	5.485	2.448	n/a	n/a	n/a	1.709	n/a	1.874	n/a	0.5966
5E+06	5	1	1	1	10.263	n/a	10.589	3.064	3.085	5.154	1.924	n/a	n/a	n/a	1.772	n/a	2.04	n/a	0.616
5E+06	5	1	1	1	11.932	n/a	12.173	3.051	3.207	5.951	3.013	n/a	n/a	n/a	1.243	n/a	1.599	n/a	0.4711
5E+06	6	1	1	1	4.122	n/a	7.631	2.091	2.745	4.197	1.521	n/a	n/a	n/a	0.756	n/a	1.284	n/a	0.1375
5E+06	6	1	1	1	4.469	n/a	7.063	3.085	2.891	4.362	1.764	n/a	n/a	n/a	1.544	n/a	1.289	n/a	0.119
5E+06	6	1	1	1	6.64	n/a	7.515	3.636	2.731	4.784	1.735	n/a	n/a	n/a	1.049	n/a	1.343	n/a	0.1398
5E+06	6	1	1	2	8.25	2.296	7.618	4.784	2.869	3.983	1.434	n/a	n/a	n/a	2.278	n/a	2.742	n/a	0.8745
5E+06	6	1	1	1	9.781	n/a	9.184	3.085	2.571	4.318	1.828	n/a	n/a	n/a	0.751	n/a	1.282	n/a	0.2792
5E+06	6	1	1	1	12.671	n/a	10.888	2.721	2.801	5.793	3.026	n/a	n/a	n/a	1.892	n/a	1.748	n/a	0.5934
5E+06	6	1	1	1	6.792	n/a	8.754	3.085	2.891	5.236	3.08	n/a	n/a	n/a	0.99	n/a	1.004	n/a	0.2288
5E+06	6	1	1	1	6.267	n/a	9.239	3.183	2.61	4.447	2.411	n/a	n/a	n/a	0.929	n/a	1.172	n/a	0.2895
5E+06	6	1	1	1	10.857	n/a	8.941	3.604	2.688	4.234	1.983	n/a	n/a	n/a	0.646	n/a	1.241	n/a	0.2512
5E+06	6	1	1	1	7.553	n/a	11.426	2.566	3.502	5.511	2.586	n/a	n/a	n/a	1.479	n/a	1.553	n/a	0.474
5E+06	6	2	2	2	10.693	4.291	7.123	4.94	2.054	3.72	1.379	2.886	3.383	1.674	1.146	0.832	1.378	1.375	0.9178
5E+06	6	1	1	1	12.571	n/a	11.997	2.957	3.383	6.127	3.604	n/a	n/a	n/a	0.883	n/a	1.06	n/a	0.3857
5E+06	6	1	1	1	14.015	n/a	10.391	2.904	2.768	4.516	2.731	n/a	n/a	n/a	0.793	n/a	1.093	n/a	0.3288
5E+06	6	1	1	1	11.416	n/a	10.816	3.043	3.211	4.811	2.411	n/a	n/a	n/a	0.841	n/a	1.471	n/a	0.3665
5E+06	6	2	2	2	8.641	5.801	6.305	4.172	2.745	3.462	0.661	3.159	4.169	1.379	1.135	0.691	0.772	1.002	0.9707
5E+06	6	1	1	1	5.622	n/a	7.897	3.55	2.801	4.974	2.384	n/a	n/a	n/a	0.969	n/a	1.021	n/a	0.1562
5E+06	6	1	1	1	4.695	n/a	7.89	2.721	2.801	3.862	1.828	n/a	n/a	n/a	0.968	n/a	1.136	n/a	0.1705
5E+06	6	1	1	1	9.509	n/a	6.094	3.211	2.669	3.72	3.085	n/a	n/a	n/a	0.754	n/a	1.101	n/a	0.0085
5E+06	7	1	1	1	11.902	n/a	8.934	3.371	3.271	5.311	2.448	n/a	n/a	n/a	1.404	n/a	1.402	n/a	0.2947
5E+06	7	1	1	1	6.925	n/a	12.733	2.571	3.383	5.853	2.109	n/a	n/a	n/a	2.447	n/a	1.894	n/a	0.7097
5E+06	7	1	1	1	9.011	n/a	7.261	2.957	2.251	4.561	2.443	n/a	n/a	n/a	1.07	n/a	1.186	n/a	0.1144
5E+06	7	1	1	1	8.913	n/a	6.53	3.825	3.211	4.138	2.453	n/a	n/a	n/a	1.134	n/a	1.246	n/a	0.0605
5E+06	7	1	1	1	10.604	n/a	11.134	2.869	3.064	4.653	1.612	n/a	n/a	n/a	1.541	n/a	1.362	n/a	0.4565
5E+06	7	1	1	1	3.586	n/a	6.53	2.296	2.688	3.855	1.076	n/a	n/a	n/a	1.13	n/a	1.184	n/a	0.0583
5E+06	7	1	1	1	1.479	n/a	9.231	2.74	2.768	4.022	1.379	n/a	n/a	n/a	1.023	n/a	1.224	n/a	0.2921
5E+06	7	1	1	1	19.1	n/a	11.911	3.064	2.837	4.493	1.793	n/a	n/a	n/a	1.299	n/a	1.617	n/a	0.4682
5E+06	7	1	1	1	13.673	n/a	10.399	3.454	2.384	3.744	1.635	n/a	n/a	n/a	1.466	n/a	1.159	n/a	0.3747
5E+06	7	1	1	1	16.821	n/a	11.139	4.197	3.097	5.462	2.904	n/a	n/a	n/a	1.03	n/a	1.589	n/a	0.4052
5E+06	7	1	1	1	7.585	n/a	10.097	3.521	2.944	4.994	3.247	n/a	n/a	n/a	1.686	n/a	1.57	n/a	0.5158
5E+06	7	1	1	1	0.975	n/a	9.829	3.051	2.51	4.403	2.109	n/a	n/a	n/a	1.31	n/a	1.402	n/a	0.3395
5E+06	7	1	1	1	3.459	n/a	7.711	2.801	2.731	4.06	1.322	n/a	n/a	n/a	0.837	n/a	1.141	n/a	0.1468
5E+06	7	1	1	1	13.608	n/a	7.701	3.383	2.091	3.371	1.793	n/a	n/a	n/a	1.074	n/a	1.283	n/a	0.1633
5E+06	7	1	1	1	11.5	n/a	12.772	3.013	3.183	5.594	2.801	n/a	n/a	n/a	1.933	n/a	2.295	n/a	0.6994
5E+06	7	1	1	0	n/a	n/a	5.992	4.473	1.651	3.064	2.028	n/a	n/a	n/a	1.113	n/a	1.519	n/a	0.017
5E+06	7	1	1	1	11.395	n/a	6.227	3.561	2.505	3.744	1.027	n/a	n/a	n/a	1.42	n/a	1.017	n/a	0.0299
5E+06	7	1	2	2	10.01	3.159	8.856	4.356	2.996	3.931	1.513	n/a	4.362	1.705	1.886	n/a	1.264	1.44	0.8514
5E+06	7	1	1	1	11.702	n/a	10.682	2.571	3.043	5.757	2.571	n/a	n/a	n/a	2.1	n/a	1.707	n/a	0.6127
5E+06	8	1	1	1	5.719	n/a	9.981	2.944	2.891	4.95	2.228	n/a	n/a	n/a	1.344	n/a	1.29	n/a	0.3502
5E+06	8	1	1	0	n/a	n/a	8.481	2.384	2.489	3.561	0.962	n/a	n/a	n/a	1.135	n/a	1.277	n/a	0.226
5E+06	8	1	1	1	5.48	n/a	6.545	3.013	2.855	2.837	0.935	n/a	n/a	n/a	0.57	n/a	1.207	n/a	0.0342
5E+06	8	1	1	1	14.251	n/a	10.143	2.957	2.891	4.888	2.368	n/a	n/a	n/a	1.265	n/a	1.518	n/a	0.3802
5E+06	8	1	1	1	10.098	n/a	11.671	3.657	3.085	5.033	2.335	n/a	n/a	n/a	2.209	n/a	2.06	n/a	0.696
5E+06	8	1	1	1	11.559	n/a	10.976	3.55	2.891	3.905	1.635	n/a	n/a	n/a	1.648	n/a	1.49	n/a	0.4918
5E+06	8	1	1	1	10.613	n/a	6.581	3.409	2.61	3.517	0.907	n/a	n/a	n/a	1.014	n/a	1.101	n/a	0.0561

5E+06	8	1	1	1	12.962	n/a	10.021	3.247	2.957	5.448	2.313	n/a	n/a	n/a	2.705	n/a	2.353	n/a	0.8287
5E+06	8	1	1	2	7.7	1.37	10.734	3.528	3.207	5.134	2.181	n/a	n/a	n/a	2.853	n/a	2.222	n/a	0.8063
5E+06	8	1	1	1	1.958	n/a	6.589	2.535	2.688	4.012	1.651	n/a	n/a	n/a	1.475	n/a	1.005	n/a	0.0716
5E+06	8	1	1	1	7.949	n/a	11.046	3.247	2.669	4.738	2.296	n/a	n/a	n/a	1.925	n/a	1.972	n/a	0.6388
5E+06	8	1	1	1	9.296	n/a	9.967	2.745	2.721	4.237	2.566	n/a	n/a	n/a	1.157	n/a	1.161	n/a	0.3262
5E+06	8	1	1	1	12.831	n/a	12.655	3.561	2.926	5.376	2.74	n/a	n/a	n/a	1.783	n/a	1.995	n/a	0.6257
5E+06	8	1	2	2	7.647	3.143	7.207	4.953	3.026	4.06	1.958	n/a	4.009	1.293	2.203	n/a	1.169	1.642	0.8901
5E+06	8	1	1	1	13.02	n/a	9.173	3.064	2.926	4.197	2.571	n/a	n/a	n/a	1.05	n/a	1.444	n/a	0.2973
5E+06	8	1	1	1	9.123	n/a	7.937	4.197	3.561	6.254	2.586	n/a	n/a	n/a	1.447	n/a	1.498	n/a	0.2091
5E+06	8	1	1	1	6.421	n/a	8.464	3.051	2.453	4.784	2.654	n/a	n/a	n/a	0.562	n/a	1.382	n/a	0.1969
5E+06	8	1	1	1	12.477	n/a	11.259	2.957	2.904	5.567	3.227	n/a	n/a	n/a	2.037	n/a	1.978	n/a	0.6587
5E+06	8	1	1	1	5.509	n/a	7.908	2.411	2.957	4.869	2.745	n/a	n/a	n/a	1.455	n/a	1.308	n/a	0.192
5E+06	9	1	2	2	7.312	2.842	8.058	4.169	3.368	2.944	4.473	n/a	n/a	4.009	2.133	n/a	0.83	1.267	0.9665
5E+06	9	1	1	1	6.2	n/a	10.691	3.183	3.207	5.71	2.448	n/a	n/a	n/a	2.289	n/a	2.035	n/a	0.6891
5E+06	9	1	1	1	4.819	n/a	5.253	2.228	2.837	4.019	1.764	n/a	n/a	n/a	1.877	n/a	1.176	n/a	0.0021
5E+06	9	2	2	2	7.828	3.542	6.653	5.194	2.805	3.882	1.37	2.731	3.271	1.252	1.107	1.81	1.146	1.21	0.8862
5E+06	9	1	1	1	9.783	n/a	8.696	3.702	3.306	4.919	1.452	n/a	n/a	n/a	1.709	n/a	1.136	n/a	0.2741
5E+06	9	1	2	2	7.353	2.427	7.462	5.063	2.586	3.73	1.604	n/a	4.057	2.009	2.127	n/a	1.616	1.316	0.8784
5E+06	9	1	1	1	6.566	n/a	7.674	3.08	2.251	4.953	2.948	n/a	n/a	n/a	1.356	n/a	1.358	n/a	0.1776
5E+06	9	1	1	1	9.172	n/a	10.897	2.944	2.535	4.76	2.61	n/a	n/a	n/a	1.52	n/a	1.33	n/a	0.4278
5E+06	9	1	1	2	12.616	0.818	11.396	2.731	3.085	5.455	2.181	n/a	n/a	n/a	2.061	n/a	1.567	n/a	0.5903
5E+06	9	1	1	1	6.819	n/a	11.097	3.862	3.302	5.253	2.505	n/a	n/a	n/a	1.694	n/a	1.805	n/a	0.5681
5E+06	9	1	1	1	1.793	n/a	9.498	3.462	3.064	4.822	2.74	n/a	n/a	n/a	2.578	n/a	2.61	n/a	0.8324
5E+06	9	1	1	1	6.013	n/a	7.531	3.163	2.61	4.261	1.771	n/a	n/a	n/a	1.052	n/a	1.172	n/a	0.1352
5E+06	9	1	1	1	6.088	n/a	8.565	2.948	3.013	4.234	1.735	n/a	n/a	n/a	1.334	n/a	1.154	n/a	0.2437
5E+06	9	2	2	2	13.572	5.412	6.543	6.062	2.443	3.409	1.134	2.443	3.306	0.962	1.298	1.174	0.807	0.914	0.9624
5E+06	9	1	1	1	8.871	n/a	10.562	2.51	2.505	4.877	2.535	n/a	n/a	n/a	1.331	n/a	1.318	n/a	0.3913
5E+06	9	1	1	2	6.05	2.726	10.781	3.247	3.08	5.36	2.157	n/a	n/a	n/a	3.023	n/a	2.516	n/a	0.8249
5E+06	9	1	1	1	8.809	n/a	8.738	4.197	2.181	4.047	2.571	n/a	n/a	n/a	1.23	n/a	1.195	n/a	0.2563
5E+06	9	1	1	1	11.932	n/a	10.556	2.801	2.535	5.164	2.842	n/a	n/a	n/a	1.704	n/a	1.562	n/a	0.5218
5E+06	9	1	1	2	7.904	1.958	10.787	2.891	3.227	5.063	2.268	n/a	n/a	n/a	1.972	n/a	2.036	n/a	0.6554
5E+06	9	1	1	1	8.916	n/a	6.962	4.057	2.535	3.852	2.091	n/a	n/a	n/a	1.003	n/a	1.32	n/a	0.094
5E+06	9	1	1	1	6.472	n/a	6.14	2.731	2.505	3.812	1.452	n/a	n/a	n/a	0.985	n/a	1.022	n/a	0.0106
5E+06	9	1	1	1	6.626	n/a	7.564	3.368	3.013	4.538	1.587	n/a	n/a	n/a	0.679	n/a	1.448	n/a	0.1329
5E+06	9	1	1	1	6.922	n/a	11.073	3.223	3.051	5.221	2.028	n/a	n/a	n/a	1.619	n/a	1.748	n/a	0.5402
5E+06	9	1	1	1	15.423	n/a	14.405	4.182	2.855	5.462	2.443	n/a	n/a	n/a	1.809	n/a	1.863	n/a	0.5776
5E+06	10	1	1	1	5.581	n/a	7.506	2.74	2.296	3.879	1.293	n/a	n/a	n/a	1.569	n/a	1.103	n/a	0.161
5E+06	10	1	1	1	9.014	n/a	10.514	3.114	2.886	4.869	2.63	n/a	n/a	n/a	1.394	n/a	1.588	n/a	0.4478
5E+06	10	1	1	1	4.333	n/a	7.063	3.383	2.944	4.853	2.384	n/a	n/a	n/a	1.049	n/a	1.252	n/a	0.0985
5E+06	10	1	1	1	9.059	n/a	6.645	2.801	2.891	3.692	1.604	n/a	n/a	n/a	1.28	n/a	1.145	n/a	0.076
5E+06	10	1	1	1	4.677	n/a	9.131	2.731	3.428	3.754	1.027	n/a	n/a	n/a	1.136	n/a	1.056	n/a	0.2818
5E+06	10	2	2	2	11.741	1.828	7.82	4.768	3.043	4.197	1.221	3.383	5.141	1.521	1.114	1.666	1.259	1.187	0.8629
5E+06	10	1	1	1	5.948	n/a	7.803	2.489	2.296	4.246	1.252	n/a	n/a	n/a	1.071	n/a	1.087	n/a	0.1539
5E+06	10	2	2	1	3.517	n/a	7.864	3.207	3.085	4.362	1.379	n/a	n/a	n/a	1.075	1.055	1.221	1.275	0.8979
5E+06	10	1	1	1	4.639	n/a	6.645	3.306	2.842	4.261	1.651	n/a	n/a	n/a	1.632	n/a	0.967	n/a	0.0827
5E+06	10	1	1	1	10.684	n/a	9.526	3.163	3.085	4.303	1.521	n/a	n/a	n/a	0.942	n/a	1.089	n/a	0.3025
5E+06	10	1	1	1	8.305	n/a	11.568	3.306	3.409	5.462	2.443	n/a	n/a	n/a	2.601	n/a	2.189	n/a	0.7662
5E+06	10	1	1	1	12.183	n/a	11.525	3.692	3.306	5.669	2.645	n/a	n/a	n/a	2.124	n/a	1.951	n/a	0.6688
5E+06	10	1	1	1	4.907	n/a	6.498	2.842	3.085	4.493	1.443	n/a	n/a	n/a	1.159	n/a	1.068	n/a	0.0517
5E+06	10	1	1	1	9.409	n/a	7.357	3.398	2.61	3.664	1.604	n/a	n/a	n/a	0.888	n/a	1.231	n/a	0.1167
5E+06	10	1	1	1	18.97	n/a	14.279	3.443	3.628	5.968	3.085	3.175	6.028	3.243	2.037	n/a	2.113	n/a	0.6925
5E+06	11	1	1	1	6.621	n/a	10.729	2.63	2.586	4.877	2.384	n/a	n/a	n/a	1.39	n/a	1.377	n/a	0.4108
5E+06	11	1	1	1	6.49	n/a	8.499	2.944	2.091	4.088	1.931	n/a	n/a	n/a	1.189	n/a	1.107	n/a	0.2213
5E+06	11	1	1	2	15.942	3.207	14.588	3.247	2.957	5.667	3.207	n/a	n/a	n/a	3.568	n/a	2.122	n/a	0.7698
5E+06	11	2	2	2	7.089	3.857	6.837	5.443	2.296	3.195	1.014	2.842	3.454	1.027	1.269	0.809	1.121	1.472	0.9218
5E+06	11	1	1	1	8.443	n/a	8.076	2.926	2.335	3.702	1.793	n/a	n/a	n/a	1.442	n/a	1.184	n/a	0.2017
5E+06	11	2	2	2	10.984	5.209	6.623	5.936	2.586	3.271	1.076	2.745	3.73	1.134	1.293	0.679	1.155	1.395	0.9379
5E+06	11	1	1	1	10.711	n/a	10.945	3.183	2.669	4.527	2.109	n/a	n/a	n/a	1.565	n/a	1.207	n/a	0.4136
5E+06	11	1	1	1	10.708	n/a	8.665	3.183	2.505	4.493	2.009	n/a	n/a	n/a	1.238	n/a	1.403	n/a	0.2613
5E+06	11	1	1	1	11.025	n/a	11.435	2.591	2.645	4.805	2.041	n/a	n/a	n/a	1.441	n/a	1.364	n/a	0.4335
5E+06	11	1	1	1	10.249	n/a	12.694	2.948	3.163	6.254	2.842	n/a	n/a	n/a	1.709	n/a	1.676	n/a	0.5371
5E+06	11	1	1	1	6.211	n/a	7.343	3.702	2.586	4.082	1.579	n/a	n/a	n/a	1.114	n/a	1.157	n/a	0.1213
5E+06	11	1	1	1	5.239	n/a	9.707	3.013	2.837	4.082	1.651	n/a	n/a	n/a	1.103	n/a	1.487	n/a	0.3235
5E+06	12	2	2	2	6.588	3.384	6.918	5.104	2.855	3.402	0.864	2.957	3.517	0.864	0.733	1.496	1.175	1.077	0.9338
5E+06	12	1	1	1	5.296	n/a	6.227	2.405	2.51	4.182	1.828	n/a	n/a	n/a	1.073	n/a	1.123	n/a	0.0192

5E+06	12	1	1	1	9.429	n/a	6.464	4.261	2.535	2.405	1.434	n/a	n/a	n/a	1.363	n/a	1.074	n/a	0.0539
5E+06	12	1	1	1	15.899	n/a	14.66	3.74	2.855	5.289	3.383	n/a	n/a	n/a	1.936	n/a	1.802	n/a	0.629
5E+06	12	1	1	1	5.417	n/a	7.557	3.348	2.688	4.356	1.983	n/a	n/a	n/a	1.056	n/a	1.278	n/a	0.1445
5E+06	12	1	1	1	5.781	n/a	6.287	3.175	2.157	2.891	1.604	n/a	n/a	n/a	1.264	n/a	1.241	n/a	0.0321
5E+06	12	1	1	1	11.277	n/a	11.122	3.29	3.085	5.104	2.591	n/a	n/a	n/a	2.1	n/a	1.709	n/a	0.6192
5E+06	12	1	1	2	7.939	0.818	10.801	3.243	3.026	5.631	2.745	n/a	n/a	n/a	1.769	n/a	1.335	n/a	0.4829
5E+06	12	1	1	1	9.918	n/a	7.671	2.51	2.296	3.195	1.134	n/a	n/a	n/a	1.565	n/a	0.996	n/a	0.1752
5E+06	12	1	1	1	9.04	n/a	8.652	2.251	2.842	5.033	2.891	n/a	n/a	n/a	1.172	n/a	1.267	n/a	0.2538
5E+06	12	1	1	2	8.669	1.156	11.17	3.183	2.61	4.762	1.479	n/a	n/a	n/a	2.298	n/a	2.65	n/a	0.7952
5E+06	12	1	1	1	1.027	n/a	9.471	3.66	2.891	4.209	1.877	n/a	n/a	n/a	1.405	n/a	1.778	n/a	0.5007
5E+06	12	1	1	1	11.607	n/a	9.985	3.013	2.891	4.768	2.181	n/a	n/a	n/a	2.46	n/a	1.859	n/a	0.6789
5E+06	12	1	1	1	7.285	n/a	8.982	3.051	2.571	5.379	2.268	n/a	n/a	n/a	1.154	n/a	1.086	n/a	0.2715
5E+06	12	1	1	1	12.901	n/a	12.571	3.302	3.085	4.661	1.951	n/a	n/a	n/a	2.601	n/a	2.718	n/a	0.7843
5E+06	12	1	1	1	9.088	n/a	9.725	2.957	3.383	5.793	2.427	n/a	n/a	n/a	2.052	n/a	1.383	n/a	0.565
5E+06	12	2	2	2	7.148	3.114	6.488	5.518	2.091	3.428	1.521	2.384	3.195	1.014	1.062	0.547	1.439	1.377	0.9583
5E+06	12	1	1	1	8.602	n/a	9.287	2.586	2.731	4.209	1.513	n/a	n/a	n/a	1.471	n/a	1.093	n/a	0.3051
5E+06	12	1	1	1	3.749	n/a	8.469	2.801	2.384	3.74	1.434	n/a	n/a	n/a	1.146	n/a	1.442	n/a	0.2412
5E+06	12	1	1	1	9.221	n/a	8.621	2.495	2.855	3.882	1.479	n/a	n/a	n/a	1.139	n/a	1.135	n/a	0.2337
5E+06	12	1	1	1	6.031	n/a	10.204	3.026	3.064	5.194	1.938	n/a	n/a	n/a	2.058	n/a	1.431	n/a	0.5744
5E+06	12	1	2	2	9.126	4.826	6.301	4.784	2.443	3.085	0.907	n/a	2.904	1.604	1.21	n/a	1.339	0.898	0.9542
5E+06	12	1	1	1	12.317	n/a	8.577	2.948	3.026	4.303	1.87	n/a	n/a	n/a	1.297	n/a	1.208	n/a	0.2487
5E+06	12	1	1	1	12.089	n/a	10.552	2.489	2.61	4.661	2.091	n/a	n/a	n/a	2.102	n/a	1.664	n/a	0.6063
5E+06	12	1	1	1	8.409	n/a	10.367	2.768	2.571	4.318	1.938	n/a	n/a	n/a	1.765	n/a	1.406	n/a	0.4977
5E+06	12	1	1	1	6.714	n/a	8.856	2.957	2.448	5.164	2.489	n/a	n/a	n/a	1.207	n/a	1.209	n/a	0.2664
5E+06	12	1	1	1	7.598	n/a	11.083	3.051	3.383	5.134	2.268	n/a	n/a	n/a	1.636	n/a	1.602	n/a	0.5097
5E+06	12	1	1	1	9.143	n/a	11.954	3.383	3.306	6.096	3.097	n/a	n/a	n/a	1.408	n/a	1.485	n/a	0.4653
5E+06	12	1	1	1	11.743	n/a	11.125	3.72	3.223	5.615	2.768	n/a	n/a	n/a	2.976	n/a	2.678	n/a	0.8174
5E+06	12	1	1	0	n/a	n/a	12.121	3.159	3.664	5.094	1.705	n/a	n/a	n/a	1.72	n/a	1.585	n/a	0.531
5E+06	12	1	1	1	10.427	n/a	8.537	3.211	2.571	4.022	1.764	n/a	n/a	n/a	0.889	n/a	1.258	n/a	0.214
5E+06	12	1	1	1	6.464	n/a	12.873	3.383	4.318	6.237	2.14	n/a	n/a	n/a	2.321	n/a	2.094	n/a	0.7271
5E+06	13	1	1	1	9.165	n/a	7.564	2.726	2.891	4.516	1.727	n/a	n/a	n/a	1.486	n/a	0.93	n/a	0.1515
5E+06	13	1	1	0	n/a	n/a	8.833	2.842	2.768	5.221	2.268	n/a	n/a	n/a	1.031	n/a	1.368	n/a	0.2639
5E+06	13	1	1	1	6.626	n/a	10.447	3.151	3.159	5.578	2.181	n/a	n/a	n/a	2.089	n/a	1.822	n/a	0.6356
5E+06	13	1	1	1	9.686	n/a	10.002	3.432	3.026	5.567	2.842	n/a	n/a	n/a	1.666	n/a	1.666	n/a	0.5433
5E+06	13	1	1	1	4.953	n/a	6.422	3.368	2.645	3.618	0.641	n/a	n/a	n/a	1.402	n/a	0.985	n/a	0.0495
5E+06	13	1	1	1	6.61	n/a	9.741	2.801	2.983	4.738	2.028	n/a	n/a	n/a	1.433	n/a	1.433	n/a	0.372
5E+06	13	1	1	1	5.76	n/a	10.801	3.306	2.842	5.388	3.383	n/a	n/a	n/a	1.363	n/a	1.909	n/a	0.5249
5E+06	13	1	1	1	11.161	n/a	10.976	3.657	3.271	4.716	1.828	n/a	n/a	n/a	3.39	n/a	2.035	n/a	0.8137
5E+06	13	1	1	1	6.068	n/a	11.619	2.891	2.688	4.561	2.448	n/a	n/a	n/a	2.172	n/a	1.85	n/a	0.6621
5E+06	13	1	1	1	6.805	n/a	10.787	3.29	2.801	4.586	2.443	n/a	n/a	n/a	1.223	n/a	1.5	n/a	0.3968
5E+06	14	2	2	2	10.922	5.582	6.171	4.805	2.085	3.402	1.322	2.586	4.098	1.252	1.726	1.08	0.812	1.002	0.9298
5E+06	14	1	1	2	9.19	1.612	11.435	3.348	2.926	5.539	2.948	n/a	n/a	n/a	2.332	n/a	2.155	n/a	0.7306
5E+06	14	1	1	1	10.984	n/a	10.268	3.657	2.268	4.209	2.157	n/a	n/a	n/a	2.706	n/a	1.889	n/a	0.7554
5E+06	14	1	1	1	6.372	n/a	5.757	3.243	2.731	3.961	1.814	n/a	n/a	n/a	1.188	n/a	1.1	n/a	0.0042
5E+06	14	1	1	2	5.873	1.828	10.233	4.019	2.801	4.38	1.793	n/a	n/a	n/a	2.602	n/a	2.147	n/a	0.8026
5E+06	14	1	1	1	13.174	n/a	13.394	2.085	3.175	5.61	3.618	n/a	n/a	n/a	1.006	n/a	1.305	n/a	0.4024
5E+06	14	1	1	1	9.234	n/a	10.943	3.064	3.462	5.194	2.245	n/a	n/a	n/a	1.836	n/a	1.554	n/a	0.5494
5E+06	14	1	1	1	6.103	n/a	10.108	3.862	2.768	5.124	2.837	n/a	n/a	n/a	1.946	n/a	1.868	n/a	0.6095
5E+06	14	1	1	1	5.403	n/a	6.612	3.409	2.944	4.209	1.134	n/a	n/a	n/a	1.29	n/a	1.182	n/a	0.0738
5E+06	14	1	1	1	11.463	n/a	9.483	3.211	2.996	4.653	1.635	n/a	n/a	n/a	1.414	n/a	1.354	n/a	0.3182
5E+06	14	1	1	1	8.093	n/a	11.601	3.306	3.026	5.557	3.114	n/a	n/a	n/a	1.796	n/a	1.847	n/a	0.5871
5E+06	14	1	1	1	5.938	n/a	6.456	2.842	2.926	3.961	1.076	n/a	n/a	n/a	1.217	n/a	1.081	n/a	0.0473
5E+06	14	1	1	2	11.921	1.167	11.398	3.306	3.013	5.578	2.842	n/a	n/a	n/a	2.284	n/a	2.386	n/a	0.7483
5E+06	14	1	1	1	11.235	n/a	11.075	3.247	3.409	5.221	2.151	n/a	n/a	n/a	2.268	n/a	2.34	n/a	0.7447
5E+06	14	1	1	1	5.933	n/a	7.921	3.043	2.886	4.009	1.014	n/a	n/a	n/a	1.313	n/a	1.143	n/a	0.18
5E+06	14	1	1	1	14.028	n/a	7.89	4.683	2.891	3.08	1.727	n/a	n/a	n/a	1.277	n/a	1.337	n/a	0.1872
5E+06	14	1	1	1	5.452	n/a	9.975	3.528	2.983	4.784	2.443	n/a	n/a	n/a	1.945	n/a	1.775	n/a	0.6031
5E+06	14	1	1	1	11.531	n/a	13.34	3.66	2.726	5.667	2.842	n/a	n/a	n/a	1.436	n/a	1.815	n/a	0.5128
5E+06	15	1	1	1	6.467	n/a	8.636	3.812	2.669	4.237	2.251	n/a	n/a	n/a	1.115	n/a	1.104	n/a	0.2312
5E+06	15	1	1	1	6.068	n/a	6.366	2.731	2.335	4.107	1.479	n/a	n/a	n/a	1.04	n/a	1.449	n/a	0.0451
5E+06	15	1	1	1	6.943	n/a	9.79	2.157	3.302	4.735	1.651	n/a	n/a	n/a	1.291	n/a	1.365	n/a	0.3315
5E+06	15	1	1	2	7.386	3.448	7.598	3.183	2.837	4.333	1.37	n/a	n/a	n/a	2.841	n/a	2.709	n/a	0.8552
5E+06	15	1	1	1	6.455	n/a	6.99	2.405	2.801	3.879	1.014	n/a	n/a	n/a	1.179	n/a	1.152	n/a	0.0962
5E+06	15	1	1	1	5.695	n/a	7.864	2.591	3.383	5.223	1.958	n/a	n/a	n/a	1.407	n/a	1.184	n/a	0.1824
5E+06	15	1	1	1	5.561	n/a	10.222	2.405	2.645	4.979	2.109	n/a	n/a	n/a	0.889	n/a	1.427	n/a	0.3448

5E+06	15	1	1	1	7.319	n/a	9.395	2.654	2.505	4.209	1.579	n/a	n/a	n/a	1.016	n/a	1.567	n/a	0.3077
5E+06	15	1	1	1	5.671	n/a	9.063	2.855	2.688	4.456	1.705	n/a	n/a	n/a	2.782	n/a	2.753	n/a	0.8362
5E+06	15	1	1	1	8.634	n/a	8.284	2.495	3.114	4.586	1.771	n/a	n/a	n/a	1.261	n/a	1.554	n/a	0.2362
5E+06	15	1	1	1	0.935	n/a	10.173	3.271	3.247	3.931	1.283	n/a	n/a	n/a	0.977	n/a	1.399	n/a	0.3422
5E+06	15	1	1	1	7.448	n/a	10.859	2.63	3.271	5.594	2.61	n/a	n/a	n/a	1.365	n/a	1.723	n/a	0.477
5E+06	15	1	2	2	15.671	4.887	8.334	4.877	3.159	4.047	1.513	n/a	4.356	2.181	2.651	n/a	1.675	1.643	0.8476
5E+06	15	1	1	1	7.046	n/a	11.898	2.61	2.74	5.933	2.745	n/a	n/a	n/a	2.165	n/a	2.585	n/a	0.759
5E+06	15	2	2	2	11.232	3.956	6.639	4.362	1.604	2.251	1.322	3.064	3.085	0.641	1.2	0.782	1.703	1.24	0.9019
5E+06	15	2	2	2	7.37	2.612	7.645	5.171	2.957	3.812	1.134	2.586	3.915	1.705	1.061	1.035	1.419	1.509	0.8706
5E+06	15	1	1	1	11.033	n/a	10.697	2.296	3.247	5.782	3.047	n/a	n/a	n/a	2.547	n/a	2.555	n/a	0.81
5E+06	15	1	1	1	12.583	n/a	10.979	2.61	2.944	4.848	2.384	n/a	n/a	n/a	1.934	n/a	2.328	n/a	0.6823
5E+06	15	1	1	1	4.988	n/a	7.072	3.114	2.566	3.774	1.37	n/a	n/a	n/a	1.049	n/a	1.338	n/a	0.103
5E+06	15	1	1	1	2.887	n/a	11.515	3.702	3.195	5.622	2.411	n/a	n/a	n/a	1.876	n/a	2.574	n/a	0.7236
5E+06	15	1	1	1	10.239	n/a	11.564	2.759	3.561	6.169	2.801	n/a	n/a	n/a	1.629	n/a	2.762	n/a	0.7132
5E+06	15	1	1	1	11.859	n/a	12.85	2.768	2.768	5.683	2.74	n/a	n/a	n/a	1.663	n/a	2.194	n/a	0.6421
5E+06	15	1	1	1	12.738	n/a	11.855	3.043	2.443	4.906	2.904	n/a	n/a	n/a	1.243	n/a	1.513	n/a	0.4392
5E+06	15	1	1	2	9.161	1.014	10.947	3.517	2.904	4.617	1.705	n/a	n/a	n/a	2.192	n/a	2.495	n/a	0.7626
5E+06	15	1	1	1	9.338	n/a	8.172	3.604	3.371	4.182	1.014	n/a	n/a	n/a	1.436	n/a	0.74	n/a	0.1848
5E+06	15	1	1	1	9.521	n/a	11.059	3.247	2.891	5.141	2.268	n/a	n/a	n/a	0.811	n/a	1.312	n/a	0.3638
5E+06	15	1	1	1	4.37	n/a	6.766	2.745	2.904	4.403	1.293	n/a	n/a	n/a	1.285	n/a	1.227	n/a	0.085
5E+06	15	1	1	1	7.661	n/a	7.022	2.591	2.296	3.702	1.521	n/a	n/a	n/a	1.171	n/a	1.389	n/a	0.1053
5E+06	15	1	1	1	4.922	n/a	8.856	2.268	2.405	4.047	2.091	n/a	n/a	n/a	1.223	n/a	1.357	n/a	0.2766
5E+06	16	1	1	1	4.869	n/a	6.99	2.251	2.586	3.812	1.814	n/a	n/a	n/a	0.851	n/a	1.74	n/a	0.1007
5E+06	16	1	1	1	5.567	n/a	9.293	2.268	2.996	5.063	2.427	n/a	n/a	n/a	1.412	n/a	1.456	n/a	0.313
5E+06	16	2	2	2	7.005	2.32	7.699	5.452	2.535	3.586	0.975	2.654	3.462	0.864	1.169	1.042	1.209	1.768	0.8667
5E+06	16	1	1	1	5.701	n/a	7.343	2.085	2.721	4.138	n/a	n/a	n/a	n/a	1.218	n/a	1.378	n/a	0.1305
5E+06	16	2	2	2	9.297	4.122	7.544	5.171	2.891	3.66	n/a	2.891	4.009	n/a	1.452	1.228	1.339	1.45	0.859
5E+06	16	1	1	1	11.817	n/a	10.458	3.306	2.495	4.377	2.085	n/a	n/a	n/a	1.762	n/a	1.679	n/a	0.5619
5E+06	16	2	2	2	8.12	3.621	6.807	5.176	2.855	4.16	1.579	3.043	3.812	1.027	1.079	1.15	1.474	1.44	0.894
5E+06	16	1	1	1	10.76	n/a	12.632	3.754	3.337	4.979	2.489	n/a	n/a	n/a	1.13	n/a	1.65	n/a	0.4623
5E+06	16	1	1	1	7.021	n/a	11.231	3.183	3.383	5.485	2.405	n/a	n/a	n/a	2.071	n/a	1.781	n/a	0.6323
5E+06	16	2	2	2	8.632	4.643	5.688	5.243	2.891	3.532	1.134	2.721	3.628	0.962	1.109	1.045	1.309	1.241	0.9258
5E+06	16	1	1	1	9.747	n/a	9.975	2.768	2.869	4.981	2.489	n/a	n/a	n/a	1.227	n/a	1.338	n/a	0.3368
5E+06	16	1	1	1	7.263	n/a	6.203	2.745	2.891	3.754	1.283	n/a	n/a	n/a	0.963	n/a	1.361	n/a	0.0213
5E+06	16	1	1	1	3.744	n/a	7.171	2.645	2.645	3.402	1.167	n/a	n/a	n/a	0.846	n/a	1.414	n/a	0.1121
5E+06	17	1	1	1	10.059	n/a	10.682	3.207	2.944	4.333	1.931	n/a	n/a	n/a	1.439	n/a	1.729	n/a	0.4948
5E+06	17	1	1	1	5.916	n/a	7.759	3.905	3.151	4.172	1.793	n/a	n/a	n/a	1.136	n/a	1.18	n/a	0.1681
5E+06	17	1	1	1	10.694	n/a	10.199	2.957	3.013	5.352	2.645	n/a	n/a	n/a	1.072	n/a	1.31	n/a	0.3475
5E+06	17	1	1	1	5.883	n/a	6.822	2.654	2.489	3.967	1.452	n/a	n/a	n/a	1.274	n/a	1.396	n/a	0.0917
5E+06	17	1	1	1	8.003	n/a	10.363	3.097	3.013	4.333	1.958	n/a	n/a	n/a	1.207	n/a	1.508	n/a	0.3885
5E+06	17	1	1	1	8.859	n/a	10.454	3.532	2.731	4.877	2.296	n/a	n/a	n/a	1.514	n/a	1.376	n/a	0.4249
5E+06	17	1	1	1	12.619	n/a	6.446	4.022	2.891	3.462	0.935	n/a	n/a	n/a	1.389	n/a	1.406	n/a	0.0671
5E+06	17	1	1	1	7.304	n/a	9.791	3.469	3.29	5.966	2.443	n/a	n/a	n/a	1.392	n/a	1.653	n/a	0.4306
5E+06	17	1	1	1	6.238	n/a	8.334	2.996	2.535	4.628	2.571	n/a	n/a	n/a	0.99	n/a	1.166	n/a	0.1945
5E+06	17	1	1	1	14.301	n/a	11.231	3.402	2.944	4.824	1.924	n/a	n/a	n/a	1.938	n/a	1.494	n/a	0.5556
5E+06	17	1	1	1	7.627	n/a	8.754	3.302	2.768	4.098	2.085	n/a	n/a	n/a	1.552	n/a	1.452	n/a	0.2869
5E+06	17	1	1	1	8.193	n/a	6.414	3.532	2.028	3.432	1.651	n/a	n/a	n/a	0.844	n/a	1.017	n/a	0.0278
5E+06	17	1	1	2	5.749	1.123	10.99	3.604	3.163	4.805	1.793	n/a	n/a	n/a	2.459	n/a	2.324	n/a	0.7806
5E+06	17	1	1	1	10.199	n/a	10.622	3.114	2.505	5.968	3.227	n/a	n/a	n/a	1.288	n/a	1.644	n/a	0.4363
5E+06	17	1	1	1	8.898	n/a	11.626	3.227	3.114	4.625	2.009	n/a	n/a	n/a	1.152	n/a	1.181	n/a	0.394
5E+06	17	1	1	1	5.083	n/a	6.749	2.855	3.532	4.234	1.027	n/a	n/a	n/a	1.246	n/a	0.811	n/a	0.0693
5E+06	17	1	1	1	4.022	n/a	6.592	3.532	2.74	3.931	1.134	n/a	n/a	n/a	1.492	n/a	1.152	n/a	0.0782
5E+06	17	1	1	1	14.99	n/a	11.462	2.63	3.175	5.837	2.51	n/a	n/a	n/a	1.352	n/a	1.492	n/a	0.445
5E+06	17	1	1	1	7.701	n/a	10.945	3.064	2.61	5.695	3.085	n/a	n/a	n/a	1.304	n/a	1.608	n/a	0.4421
5E+06	17	1	1	1	5.75	n/a	7.429	3.348	2.996	4.123	2.091	n/a	n/a	n/a	1.133	n/a	1.075	n/a	0.1259
5E+06	17	1	1	1	6.498	n/a	6.494	3.247	2.669	4.022	1.37	n/a	n/a	n/a	1.04	n/a	0.973	n/a	0.0364
5E+06	17	1	1	1	11.473	n/a	11.089	3.163	2.869	5.236	1.87	n/a	n/a	n/a	1.478	n/a	1.316	n/a	0.4221
5E+06	17	1	1	1	4.267	n/a	7.539	2.448	2.948	3.604	0.717	n/a	n/a	n/a	0.93	n/a	1.055	n/a	0.1282
5E+06	17	1	2	2	7.429	2.336	6.106	4.218	2.109	2.837	1.513	n/a	3.043	1.156	2.011	n/a	1.197	0.965	0.9501
5E+06	18	1	1	1	11.896	n/a	10.79	3.348	n/a	n/a	n/a	n/a	n/a	n/a	0.996	n/a	1.258	n/a	0.3611
5E+06	18	2	2	2	13.364	5.298	5.831	5.426	2.891	3.879	0.661	3.306	3.013	0.661	1.01	0.712	1.436	1.053	0.946
5E+06	18	2	2	1	5.18	n/a	8.621	3.064	2.591	4.197	1.521	n/a	n/a	n/a	1.318	1.278	1.421	1.29	0.8438
5E+06	18	1	1	1	7.5	n/a	7.948	2.886	2.731	4.473	2.085	n/a	n/a	n/a	1.5	n/a	1.413	n/a	0.2066
5E+06	18	1	1	1	6.319	n/a	8.692	2.869	2.842	4.595	2.157	n/a	n/a	n/a	0.92	n/a	1.146	n/a	0.2238
5E+06	18	1	1	1	13.009	n/a	10.803	3.223	2.61	4.493	2.591	n/a	n/a	n/a	1.92	n/a	1.458	n/a	0.5463

5E+06	18	1	1	2	7.349	1.134	11.61	3.55	3.085	5.631	2.731	n/a	n/a	n/a	2.337	n/a	2.156	n/a	0.7341
5E+06	18	1	1	1	4.199	n/a	6.194	2.74	2.489	3.207	1.293	n/a	n/a	n/a	0.719	n/a	1.297	n/a	0.0149
5E+06	18	1	1	1	14.101	n/a	8.194	3.29	2.505	4.261	2.61	n/a	n/a	n/a	1.047	n/a	1.44	n/a	0.2042
5E+06	18	1	1	1	3.693	n/a	10.905	3.085	2.944	4.94	2.586	n/a	n/a	n/a	2.372	n/a	2.417	n/a	0.7879
5E+06	18	1	1	1	11.46	n/a	10.076	2.983	2.157	5.094	2.335	n/a	n/a	n/a	1.61	n/a	1.335	n/a	0.4193
5E+06	18	1	2	2	12.762	3.466	7.89	4.716	2.405	3.657	1.735	n/a	3.875	2.384	2.356	n/a	1.191	1.257	0.8822
5E+06	18	1	1	1	7.944	n/a	8.297	2.837	3.163	5.134	2.279	n/a	n/a	n/a	1.098	n/a	1.176	n/a	0.1993
5E+06	18	1	1	1	8.893	n/a	11.697	2.805	3.247	5.748	2.996	n/a	n/a	n/a	2.316	n/a	1.982	n/a	0.7063
5E+06	18	1	1	1	11.664	n/a	12.591	3.785	2.61	5.253	2.181	n/a	n/a	n/a	1.717	n/a	1.39	n/a	0.4799
5E+06	18	1	1	1	9.274	n/a	11.987	3.398	3.306	5.775	3.371	n/a	n/a	n/a	2.339	n/a	2.086	n/a	0.7201
5E+06	18	1	1	1	10.723	n/a	7.13	2.405	2.688	3.55	1.167	n/a	n/a	n/a	1.318	n/a	1.034	n/a	0.1098
5E+06	18	1	1	1	7.102	n/a	8.878	3.302	2.535	4.501	1.87	n/a	n/a	n/a	1.381	n/a	1.068	n/a	0.269
5E+06	18	1	1	1	14.456	n/a	11.202	3.812	2.489	6.24	3.825	n/a	n/a	n/a	1.176	n/a	1.112	n/a	0.3775
5E+06	18	1	1	1	9.948	n/a	11.833	3.586	3.085	5.379	2.505	n/a	n/a	n/a	1.486	n/a	1.779	n/a	0.5188
5E+06	18	1	1	1	15.78	n/a	12.322	3.757	2.61	5.338	2.983	n/a	n/a	n/a	1.434	n/a	1.678	n/a	0.4858
5E+06	18	1	1	1	5.812	n/a	6.897	2.405	3.114	3.879	1.587	n/a	n/a	n/a	0.922	n/a	0.989	n/a	0.0805
5E+06	18	1	1	1	11.242	n/a	7.207	2.091	2.411	3.604	1.379	n/a	n/a	n/a	1.136	n/a	1.046	n/a	0.1076
1E+07	1	1	1	1	6.685	n/a	7.347	2.029	2.27	3.504	1.673	n/a	n/a	n/a	0.98	n/a	1.12	n/a	0.1346
1E+07	1	1	1	1	6.981	n/a	7.596	2.441	2.438	4.579	2.358	n/a	n/a	n/a	1.097	n/a	1.159	n/a	0.1545
1E+07	1	1	1	1	6.827	n/a	9.058	2.073	2.214	4.057	1.894	n/a	n/a	n/a	1.454	n/a	1.117	n/a	0.2803
1E+07	1	1	1	1	8.69	n/a	10.579	2.491	2.313	3.629	1.55	n/a	n/a	n/a	0.615	n/a	0.997	n/a	0.3446
1E+07	1	1	1	1	5.481	n/a	7.309	2.313	2.441	3.895	1.823	n/a	n/a	n/a	0.66	n/a	1.169	n/a	0.1125
1E+07	1	1	1	1	6.192	n/a	7.309	2.313	1.894	3.278	1.462	n/a	n/a	n/a	0.802	n/a	1.137	n/a	0.1149
1E+07	1	1	1	1	6.075	n/a	6.693	2.434	2.029	3.814	1.787	n/a	n/a	n/a	0.796	n/a	1.077	n/a	0.0764
1E+07	2	1	1	2	9.29	0.309	13.533	2.678	2.434	5.745	3.39	n/a	n/a	n/a	1.18	n/a	1.407	n/a	0.4922
1E+07	2	1	1	1	8.778	n/a	11.349	2.274	2.566	4.163	1.607	n/a	n/a	n/a	2.007	n/a	1.773	n/a	0.6389
1E+07	2	1	1	1	6.318	n/a	8.43	1.814	2.073	2.441	2.441	n/a	n/a	n/a	0.517	n/a	1.075	n/a	0.1696
1E+07	2	1	1	1	11.8	n/a	12.396	2.995	2.181	5.824	3.827	n/a	n/a	n/a	1.413	n/a	1.751	n/a	0.5308
1E+07	2	1	1	1	5.829	n/a	6.098	2.578	2.366	3.367	1.339	n/a	n/a	n/a	0.829	n/a	0.977	n/a	0.0319
1E+07	2	1	1	1	9.407	n/a	11.594	2.617	1.545	3.21	1.942	n/a	n/a	n/a	1.964	n/a	1.901	n/a	0.6495
1E+07	2	1	1	1	9.799	n/a	12.03	2.976	2.196	3.755	1.711	n/a	n/a	n/a	1.876	n/a	1.908	n/a	0.646
1E+07	3	2	2	2	8.281	5.558	6.778	4.649	2.464	2.817	1.147	2.451	4.01	1.673	0.755	0.677	0.958	1.051	1
1E+07	3	1	1	1	13.691	n/a	7.777	2.53	2.718	3.917	1.496	n/a	n/a	n/a	0.878	n/a	1.028	n/a	0.157
1E+07	4	1	1	1	9.078	n/a	11.578	2.869	2.594	5.062	2.468	n/a	n/a	n/a	2.359	n/a	2.402	n/a	0.775
1E+07	5	1	1	1	8.91	n/a	8.557	2.578	2.642	3.983	1.687	n/a	n/a	n/a	1.145	n/a	1.37	n/a	0.2345
1E+07	5	1	1	1	4.256	n/a	7.934	2.746	2.069	4.131	2.073	n/a	n/a	n/a	0.536	n/a	0.987	n/a	0.152
1E+07	5	1	1	1	4.946	n/a	11.571	2.434	2.451	4.67	2.41	n/a	n/a	n/a	0.91	n/a	1.35	n/a	0.4393
1E+07	5	1	1	1	12.078	n/a	10.737	2.434	2.341	4.026	1.823	n/a	n/a	n/a	1.144	n/a	0.982	n/a	0.3971
1E+07	5	1	1	1	11.082	n/a	12.475	2.849	2.645	4.952	2.366	n/a	n/a	n/a	0.909	n/a	1.337	n/a	0.4671
1E+07	5	1	1	1	10.047	n/a	10.293	2.464	2.323	4.392	2.192	n/a	n/a	n/a	1.917	n/a	1.863	n/a	0.625
1E+07	5	1	1	1	11.072	n/a	14.126	3.631	2.451	4.362	2.214	n/a	n/a	n/a	1.576	n/a	1.442	n/a	0.5049
1E+07	5	1	1	1	15.464	n/a	11.646	2.645	2.421	5.088	2.67	n/a	n/a	n/a	1.915	n/a	1.754	n/a	0.6043
1E+07	5	1	1	1	12.22	n/a	11.141	2.582	3.103	4.877	1.962	n/a	n/a	n/a	1.686	n/a	1.884	n/a	0.5872
1E+07	5	1	1	1	8.646	n/a	8.858	3.207	3.05	4.448	1.566	n/a	n/a	n/a	0.942	n/a	1.849	n/a	0.2694
1E+07	6	2	2	2	12.922	6.049	6.391	4.099	1.796	2.894	1.217	2.029	3.207	1.182	0.851	1.108	1.249	1.042	0.9955
1E+07	6	1	1	1	13.178	n/a	13.691	2.241	2.241	4.89	2.822	n/a	n/a	n/a	1.863	n/a	1.772	n/a	0.5805
1E+07	6	1	1	1	15.097	n/a	10.138	1.726	2.358	4.177	2.295	n/a	n/a	n/a	0.657	n/a	0.971	n/a	0.3023
1E+07	6	1	1	1	7.334	n/a	8.903	2.181	2.192	4.057	1.687	n/a	n/a	n/a	0.913	n/a	1.072	n/a	0.2398
1E+07	6	1	1	1	11.047	n/a	8.665	2.386	2.284	4.773	2.645	n/a	n/a	n/a	0.934	n/a	1.08	n/a	0.216
1E+07	6	1	1	1	14.33	n/a	8.314	2.386	2.088	4.249	2.358	n/a	n/a	n/a	1.109	n/a	1.338	n/a	0.2108
1E+07	6	1	1	1	8.813	n/a	11.864	2.366	2.421	3.674	1.308	n/a	n/a	n/a	1.12	n/a	1.367	n/a	0.4639
1E+07	6	1	1	1	6.999	n/a	10.307	2.185	2.241	3.902	1.85	n/a	n/a	n/a	0.904	n/a	1.142	n/a	0.3504
1E+07	6	1	1	1	8.302	n/a	8.662	1.837	2.116	3.346	1.711	n/a	n/a	n/a	1.094	n/a	1.014	n/a	0.2239
1E+07	6	1	1	1	8.128	n/a	12.048	2.727	2.746	4.65	2.366	n/a	n/a	n/a	1.529	n/a	1.552	n/a	0.5146
1E+07	6	1	1	1	14.444	n/a	10.045	2.341	2.214	3.594	1.745	n/a	n/a	n/a	0.907	n/a	1.168	n/a	0.3332
1E+07	6	1	1	1	6.598	n/a	8.356	2.069	2.323	3.53	1.496	n/a	n/a	n/a	0.93	n/a	1.342	n/a	0.2056
1E+07	6	1	1	1	7.474	n/a	10.524	2.678	2.438	3.85	1.545	n/a	n/a	n/a	1.118	n/a	1.224	n/a	0.3942
1E+07	6	1	1	1	7.804	n/a	9.626	2.468	2.341	3.27	1.089	n/a	n/a	n/a	0.869	n/a	1.493	n/a	0.3163
1E+07	6	1	1	2	9.326	0.258	10.654	2.814	2.566	4.775	2.478	n/a	n/a	n/a	2.047	n/a	2.262	n/a	0.7109
1E+07	7	1	1	1	8.365	n/a	7.579	0.807	2.578	3.522	0.977	n/a	n/a	n/a	0.608	n/a	0.924	n/a	0.1296
1E+07	7	1	1	1	8.048	n/a	9.149	2.004	2.27	3.902	2.313	n/a	n/a	n/a	1.092	n/a	0.935	n/a	0.2531
1E+07	7	1	1	1	6.705	n/a	9.866	2.491	2.468	4.137	1.545	n/a	n/a	n/a	1.047	n/a	1.253	n/a	0.3389
1E+07	7	1	1	1	10.327	n/a	11.644	2.313	2.448	5.39	3.122	n/a	n/a	n/a	0.75	n/a	0.988	n/a	0.3912
1E+07	7	1	1	1	6.542	n/a	6.908	2.069	2.358	3.79	1.321	n/a	n/a	n/a	1.119	n/a	0.996	n/a	0.0979
1E+07	8	1	1	1	13.29	n/a	8.955	2.309	2.826	5.773	2.834	n/a	n/a	n/a	1.56	n/a	1.844	n/a	0.5737

1E+07	8	1	1	1	13.622	n/a	11.894	2.491	2.323	4.388	2.123	n/a	n/a	n/a	1.447	n/a	1.86	n/a	0.5471
1E+07	9	1	1	1	8.169	n/a	10.612	1.929	2.578	4.282	1.587	n/a	n/a	n/a	1.173	n/a	1.47	n/a	0.4332
1E+07	9	1	1	1	6.517	n/a	7.055	1.434	2.004	2.746	0.748	n/a	n/a	n/a	0.647	n/a	1.107	n/a	0.0955
1E+07	9	1	1	1	5.543	n/a	9.065	2.185	2.323	4.251	1.837	n/a	n/a	n/a	0.752	n/a	1.14	n/a	0.2478
1E+07	9	1	1	1	13.528	n/a	11.424	2.826	2.15	4.512	2.366	n/a	n/a	n/a	2.019	n/a	1.915	n/a	0.6637
1E+07	9	1	1	1	20.834	n/a	11.628	2.642	2.645	5.29	2.491	n/a	n/a	n/a	1.102	n/a	1.656	n/a	0.4827
1E+07	9	1	1	1	10.022	n/a	6.71	2.214	1.962	3.895	1.954	n/a	n/a	n/a	0.983	n/a	1.167	n/a	0.0811
1E+07	10	1	1	1	7.675	n/a	12.284	2.451	2.478	4.997	2.775	n/a	n/a	n/a	1.07	n/a	1.495	n/a	0.4764
1E+07	11	1	1	1	12.89	n/a	13.034	2.566	2.645	5.285	3.335	n/a	n/a	n/a	2.7	n/a	2.045	n/a	0.7597
1E+07	11	1	1	1	12.522	n/a	11.772	1.382	2.309	4.902	2.569	n/a	n/a	n/a	1.729	n/a	1.297	n/a	0.5081
1E+07	11	1	1	1	4.983	n/a	6.563	2.451	1.796	3.613	1.712	n/a	n/a	n/a	2.998	n/a	2.098	n/a	0.9168
1E+07	11	1	1	1	11.411	n/a	12.596	2.569	3.082	5.044	2.67	n/a	n/a	n/a	2.734	n/a	2.238	n/a	0.7867
1E+07	11	1	1	1	10.745	n/a	6.852	2.925	2.069	3.387	1.668	n/a	n/a	n/a	1.896	n/a	1.373	n/a	0.1371
1E+07	11	1	1	2	14.863	3.085	8.814	3.338	3.49	5.576	2.645	n/a	n/a	n/a	3.318	n/a	2.659	n/a	0.8625
1E+07	11	1	1	1	11.72	n/a	7.757	2.775	2.491	3.851	1.308	n/a	n/a	n/a	1.704	n/a	1.409	n/a	0.1978
1E+07	11	1	1	1	9.76	n/a	10.71	2.437	2.848	4.618	2.962	n/a	n/a	n/a	1.781	n/a	1.595	n/a	0.5571
1E+07	11	1	1	1	16.306	n/a	11.375	2.192	2.775	4.648	3.1	n/a	n/a	n/a	2.655	n/a	1.917	n/a	0.7445
1E+07	11	1	1	1	10.23	n/a	11.474	2.579	2.769	4.618	2.743	n/a	n/a	n/a	2.394	n/a	2.387	n/a	0.7789
1E+07	11	1	1	1	7.546	n/a	6.031	2.67	2.313	3.985	1.434	n/a	n/a	n/a	1.411	n/a	1.524	n/a	0.0575
1E+07	11	1	1	1	6.012	n/a	5.936	3.09	1.941	2.769	1.566	n/a	n/a	n/a	2.061	n/a	1.916	n/a	0.9296
1E+07	11	1	1	1	10.004	n/a	5.92	2.491	1.721	2.848	0.811	n/a	n/a	n/a	1.519	n/a	1.422	n/a	0.0435
1E+07	11	1	1	1	8.16	n/a	9.246	2.365	2.883	4.599	2.27	n/a	n/a	n/a	1.754	n/a	1.628	n/a	0.567
1E+07	11	1	1	1	4.868	n/a	6.605	2.028	3.09	3.859	1.411	n/a	n/a	n/a	1.672	n/a	1.164	n/a	0.1003
1E+07	12	1	1	1	12.43	n/a	11.489	2.478	2.822	5.414	2.582	n/a	n/a	n/a	1.67	n/a	1.287	n/a	0.4954
1E+07	12	1	1	1	6.273	n/a	7.192	2.814	2.185	3.857	1.155	n/a	n/a	n/a	2.12	n/a	1.363	n/a	0.9382
1E+07	12	1	1	1	8.001	n/a	11.363	2.925	2.309	3.548	1.434	n/a	n/a	n/a	2.004	n/a	1.877	n/a	0.653
1E+07	12	1	1	1	12.152	n/a	9.269	2.053	2.088	5.131	3.756	n/a	n/a	n/a	2.097	n/a	2.667	n/a	0.8707
1E+07	12	1	1	1	8.082	n/a	7.892	2.27	2.284	3.808	1.712	n/a	n/a	n/a	1.428	n/a	1.376	n/a	0.1875
1E+07	12	1	1	1	8.444	n/a	10.93	2.491	2.697	4.774	1.668	n/a	n/a	n/a	1.896	n/a	2.092	n/a	0.6673
1E+07	12	1	1	1	9.1	n/a	9.724	2.088	1.958	5.01	2.883	n/a	n/a	n/a	4.321	n/a	2.807	n/a	0.8301
1E+07	12	1	1	1	11.639	n/a	6.97	2.721	1.941	4.682	2.116	n/a	n/a	n/a	1.736	n/a	1.556	n/a	0.142
1E+07	12	1	1	1	6.583	n/a	8.614	2.284	1.929	2.769	1.034	n/a	n/a	n/a	0.912	n/a	0.828	n/a	0.1849
1E+07	12	2	2	2	8.897	1.965	6.986	3.747	2.632	3.791	0.77	2.359	3.423	1.026	1.734	1.484	1.422	1.593	0.9041
1E+07	12	1	1	1	12.849	n/a	10.982	2.579	2.579	3.53	1.643	n/a	n/a	n/a	1.221	n/a	1.235	n/a	0.4271
1E+07	12	1	1	1	8.299	n/a	7.724	2.053	2.196	3.925	1.566	n/a	n/a	n/a	1.686	n/a	1.592	n/a	0.2082
1E+07	12	1	1	1	4.97	n/a	5.478	2.718	2.323	3.594	1.155	n/a	n/a	n/a	2.034	n/a	1.733	n/a	0.9339
1E+07	12	1	1	1	8.656	n/a	12.496	2.313	2.527	4.362	1.814	n/a	n/a	n/a	2.524	n/a	2.557	n/a	0.7905
1E+07	13	2	2	1	5.3	n/a	9.197	3.278	2.825	4.131	2.004	n/a	n/a	n/a	2.433	2.452	2.088	1.912	0.8381
1E+07	13	1	1	1	6.922	n/a	10.079	2.54	2.775	5.01	2.816	n/a	n/a	n/a	2.372	n/a	2.586	n/a	0.8102
1E+07	13	1	1	1	2.717	n/a	6.474	1.814	2.814	3.084	1.264	n/a	n/a	n/a	1.691	n/a	1.3	n/a	0.0907
1E+07	13	1	1	1	2.805	n/a	5.336	3.238	2.069	2.825	1.814	n/a	n/a	n/a	1.179	n/a	1.429	n/a	0.0045
1E+07	13	1	1	1	5.772	n/a	6.426	2.069	n/a	2.323	3.473	n/a	n/a	n/a	1.424	n/a	1.347	n/a	0.0788
1E+07	13	1	1	1	7.458	n/a	9.794	2.995	2.273	3.851	1.954	n/a	n/a	n/a	1.508	n/a	1.55	n/a	0.489
1E+07	13	1	1	1	5.896	n/a	8.862	2.313	2.421	3.674	2.124	n/a	n/a	n/a	1.14	n/a	1.147	n/a	0.2505
1E+07	13	1	1	1	7.46	n/a	6.29	2.365	1.954	3.775	1.539	n/a	n/a	n/a	1.688	n/a	1.703	n/a	0.9469
1E+07	13	1	1	1	12.481	n/a	9.714	2.185	2.556	4.232	2.594	n/a	n/a	n/a	1.527	n/a	1.532	n/a	0.4859
1E+07	13	1	1	1	7.428	n/a	12.3	1.941	2.706	5.171	3.773	n/a	n/a	n/a	1.807	n/a	1.454	n/a	0.5406
1E+07	13	1	1	1	15.249	n/a	10.984	3.105	3.747	6.265	3.358	n/a	n/a	n/a	4.174	n/a	2.396	n/a	0.8221
1E+07	13	1	1	1	8.272	n/a	11.573	2.848	3.103	4.177	2.579	n/a	n/a	n/a	2.369	n/a	2.256	n/a	0.7559
1E+07	13	1	1	1	10.939	n/a	11.06	2.706	2.546	4.599	1.687	n/a	n/a	n/a	2.341	n/a	2.089	n/a	0.722
1E+07	13	1	1	1	9.241	n/a	11.133	2.441	2.468	4.705	2.072	n/a	n/a	n/a	1.884	n/a	1.919	n/a	0.6424
1E+07	13	1	1	1	8.858	n/a	9.778	1.339	2.679	3.859	1.668	n/a	n/a	n/a	1.183	n/a	0.982	n/a	0.3079
1E+07	13	2	2	2	13.849	8.724	10.264	2.556	2.53	4.982	2.566	2.814	4.568	2.135	1.558	3.389	1.58	2.62	0.8341
1E+07	13	1	1	1	13.084	n/a	9.914	2.645	2.598	5.127	2.953	n/a	n/a	n/a	1.623	n/a	1.612	n/a	0.5373
1E+07	13	1	1	1	9.197	n/a	10.593	2.763	2.869	4.841	2.004	n/a	n/a	n/a	2.963	n/a	2.696	n/a	0.8181
1E+07	13	1	1	1	8.602	n/a	9.207	2.313	2.192	3.49	2.057	n/a	n/a	n/a	1.579	n/a	1.221	n/a	0.3191
1E+07	13	1	1	1	2.174	n/a	8.521	2.706	2.196	4.364	1.907	n/a	n/a	n/a	1.897	n/a	1.557	n/a	0.5838
1E+07	13	1	1	1	8.697	n/a	12.424	2.769	2.617	4.453	2.365	n/a	n/a	n/a	2.212	n/a	1.97	n/a	0.7072
1E+07	13	1	1	0	n/a	n/a	12.438	2.869	2.697	3.859	1.85	n/a	n/a	n/a	2.402	n/a	2.057	n/a	0.737
1E+07	13	1	2	2	6.923	2.559	7.511	3.049	3.23	6.009	1.264	n/a	3.215	2.359	3.486	n/a	1.066	1.335	0.8957
1E+07	13	1	1	1	11.999	n/a	7.775	2.903	2.135	3.49	1.502	n/a	n/a	n/a	1.569	n/a	1.47	n/a	0.1901
1E+07	13	1	1	1	12.881	n/a	5.405	1.561	2.192	2.869	1.147	n/a	n/a	n/a	0.978	n/a	0.965	n/a	0.0023
1E+07	13	1	1	1	7.53	n/a	9.032	2.309	2.24	3.674	1.623	n/a	n/a	n/a	1.316	n/a	1.022	n/a	0.2612
1E+07	13	1	2	2	9.132	1.549	9.272	3.335	2.953	4.67	2.556	n/a	3.827	1.382	3.039	n/a	1.315	1.237	0.8543
1E+07	13	1	1	1	11.071	n/a	7.088	2.185	2.295	3.859	1.796	n/a	n/a	n/a	1.373	n/a	1.298	n/a	0.1321

1E+07	13	1	1	1	14.716	n/a	10.741	2.642	2.397	4.137	1.868	n/a	n/a	n/a	1.158	n/a	1.202	n/a	0.409
1E+07	13	1	2	2	6.735	3.596	6.046	4.057	1.962	3.669	1.388	n/a	3.019	1.566	1.544	n/a	1.57	1.289	0.9425
1E+07	13	1	1	0	n/a	n/a	11.281	1.962	3.729	5.133	1.801	n/a	n/a	n/a	2.949	n/a	1.832	n/a	0.7828
1E+07	13	1	1	1	1.814	n/a	9.309	2.491	2.67	5.236	2.359	n/a	n/a	n/a	1.011	n/a	0.957	n/a	0.2666
1E+07	13	1	1	1	5.45	n/a	6.563	2.185	2.185	2.822	0.529	n/a	n/a	n/a	1.645	n/a	1.02	n/a	0.0859
1E+07	13	1	1	1	4.341	n/a	6.291	2.295	2.645	3.387	0.925	n/a	n/a	n/a	1.587	n/a	0.93	n/a	0.0693
1E+07	13	1	1	1	4.949	n/a	8.519	2.24	3.103	4.512	1.958	n/a	n/a	n/a	2.735	n/a	2.339	n/a	0.8915
1E+07	13	2	2	2	10.999	4.012	6.27	4.475	2.192	2.951	1.034	1.782	3.006	1.183	2.426	1.52	1.147	1.38	0.9083
1E+07	13	1	1	1	9.181	n/a	11.58	2.745	2.341	3.859	1.823	n/a	n/a	n/a	3.058	n/a	2.47	n/a	0.7945
1E+07	13	1	1	2	8.625	3.925	8.363	3.96	2.67	5.209	2.53	n/a	n/a	n/a	2.425	n/a	1.975	n/a	0.9253
1E+07	13	1	1	1	20.962	n/a	10.866	2.192	2.775	5.03	2.181	n/a	n/a	n/a	1.775	n/a	1.963	n/a	0.618
1E+07	13	1	1	1	8.557	n/a	9.641	3.1	3.502	5.766	2.192	n/a	n/a	n/a	3.256	n/a	1.998	n/a	0.8462
1E+07	14	1	1	1	16.161	n/a	8.71	3.049	2.825	4.618	2.196	n/a	n/a	n/a	2.968	n/a	2.408	n/a	0.8748
1E+07	14	1	1	1	9.42	n/a	11.102	2.004	2.295	4.836	2.341	n/a	n/a	n/a	2.137	n/a	2.008	n/a	0.6926
1E+07	14	1	1	1	10.05	n/a	11.632	3.082	2.697	5.443	2.679	n/a	n/a	n/a	2.826	n/a	2.589	n/a	0.7984
1E+07	14	1	1	1	11.938	n/a	12.87	2.869	3.105	4.79	2.309	n/a	n/a	n/a	1.82	n/a	2.788	n/a	0.7521
1E+07	14	1	1	1	6.207	n/a	6.042	2.491	2.569	3.628	1.183	n/a	n/a	n/a	1.624	n/a	1.144	n/a	0.0505
1E+07	14	1	1	2	8.329	2.721	8.055	4.202	3.574	5.289	2.069	n/a	n/a	n/a	3.525	n/a	2.789	n/a	0.879
1E+07	14	1	2	2	8.597	4.432	6.127	4.22	2.313	4.369	1.954	n/a	4.026	1.463	4.106	n/a	1.457	1.632	0.8873
1E+07	14	1	1	1	9.362	n/a	10.495	1.745	2.706	4.774	2.15	n/a	n/a	n/a	3.035	n/a	2.441	n/a	0.8141
1E+07	14	1	1	1	7.56	n/a	7.631	2.196	2.397	4.748	2.434	n/a	n/a	n/a	1.487	n/a	1.197	n/a	0.1646
1E+07	14	1	1	1	9.313	n/a	10.319	2.579	2.769	4.131	2.27	n/a	n/a	n/a	1.558	n/a	1.796	n/a	0.5537
1E+07	14	1	1	1	6.552	n/a	7.954	3.079	1.907	3.631	1.283	n/a	n/a	n/a	1.269	n/a	1.813	n/a	0.2186
1E+07	14	1	1	1	11.621	n/a	10.838	2.903	2.883	4.539	1.868	n/a	n/a	n/a	3.269	n/a	2.427	n/a	0.8062
1E+07	14	1	1	1	14.622	n/a	7.982	2.883	2.313	3.567	1.85	n/a	n/a	n/a	1.354	n/a	1.425	n/a	0.2004
1E+07	14	1	1	1	13.899	n/a	7.966	2.491	2.527	3.215	0.898	n/a	n/a	n/a	1.134	n/a	1.648	n/a	0.1952
1E+07	14	1	1	1	8.885	n/a	8.664	2.124	2.598	4.584	1.837	n/a	n/a	n/a	1.2	n/a	1.741	n/a	0.2585
1E+07	14	1	1	1	12.83	n/a	10.511	2.931	2.365	4.626	1.907	n/a	n/a	n/a	3.87	n/a	2.523	n/a	0.8261
1E+07	14	1	1	1	8.253	n/a	12.516	2.421	2.181	4.556	2.763	n/a	n/a	n/a	2.287	n/a	1.901	n/a	0.7146
1E+07	14	1	1	1	7.906	n/a	11.397	2.721	2.903	5.414	2.53	n/a	n/a	n/a	2.29	n/a	1.917	n/a	0.6962
1E+07	14	1	1	1	7.066	n/a	6.17	2.834	2.569	3.49	1.27	n/a	n/a	n/a	1.488	n/a	1.269	n/a	0.0645
1E+07	14	1	1	1	7.858	n/a	10.67	2.491	1.587	3.827	2.053	n/a	n/a	n/a	1.608	n/a	1.568	n/a	0.534
1E+07	14	1	1	1	13.249	n/a	11.06	1.668	2.057	4.107	1.668	n/a	n/a	n/a	1.391	n/a	1.693	n/a	0.5178
1E+07	14	1	1	1	5.281	n/a	11.093	2.313	2.323	5.041	3.207	n/a	n/a	n/a	1.325	n/a	1.811	n/a	0.521
1E+07	14	1	1	1	6.453	n/a	5.289	2.295	2.057	2.697	1.264	n/a	n/a	n/a	1.434	n/a	1.281	n/a	0.0068
1E+07	14	1	1	2	10.783	3.19	9.018	2.434	2.67	4.249	1.364	n/a	n/a	n/a	3.301	n/a	2.278	n/a	0.8666
1E+07	14	1	1	1	10.432	n/a	11.538	2.273	2.323	4.647	2.951	n/a	n/a	n/a	1.321	n/a	1.112	n/a	0.4485
1E+07	15	1	1	1	9.08	n/a	5.886	2.434	2.848	2.825	0.654	n/a	n/a	n/a	3.138	n/a	0.846	n/a	0.9688
1E+07	15	1	1	1	11.084	n/a	6.487	1.924	2.15	2.721	1.155	n/a	n/a	n/a	1.407	n/a	1.379	n/a	0.0835
1E+07	15	1	1	1	5.127	n/a	6.734	2.743	2.355	3.103	1.587	n/a	n/a	n/a	2.511	n/a	1.435	n/a	0.9644
1E+07	15	1	1	1	13.168	n/a	9.662	2.84	2.52	4.22	2.116	n/a	n/a	n/a	3.444	n/a	2.926	n/a	0.8421
1E+07	15	1	2	2	8.156	2.397	7.402	4.59	2.642	4.27	2.028	n/a	4.051	1.712	3.247	n/a	1.232	1.712	0.8831
1E+07	15	1	1	1	6.455	n/a	5.681	2.207	2.566	2.931	1.183	n/a	n/a	n/a	1.496	n/a	1.639	n/a	0.0365
1E+07	15	1	1	1	5.243	n/a	9.219	2.491	2.931	5.195	3.145	n/a	n/a	n/a	3.351	n/a	2.119	n/a	0.8584
1E+07	15	1	1	1	5.808	n/a	6.148	2.355	2.962	3.7	1.463	n/a	n/a	n/a	1.791	n/a	1.613	n/a	0.96
1E+07	15	1	1	1	6.584	n/a	9.101	2.598	1.996	3.452	1.283	n/a	n/a	n/a	1.562	n/a	1.527	n/a	0.3418
1E+07	15	1	1	1	11.297	n/a	6.443	2.421	1.868	3.409	1.382	n/a	n/a	n/a	2.44	n/a	1.499	n/a	0.9556
1E+07	15	1	1	1	3.31	n/a	4.902	2.451	2.569	2.962	1.587	n/a	n/a	n/a	1.166	n/a	1.464	n/a	0.0113
1E+07	15	1	1	1	8.584	n/a	6.829	2.441	1.837	3.464	1.929	n/a	n/a	n/a	1.831	n/a	1.957	n/a	0.9512
1E+07	15	1	1	1	9.32	n/a	12.092	2.468	2.617	4.27	1.924	n/a	n/a	n/a	2.466	n/a	2.334	n/a	0.7712
1E+07	15	1	1	1	5.041	n/a	5.26	2.718	2.727	3.215	0.925	n/a	n/a	n/a	1.079	n/a	0.935	n/a	0.009
1E+07	15	1	1	1	4.99	n/a	6.634	2.088	2.451	3.1	1.026	n/a	n/a	n/a	1.826	n/a	1.318	n/a	0.11
1E+07	15	1	1	1	12.003	n/a	8.988	2.822	2.323	3.485	1.924	n/a	n/a	n/a	2.774	n/a	3.445	n/a	0.8503
1E+07	15	2	2	2	9.777	4.369	5.835	3.7	3.003	3.674	1.587	2.642	3.851	1.55	1.694	1.114	1.243	1.55	0.9125
1E+07	15	1	1	1	10.239	n/a	13.12	2.295	2.527	4.424	2.088	n/a	n/a	n/a	2.274	n/a	2.156	n/a	0.7332
1E+07	15	1	1	1	10.648	n/a	11.87	2.706	2.569	5.385	2.925	n/a	n/a	n/a	2.095	n/a	1.865	n/a	0.6709
1E+07	15	1	1	1	12.994	n/a	10.545	2.566	2.598	4.27	2.617	n/a	n/a	n/a	2.357	n/a	2.859	n/a	0.8023
1E+07	15	1	1	1	9.838	n/a	8.262	1.958	1.85	4.057	1.726	n/a	n/a	n/a	1.499	n/a	1.563	n/a	0.2371
1E+07	16	1	1	1	5.395	n/a	10.016	2.069	2.826	3.736	1.283	n/a	n/a	n/a	0.863	n/a	0.952	n/a	0.2996
1E+07	16	1	1	1	6.117	n/a	9.334	2.177	2.464	4.902	2.313	n/a	n/a	n/a	0.789	n/a	1.114	n/a	0.2639
1E+07	16	1	1	1	13.782	n/a	11.658	1.787	2.617	3.983	1.434	n/a	n/a	n/a	0.833	n/a	1.131	n/a	0.415
1E+07	16	1	1	1	7.923	n/a	9.71	2.029	2.15	3.808	1.907	n/a	n/a	n/a	0.673	n/a	0.848	n/a	0.2858
1E+07	16	1	1	1	6.502	n/a	7.167	1.991	1.745	3.74	2.341	n/a	n/a	n/a	0.678	n/a	1.032	n/a	0.1052
1E+07	16	1	1	1	8.202	n/a	8.467	2.323	2.441	3.594	1.434	n/a	n/a	n/a	1.532	n/a	2.006	n/a	0.6008
1E+07	16	1	1	1	4.804	n/a	4.618	2.822	2.309	3.423	1.21	n/a	n/a	n/a	1.339	n/a	1.02	n/a	0.0181

1E+07	17	2	2	2	7.735	2.578	6.116	4.107	2.722	3.882	1.162	2.556	3.278	1.058	0.654	0.809	0.829	1.068	0.074
1E+07	17	2	2	2	7.974	3.222	6.179	4.208	3.346	4.057	0.861	2.883	3.814	1.034	1.006	1.153	1.132	1.155	0.991
1E+07	17	2	2	2	15.202	5.977	6.055	4.665	2.582	2.645	1.182	3.522	3.548	0.898	0.627	0.494	0.897	1.048	0.0669
1E+07	17	1	1	1	15.462	n/a	10.424	2.355	2.742	5.51	2.802	n/a	n/a	n/a	1.094	n/a	1.092	n/a	0.3677
1E+07	17	1	1	1	7.599	n/a	9.606	2.434	2.478	4.328	2.088	n/a	n/a	n/a	0.654	n/a	0.918	n/a	0.2775
1E+07	17	1	1	1	10.612	n/a	8.967	2.451	2.053	3.902	2.15	n/a	n/a	n/a	0.476	n/a	0.793	n/a	0.1926
1E+07	17	1	1	1	6.851	n/a	8.789	2.566	2.464	3.827	1.364	n/a	n/a	n/a	0.477	n/a	0.904	n/a	0.1824
1E+07	17	1	1	1	15.321	n/a	11.591	2.421	1.907	4.59	2.742	n/a	n/a	n/a	0.998	n/a	1.23	n/a	0.4362
1E+07	17	1	1	1	9.136	n/a	11.738	2.556	2.706	5.413	2.569	n/a	n/a	n/a	0.957	n/a	1.247	n/a	0.4423
1E+07	17	1	1	1	17.665	n/a	11.771	2.706	2.468	4.137	1.539	n/a	n/a	n/a	0.633	n/a	1.068	n/a	0.3882
1E+07	17	1	1	1	12.447	n/a	12.27	2.826	2.366	4.626	2.123	n/a	n/a	n/a	1.519	n/a	1.632	n/a	0.5275
1E+07	17	1	1	1	13.335	n/a	11.774	2.77	2.069	4.178	2.135	n/a	n/a	n/a	0.944	n/a	1.465	n/a	0.4608
1E+07	17	1	1	1	7.627	n/a	9.545	2.313	2.196	3.858	1.787	n/a	n/a	n/a	0.696	n/a	1.096	n/a	0.283
1E+07	17	1	1	1	11.005	n/a	10.367	2.598	2.478	5.107	2.421	n/a	n/a	n/a	0.921	n/a	1.156	n/a	0.359
1E+07	17	1	1	1	8.103	n/a	10.43	2.746	2.645	4.247	1.726	n/a	n/a	n/a	0.847	n/a	1.317	n/a	0.3648
1E+07	17	1	1	1	9.146	n/a	10.644	2.814	2.645	4.016	1.411	n/a	n/a	n/a	0.712	n/a	1.33	n/a	0.3706
1E+07	17	1	1	1	9.684	n/a	8.109	2.181	2.464	4.093	1.837	n/a	n/a	n/a	0.925	n/a	1.167	n/a	0.1722
1E+07	17	1	1	1	7.468	n/a	9.658	2.742	2.451	3.328	1.434	n/a	n/a	n/a	1.038	n/a	1.267	n/a	0.3135
1E+07	17	1	1	1	15.002	n/a	6.67	2.029	2.069	3.335	1.417	n/a	n/a	n/a	0.938	n/a	0.995	n/a	0.0716
1E+07	17	1	1	1	6.789	n/a	7.674	2.421	2.366	4.008	1.837	n/a	n/a	n/a	0.9	n/a	1.147	n/a	0.1495
1E+07	17	1	1	1	5.838	n/a	6.374	2.029	2.069	3.106	1.034	n/a	n/a	n/a	0.988	n/a	1.044	n/a	0.0551
1E+07	17	1	1	1	7.115	n/a	8.917	2.894	2.241	4.163	1.823	n/a	n/a	n/a	0.618	n/a	0.955	n/a	0.2134
1E+07	17	1	1	1	15.093	n/a	7.447	2.521	2.057	5.198	3.003	n/a	n/a	n/a	0.793	n/a	0.996	n/a	0.1223
1E+07	17	1	1	1	9.656	n/a	6.829	2.53	1.924	2.992	0.811	n/a	n/a	n/a	0.829	n/a	1.151	n/a	0.0883
1E+07	17	1	1	1	15.983	n/a	7.175	2.903	2.358	4.341	1.868	n/a	n/a	n/a	1.217	n/a	1.204	n/a	0.1272
1E+07	17	1	1	1	12.63	n/a	6.394	2.67	2.556	4.057	1.607	n/a	n/a	n/a	0.858	n/a	1.033	n/a	0.0528
1E+07	17	1	1	1	7.474	n/a	8.291	3.485	2.397	3.09	1.417	n/a	n/a	n/a	1.037	n/a	1.324	n/a	0.203
1E+07	17	1	1	1	7.378	n/a	5.97	2.869	1.796	2.722	0.907	n/a	n/a	n/a	0.993	n/a	0.982	n/a	0.025
1E+07	17	1	1	1	2.802	n/a	6.333	3.218	2.742	4.137	1.545	n/a	n/a	n/a	0.382	n/a	0.832	n/a	0.0296
1E+07	17	1	1	1	10.606	n/a	7.322	2.976	2.578	4.746	2.566	n/a	n/a	n/a	0.823	n/a	1.19	n/a	0.1198
1E+07	17	1	1	1	9.275	n/a	12.318	2.053	3.084	4.773	1.787	n/a	n/a	n/a	1.939	n/a	1.647	n/a	0.5771
1E+07	17	1	1	1	9.436	n/a	11.235	2.116	2.817	4.65	1.787	n/a	n/a	n/a	1.853	n/a	2.075	n/a	0.6601
1E+07	17	1	1	1	12.902	n/a	11.286	3.367	2.358	4.868	2.84	n/a	n/a	n/a	1.295	n/a	1.464	n/a	0.4796
1E+07	17	1	1	1	15.174	n/a	12.19	1.924	3.028	5.236	2.358	n/a	n/a	n/a	1.925	n/a	2.064	n/a	0.6817
1E+07	17	1	1	1	13.733	n/a	11.324	2.355	2.722	5.75	2.954	n/a	n/a	n/a	2.081	n/a	2.129	n/a	0.6999
1E+07	17	1	1	1	11.927	n/a	11.923	2.697	2.438	3.995	1.561	n/a	n/a	n/a	1.704	n/a	1.913	n/a	0.5906
1E+07	17	1	1	1	15.205	n/a	10.602	2.41	2.718	4.099	1.502	n/a	n/a	n/a	2.133	n/a	1.955	n/a	0.6745
1E+07	17	1	1	1	8.104	n/a	12.604	2.928	3.028	4.698	1.673	n/a	n/a	n/a	1.541	n/a	1.933	n/a	0.5637
1E+07	17	1	1	1	10.757	n/a	12.329	2.894	3.084	4.731	1.801	n/a	n/a	n/a	1.237	n/a	1.77	n/a	0.5017
1E+07	17	1	1	1	12.18	n/a	13.01	3.106	2.617	4.424	1.782	n/a	n/a	n/a	2.213	n/a	2.36	n/a	0.7483
1E+07	17	1	1	1	12.175	n/a	10.174	2.434	2.645	4.71	1.924	n/a	n/a	n/a	2.421	n/a	1.943	n/a	0.7183
1E+07	17	1	1	1	14.542	n/a	11.302	3.042	3.122	5.316	2.185	n/a	n/a	n/a	1.777	n/a	1.996	n/a	0.6284
1E+07	18	1	1	1	7.462	n/a	7.876	2.069	2.645	4.378	1.801	n/a	n/a	n/a	1.296	n/a	1.228	n/a	0.1747
1E+07	18	1	1	1	7.076	n/a	10.161	2.869	2.123	3.952	1.907	n/a	n/a	n/a	0.792	n/a	1.186	n/a	0.3361
1E+07	18	1	1	1	6.133	n/a	8.191	2.313	2.029	3.882	2.053	n/a	n/a	n/a	0.766	n/a	1.001	n/a	0.1621
1E+07	18	1	1	1	12.05	n/a	10.34	3.019	2.697	4.265	1.622	n/a	n/a	n/a	1.054	n/a	1.38	n/a	0.3853
1E+07	18	1	1	1	11.323	n/a	12.102	2.181	2.582	4.163	1.726	n/a	n/a	n/a	0.819	n/a	1.086	n/a	0.4211
1E+07	18	1	1	1	6.698	n/a	12.385	2.341	2.775	4.997	2.527	n/a	n/a	n/a	1.461	n/a	1.605	n/a	0.5114
1E+07	18	1	1	1	11.478	n/a	10.655	2.069	2.521	4.392	1.929	n/a	n/a	n/a	1.196	n/a	1.201	n/a	0.412
1E+07	18	1	1	1	8.555	n/a	10.958	2.274	2.355	5.195	2.849	n/a	n/a	n/a	1.756	n/a	1.911	n/a	0.6111
1E+07	18	1	1	1	7.249	n/a	10.338	2.355	2.057	3.979	2.088	n/a	n/a	n/a	0.748	n/a	0.976	n/a	0.3247
1E+07	18	1	1	1	8.304	n/a	11.52	2.817	2.284	4.008	1.924	n/a	n/a	n/a	0.601	n/a	0.946	n/a	0.3823
1E+07	18	1	1	1	11.722	n/a	10.345	2.578	2.323	4.89	2.706	n/a	n/a	n/a	0.9	n/a	1.152	n/a	0.3561
1E+07	18	1	1	1	11.418	n/a	12.112	3.132	2.598	4.265	1.929	n/a	n/a	n/a	1.313	n/a	1.173	n/a	0.4577
1E+07	18	1	1	1	9.406	n/a	10.994	2.869	2.366	4.79	2.295	n/a	n/a	n/a	1.041	n/a	1.171	n/a	0.4031
1E+07	18	1	1	1	4.81	n/a	8.433	2.284	2.207	3.808	1.673	n/a	n/a	n/a	0.528	n/a	0.982	n/a	0.1671
1E+07	18	1	1	1	6.822	n/a	10.132	2.926	2.556	3.747	1.721	n/a	n/a	n/a	0.854	n/a	1.123	n/a	0.3304
1E+07	18	1	1	1	6.897	n/a	7.676	2.697	1.924	2.84	1.034	n/a	n/a	n/a	1.011	n/a	1.204	n/a	0.1595
1E+07	18	1	1	1	6.553	n/a	6.168	2.569	2.313	3.33	1.058	n/a	n/a	n/a	1.063	n/a	1.101	n/a	0.0412
1E+07	18	1	1	1	6.774	n/a	6.446	2.976	2.185	3.288	1.496	n/a	n/a	n/a	0.946	n/a	0.991	n/a	0.0622
1E+07	18	1	1	1	6.581	n/a	5.697	2.742	2.849	4.099	1.496	n/a	n/a	n/a	0.877	n/a	1.135	n/a	0.0204
1E+07	18	1	1	1	7.566	n/a	8.258	2.004	2.984	3.85	1.248	n/a	n/a	n/a	1.836	n/a	1.537	n/a	0.5704
1E+07	18	1	1	1	8.261	n/a	11.339	2.826	2.566	4.749	2.241	n/a	n/a	n/a	1.012	n/a	1.657	n/a	0.4733
1E+07	18	1	1	1	8.742	n/a	9.184	2.632	2.566	4.902	2.491	n/a	n/a	n/a	1.698	n/a	2.013	n/a	0.6146
1E+07	18	1	1	1	14.59	n/a	10.901	1.958	2.556	4.384	1.745	n/a	n/a	n/a	2.344	n/a	1.909	n/a	0.7035

1E+07	18	1	1	1	9.516	n/a	11.836	2.697	2.883	5.396	2.697	n/a	n/a	n/a	2.353	n/a	2.403	n/a	0.7673
1E+07	18	1	1	1	7.202	n/a	10.232	2.464	2.754	4.733	1.907	n/a	n/a	n/a	1.976	n/a	1.806	n/a	0.6215
1E+07	18	1	1	1	14.413	n/a	11.849	2.869	2.632	5.353	2.546	n/a	n/a	n/a	1.713	n/a	2.365	n/a	0.6889
1E+07	18	1	1	1	18.734	n/a	11.603	3.1	2.718	4.705	2.069	n/a	n/a	n/a	1.774	n/a	2.263	n/a	0.6853
1E+07	18	1	1	1	14.981	n/a	10.843	2.954	2.976	5.107	2.274	n/a	n/a	n/a	1.809	n/a	1.985	n/a	0.6354
1E+07	18	1	1	1	11.914	n/a	10.609	3.613	2.926	5.385	2.468	n/a	n/a	n/a	1.982	n/a	1.916	n/a	0.6566
1E+07	19	1	2	2	15.041	3.678	7.724	3.298	2.468	3.485	0.907	n/a	5.107	2.928	1.989	n/a	1.259	0.945	0.9865
1E+07	19	2	2	2	10.423	4.832	5.446	5.234	2.697	3.09	1.154	3.85	3.814	0.925	0.602	0.733	1.105	0.988	0.9821
1E+07	19	1	1	1	13.014	n/a	10.826	2.185	2.241	4.362	2.241	n/a	n/a	n/a	1.023	n/a	1.276	n/a	0.406
1E+07	19	1	1	1	14.406	n/a	10.174	2.057	2.341	4.422	2.196	n/a	n/a	n/a	0.664	n/a	1.204	n/a	0.3219
1E+07	19	1	1	1	15.942	n/a	9.206	2.53	2.244	4.99	2.775	n/a	n/a	n/a	0.785	n/a	0.812	n/a	0.2451
1E+07	19	1	1	1	7.832	n/a	6.318	1.787	2.441	4.053	1.721	n/a	n/a	n/a	0.464	n/a	0.979	n/a	0.0342
1E+07	19	1	1	1	8.594	n/a	9.574	2.826	2.241	3.218	0.977	n/a	n/a	n/a	0.961	n/a	1.338	n/a	0.3051
1E+07	19	1	1	1	2.011	n/a	8.892	2.727	2.366	4.091	1.726	n/a	n/a	n/a	1.054	n/a	1.464	n/a	0.2558
1E+07	19	1	1	1	14.419	n/a	8.879	2.742	1.942	3.542	1.787	n/a	n/a	n/a	0.675	n/a	1.05	n/a	0.2213
1E+07	19	1	1	1	12.749	n/a	9.938	1.364	2.104	5.274	3.186	n/a	n/a	n/a	0.757	n/a	1.05	n/a	0.2968
1E+07	19	1	1	1	6.611	n/a	9.976	1.451	2.421	4.288	2.004	n/a	n/a	n/a	0.62	n/a	1.06	n/a	0.2913
1E+07	19	1	1	1	11.947	n/a	13.861	2.546	2.54	4.91	2.645	n/a	n/a	n/a	1.323	n/a	1.542	n/a	0.4986
1E+07	19	1	1	1	2.962	n/a	7.638	1.539	2.569	4.242	1.962	n/a	n/a	n/a	0.635	n/a	1.277	n/a	0.1445
1E+07	19	1	1	1	15.556	n/a	8.442	1.907	1.907	3.33	1.434	n/a	n/a	n/a	0.598	n/a	1.19	n/a	0.1773
1E+07	19	1	1	1	11.03	n/a	9.195	2.181	2.088	4.856	2.645	n/a	n/a	n/a	1.046	n/a	1.201	n/a	0.2721
1E+07	19	1	1	1	5.727	n/a	8.915	2.135	2.645	4.107	1.539	n/a	n/a	n/a	0.869	n/a	1.234	n/a	0.2424
1E+07	19	1	1	1	8.358	n/a	7.558	1.837	2.123	3.288	1.217	n/a	n/a	n/a	0.95	n/a	1.358	n/a	0.147
1E+07	19	1	1	1	11.745	n/a	10.654	2.84	1.837	4.634	2.953	n/a	n/a	n/a	0.921	n/a	1.144	n/a	0.3736
1E+07	19	1	1	1	17.237	n/a	8.882	2.451	2.434	5.278	2.817	n/a	n/a	n/a	0.763	n/a	1.173	n/a	0.2318
1E+07	19	1	1	1	10.87	n/a	6.157	1.837	2.004	3.186	1.002	n/a	n/a	n/a	0.516	n/a	0.936	n/a	0.0227
1E+07	19	1	1	1	12.574	n/a	7.322	2.632	2.181	3.367	1.382	n/a	n/a	n/a	0.779	n/a	1.152	n/a	0.1174
1E+07	19	1	1	1	6.494	n/a	5.53	2.309	2.029	2.926	0.898	n/a	n/a	n/a	0.962	n/a	0.884	n/a	0.0159
1E+07	19	1	1	1	12.542	n/a	6.311	3.05	2.41	3.983	1.545	n/a	n/a	n/a	1.421	n/a	0.903	n/a	0.0598
1E+07	19	1	1	1	13.115	n/a	7.176	2.004	2.295	3.788	1.434	n/a	n/a	n/a	1.124	n/a	1.307	n/a	0.1247
1E+07	19	1	1	1	7.734	n/a	10.316	2.77	2.073	4.409	2.569	n/a	n/a	n/a	1.586	n/a	1.722	n/a	0.5439
1E+07	19	1	1	1	11.873	n/a	11.846	2.556	2.954	4.746	1.924	n/a	n/a	n/a	2.055	n/a	1.951	n/a	0.6781
1E+07	19	1	1	1	5.506	n/a	7.569	3.338	1.991	3.849	1.745	n/a	n/a	n/a	1.769	n/a	2.215	n/a	0.9776
1E+07	20	1	1	1	10.145	n/a	10.598	1.942	1.687	4.265	2.569	n/a	n/a	n/a	0.583	n/a	0.897	n/a	0.3276
1E+07	20	1	1	1	7.766	n/a	9.593	1.801	2.366	3.423	1.182	n/a	n/a	n/a	0.862	n/a	1.348	n/a	0.294
1E+07	20	1	1	1	5.701	n/a	9.598	2.244	2.295	3.674	1.434	n/a	n/a	n/a	0.619	n/a	0.966	n/a	0.2748
1E+07	20	1	1	1	8.276	n/a	10.632	2.491	2.313	4.37	2.088	n/a	n/a	n/a	1.207	n/a	1.392	n/a	0.4241
1E+07	20	1	1	1	6.971	n/a	8.656	2.123	2.284	3.502	1.308	n/a	n/a	n/a	0.903	n/a	1.255	n/a	0.2265
1E+07	20	1	1	1	8.567	n/a	9.682	3.215	2.984	5.109	2.464	n/a	n/a	n/a	0.684	n/a	1.175	n/a	0.2885
1E+07	20	1	1	1	12.384	n/a	10.906	1.787	2.67	4.536	1.907	n/a	n/a	n/a	1.233	n/a	1.407	n/a	0.4454
1E+07	20	1	1	1	14.084	n/a	10.52	2.185	1.814	4.235	2.295	n/a	n/a	n/a	0.865	n/a	0.9	n/a	0.3475
1E+07	20	1	1	1	8.692	n/a	10.173	2.185	2.358	4.313	2.15	n/a	n/a	n/a	1.138	n/a	1.566	n/a	0.418
1E+07	20	1	1	1	13.855	n/a	11.046	2.478	2.181	3.859	1.745	n/a	n/a	n/a	0.773	n/a	1.056	n/a	0.3794
1E+07	20	1	1	1	7.627	n/a	10.503	2.441	2.341	4.282	1.942	n/a	n/a	n/a	0.95	n/a	1.121	n/a	0.3619
1E+07	20	1	1	1	8.317	n/a	10.716	2.569	2.181	4.506	2.366	n/a	n/a	n/a	1.396	n/a	1.351	n/a	0.4702
1E+07	20	1	1	1	7.171	n/a	6.011	2.834	1.996	2.53	0.898	n/a	n/a	n/a	0.957	n/a	0.97	n/a	0.0273
1E+07	20	1	1	1	7.718	n/a	6.388	3.003	2.53	4.328	1.962	n/a	n/a	n/a	1.604	n/a	2.264	n/a	0.9732
1E+07	20	1	1	1	6.306	n/a	7.103	2.313	2.397	3.574	1.263	n/a	n/a	n/a	0.936	n/a	1.142	n/a	0.1076
1E+07	20	1	1	1	10.779	n/a	6.152	2.546	2.438	3.21	0.642	n/a	n/a	n/a	1.194	n/a	1.129	n/a	0.0458
1E+07	20	1	1	1	12.784	n/a	11.792	3.27	2.869	4.885	2.135	n/a	n/a	n/a	2.575	n/a	1.832	n/a	0.7257
1E+07	20	1	1	1	7.628	n/a	5.48	1.991	1.721	3.594	1.814	n/a	n/a	n/a	0.78	n/a	1.082	n/a	0.0136
1E+07	20	1	1	2	9.374	0.581	8.898	3.335	2.77	4.43	1.673	n/a	n/a	n/a	2.38	n/a	2.33	n/a	0.8999
1E+07	21	1	2	2	6.926	2.578	6.487	4.145	2.073	3.374	1.434	n/a	3.006	1.545	2.814	n/a	0.975	1.08	0.921
1E+07	21	1	1	1	10.942	n/a	9.73	2.386	1.894	3.747	1.907	n/a	n/a	n/a	1.065	n/a	1.154	n/a	0.3107
1E+07	21	1	1	1	6.031	n/a	8.223	2.073	2.241	3.674	1.388	n/a	n/a	n/a	1.07	n/a	1.048	n/a	0.1798
1E+07	21	1	1	1	7.282	n/a	8.784	2.309	2.274	4.018	1.787	n/a	n/a	n/a	0.988	n/a	1.039	n/a	0.2292
1E+07	21	1	1	1	12.416	n/a	10.921	1.339	2.27	4.414	2.123	n/a	n/a	n/a	0.991	n/a	1.051	n/a	0.3765
1E+07	21	1	1	1	12.071	n/a	11.302	2.214	2.53	4.194	1.711	n/a	n/a	n/a	0.986	n/a	1.069	n/a	0.4001
1E+07	21	1	1	1	12.665	n/a	12.488	2.594	2.341	6.163	3.72	n/a	n/a	n/a	1.041	n/a	1.316	n/a	0.4546
1E+07	21	1	1	1	7.942	n/a	10.43	2.569	1.929	4.378	2.491	n/a	n/a	n/a	0.879	n/a	1.048	n/a	0.3533
1E+07	21	1	1	1	9.344	n/a	7.668	2.073	1.85	3.613	1.929	n/a	n/a	n/a	0.609	n/a	0.879	n/a	0.1395
1E+07	21	1	1	1	8.609	n/a	10.014	2.962	2.569	4.62	1.929	n/a	n/a	n/a	1.077	n/a	1.816	n/a	0.4301
1E+07	21	1	1	1	10.037	n/a	6.996	2.088	2.313	3.207	0.907	n/a	n/a	n/a	0.871	n/a	1.041	n/a	0.0931
1E+07	21	1	1	1	5.781	n/a	7.058	2.421	2.116	3.522	1.29	n/a	n/a	n/a	0.687	n/a	1.273	n/a	0.1028
1E+07	21	1	1	1	9.23	n/a	12.774	2.438	2.295	4.131	1.721	n/a	n/a	n/a	1.96	n/a	1.723	n/a	0.5974

1E+07	21	1	1	1	12.482	n/a	11.998	2.598	2.706	4.519	1.684	n/a	n/a	n/a	1.11	n/a	2.034	n/a	0.5243
1E+07	21	1	1	1	10.803	n/a	10.778	2.366	2.826	4.649	1.745	n/a	n/a	n/a	1.67	n/a	1.986	n/a	0.6077
1E+07	21	1	1	1	9.434	n/a	11.812	2.642	2.582	4.178	1.539	n/a	n/a	n/a	0.98	n/a	1.564	n/a	0.4516
1E+07	21	1	1	1	4.694	n/a	6.101	1.907	2.468	3.613	1.539	n/a	n/a	n/a	1.09	n/a	1.145	n/a	0.0388
1E+07	21	1	1	2	8.444	0.165	11.025	2.057	2.763	5.413	2.869	n/a	n/a	n/a	1.879	n/a	1.52	n/a	0.5604
1E+07	21	1	1	1	4.654	n/a	9.184	2.029	2.207	4.786	1.837	n/a	n/a	n/a	2.003	n/a	1.549	n/a	0.594
1E+07	21	1	1	2	12.364	0.062	12.298	2.849	2.645	5.359	2.566	n/a	n/a	n/a	2.613	n/a	2.127	n/a	0.7635
1E+07	21	1	1	1	17.33	n/a	11.969	3.084	2.594	4.761	2.192	n/a	n/a	n/a	1.916	n/a	1.847	n/a	0.6319
1E+07	21	1	1	1	11.658	n/a	10.092	2.678	2.817	4.65	1.907	n/a	n/a	n/a	2.46	n/a	2.056	n/a	0.7295
1E+07	21	1	1	1	14.255	n/a	11.179	3.019	2.617	4.235	1.787	n/a	n/a	n/a	1.633	n/a	1.707	n/a	0.5504
1E+07	21	1	1	2	9.969	0.431	10.807	2.962	2.556	3.979	1.55	n/a	n/a	n/a	2.016	n/a	2.553	n/a	0.7407
1E+07	21	1	1	1	10.858	n/a	6.171	2.645	2.135	3.028	1.411	n/a	n/a	n/a	0.991	n/a	1.342	n/a	0.0481
